# KDM6A loss enhances oxidative phosphorylation uncovering tissue-level convergent evolution

**DOI:** 10.1038/s44318-026-00891-0

**Published:** 2026-09-04

**Authors:** Antonino Alex Cartalemi, Dalia Rosano, Camilla Ferrari, Gemma Crupi, Laura Cassina, Guido Gatti, Federica Corigliano, Simona Punzi, Ilaria Villanti, Daphné Dupéré-Richer, Chiara D’Ercole, Veronica Ruggieri, Annarita Miluzio, Tiziana Bruno, Jose Manuel Garcia Manteiga, Valentina Giansanti, Richard L Bennett, Davide Cittaro, Luca Madaro, Alessandra Boletta, Telmo Pievani, Stefano Biffo, Maurizio Fanciulli, Michele Ciboddo, Jonathan Licht, Giovanni Tonon

**Affiliations:** 1 Functional Genomics of Cancer Unit, Comprehensive Cancer Center, IRCCS San Raffaele Scientific Institute, Milan, Italy; 2 Vita-Salute San Raffaele University, Milan, Italy; 3 Cystic Kidney Disorders Unit, Genetics and Cell Biology Division, IRCCS San Raffaele Scientific Institute, Milan, Italy; 4 Department of Experimental Medicine, TOR, University of Rome Tor Vergata, Rome, Italy; 5 Department of Epigenetics, Van Andel Institute, Grand Rapids, MI USA; 6 Department of Anatomical, Histological, Forensic Sciences and Orthopedics, Sapienza University of Rome, Rome, Italy; 7 Istituto Nazionale di Genetica Molecolare, INGM, “Romeo ed Enrica Invernizzi”, Milan, Italy; 8 Gene Expression and Cancer Models Unit, Department of Research and Advanced Technologies Translational Research Area, IRCCS Regina Elena National Cancer Institute, Rome, Italy; 9 Center for Omics Sciences, IRCCS San Raffaele Scientific Institute, Milan, Italy; 10 Department of Biology, University of Padua, Padua, Italy; 11 Department of Biosciences, University of Milan, Milan, Italy

**Keywords:** Cancer, Metabolism, Organelles

## Abstract

The tumor suppressor *KDM6A*/*UTX*, a histone demethylase and a 2-oxoglutarate-dependent dioxygenase, is frequently lost in many cancer types. We show that *KDM6A* loss pervasively activates oxidative phosphorylation in several solid tumors, generating a pseudo-hyperoxic environment, opposite from the pseudo-hypoxia observed in VHL-mutated renal carcinomas. Mechanistically, KDM6A sustains the expression of the coil-coil domain gene *CCDC3*, which inhibits CREB1-driven transcription of the mitochondrial regulator PPARGC1A. In the hematological cancer multiple myeloma where *KDM6A* is frequently deleted, its loss similarly promotes oxidative phosphorylation, but via an alternative mechanism: the increased transfer of mitochondria from stromal to myeloma cells via tunneling nanotubes, triggered by the loss of the mTORC1 inhibitor *TRAF3IP3*. Beyond cancer, KDM6A regulates oxidative phosphorylation also during development and in adult tissues, engaging either the CCDC3-CREB1 or the TRAF3IP3-mTORC1 pathways. These mutually exclusive associations suggest a tissue-level convergent evolution, positioning *KDM6A* as a central modulator of mitochondrial activity through context-specific partners.

## Synopsis

Loss of the oxygen-sensing chromatin regulator KDM6A/UTX is frequent in cancer cells. Here, this is shown to promote a pseudo-hyperoxic metabolic state through alternative tissue-specific pathways for controlling oxidative phosphorylation, highlighting tissue-level convergent evolution as a mechanism of metabolic regulation.

- Loss of KDM6A activates oxidative phosphorylation (OXPHOS) across multiple solid tumor types and increases mitochondrial mass.
- In solid tumors, KDM6A restrains OXPHOS through a CCDC3-CREB1-PPARGC1A pathway controlling mitochondrial biogenesis.
- In multiple myeloma, KDM6A loss activates mTORC1 through TRAF3IP3 repression and promotes mitochondrial transfer from stromal cells via tunneling nanotubes.
- KDM6A-dependent regulation of OXPHOS also operates in normal tissues and during development.

## Abstract (web summary)

Loss of the oxygen-sensing chromatin regulator KDM6A drives a pseudo-hyperoxic metabolic state through two pathways, one based on increased mitochondria synthesis, and another via increased mitochondria trafficking from stromal cells to cancer cells.

## Graphical abstract

**Figure:**
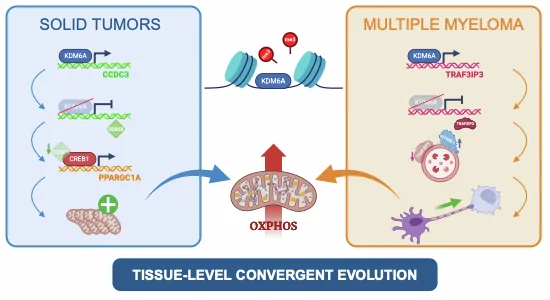

## Introduction

KDM6A (lysine demethylase 6a), also known as UTX (ubiquitously transcribed tetratricopeptide repeat on chromosome X), is a 1401 amino acid histone demethylase (Tran et al, 2020) that works in concert with the MLL3/4 histone H3K4 methyltransferase complex (Cho et al, 2007) to activate gene transcription, counteracting the activity of the Polycomb repressive complex (PRC) (Lee et al, 2007). KDM6A removes the repressive di- and tri-methylation residues from histone lysine H3K27 and recruits histone acetyl transferases, thus unleashing gene expression (Agger et al, 2007; Wang et al, 2017; Dupéré-Richer et al, 2024; Pacella et al, 2025). KDM6A exerts a prominent role during development. For example, KDM6A removes methyl groups from H3K27 in proximity of HOX gene promoters contributing to their transcription during animal body patterning (Bracken et al, 2006). Besides its role during development, KDM6A is engaged also in tissue regeneration, as for example in muscle satellite cells (Faralli et al, 2016).

KDM6A belongs to a class of enzymes sensing oxygen levels, a key function both in animals and plants (Hammarlund et al, 2020; Losman et al, 2020). In fact, KDM6A 2-oxoglutarate-dependent dioxygenase (2OGDD) activity is required for its demethylase function (McDonough et al, 2010; Kooistra and Helin, 2012). 2OGDDs include for example EGLN prolyl 4-hydroxylases (also known as PHDs), and the FIH1 asparaginyl hydroxylases. EGLN proteins control hypoxia-inducible transcription factors HIF1α and HIF2α: in normoxic conditions, they hydroxylate HIFα, hence promoting its binding to the von Hippel–Lindau tumor suppressor protein (pVHL), leading to HIFα ubiquitination (Ivan and Kaelin, 2017). The pervasive loss of pVHL in clear cell renal cancer leads to a pseudo-hypoxic state in these tumors, elicited by the persistent activation of HIF1α and HIF2α, despite normal levels of O_2_. The enhanced expression of HIF-target genes then triggers oncogenesis (Semenza, 2003). Intriguingly, KDM6A role in differentiation is oxygen-dependent, albeit in a HIF-independent manner (Chakraborty et al, 2019). Specifically, hypoxia inhibits KDM6A thus promoting H3K27 methylation and interfering with cell fate determination.

O_2_ and nutrients are essential for cell survival, and their levels are carefully sensed and assessed by cells, leading to a meticulous regulation of growth, proliferation, and energy production. The AMPK (AMP-activated Protein Kinase) and mTOR (mechanistic Target Of Rapamycin) pathways are the central molecular hubs that orchestrate the cellular responses to nutrient availability and needs, through the monitoring of ATP levels (González et al, 2020). The serine/threonine kinase AMPK detects the ratios between AMP/ATP and ADP/ATP, thus gauging ATP availability. Upon AMP:ATP and ADP:ATP unbalance, with reduction on ATP, AMPK induces a compensatory increase of oxidative phosphorylation (OXPHOS) by activating the transcriptional coactivator PGC-1α (Jäger et al, 2007) (encoded by the *PPARGC1A* gene), responsible for mitochondria biogenesis (González et al, 2020). Conversely, mTOR Complex 1 (mTORC1) senses amino acids and growth factors levels, and induces anabolism (Sabatini et al, 1994), usually activating glycolysis over OXPHOS through the phosphorylation of various targets including p70S6 kinase 1 (S6K1) and eIF4E-binding protein (4EBP) (Saxton and Sabatini, 2017).

Rewiring of cell metabolism is a recognized hallmark of tumor cells (Pavlova and Thompson, 2016). Cancer often intensifies glycolysis in the absence of increased mitochondrial oxygen consumption, a phenomenon defined “Warburg effect” (Warburg, 1924). However, evidence is mounting that OXPHOS itself, elicited by an increased number of functional mitochondria, is powerfully oncogenic per se (Sica et al, 2020), as for example in chronic myeloid leukemia, triple negative breast cancer and non-small cell lung cancer (Kuntz et al, 2017; Lee et al, 2017; Lissanu Deribe et al, 2018; Rao et al, 2019).

Our group first reported that *KDM6A* is a tumor suppressor genetically altered in various tumor types, including the incurable hematological cancer multiple myeloma (MM), a malignant clonal expansion of plasma cells, where it is often deleted (Van Haaften et al, 2009). MM cells tightly rely on the surrounding bone marrow microenvironment, which fosters MM cell growth, survival, drug resistance and migration (Hideshima et al, 2007). In MM, we reported that *KDM6A* loss enhances tumorigenicity and sensitizes MM cells to EZH2 inhibitors (Ezponda et al, 2017). Moreover, *KDM6A* regulates MM immunogenicity activating the transcription of *NLRC5* and *CIITA*, the master regulators of the major histocompatibility complex (Dupéré-Richer et al, 2024).

*KDM6A* is also one of the most frequently altered tumor suppressors in many solid tumor types, where frameshift, nonsense, splice site, and missense mutations are spread across the gene coding region (Wang and Shilatifard, 2019). Urothelial bladder carcinoma presents the highest *KDM6A* mutation frequency (up to 30%). Despite its relevance, relatively little is known on its oncogenic role, with most studies focusing on its activities on proliferation control. For instance, in lung adenocarcinoma *KDM6A* loss was associated with the downregulation of the tumor suppressors *CDKN2A* and *CDKN2B* (Wu et al, 2018). *KDM6A* KO in pancreas reconfigures chromatin and activates super-enhancers, thus controlling the transcription of *Myc*, *∆Np63* and *Runx3*, inducing a squamous-like metastatic pancreatic cancer (Andricovich et al, 2018). In bladder cancer, *KDM6A* loss leads to repression of EZH2 target genes including tumor suppressor proteins and increased sensitivity to EZH2 inhibitors (Ler et al, 2017), while in breast cancer cell lines *KDM6A* loss induces epithelial to mesenchymal transition (EMT) through up-regulation of key EMT regulators *SNAIL* and *ZEB1/2* (Choi et al, 2015).

As KDM6A is one of the few reported oxygen-sensing proteins (Hammarlund et al, 2020), in this study we sought to determine whether KDM6A exerts a role in cancer through modulation of oxygen metabolism.

## Results

### KDM6A loss induces OXPHOS through mitochondrial mass increase in solid tumors

**Figure EV1:**
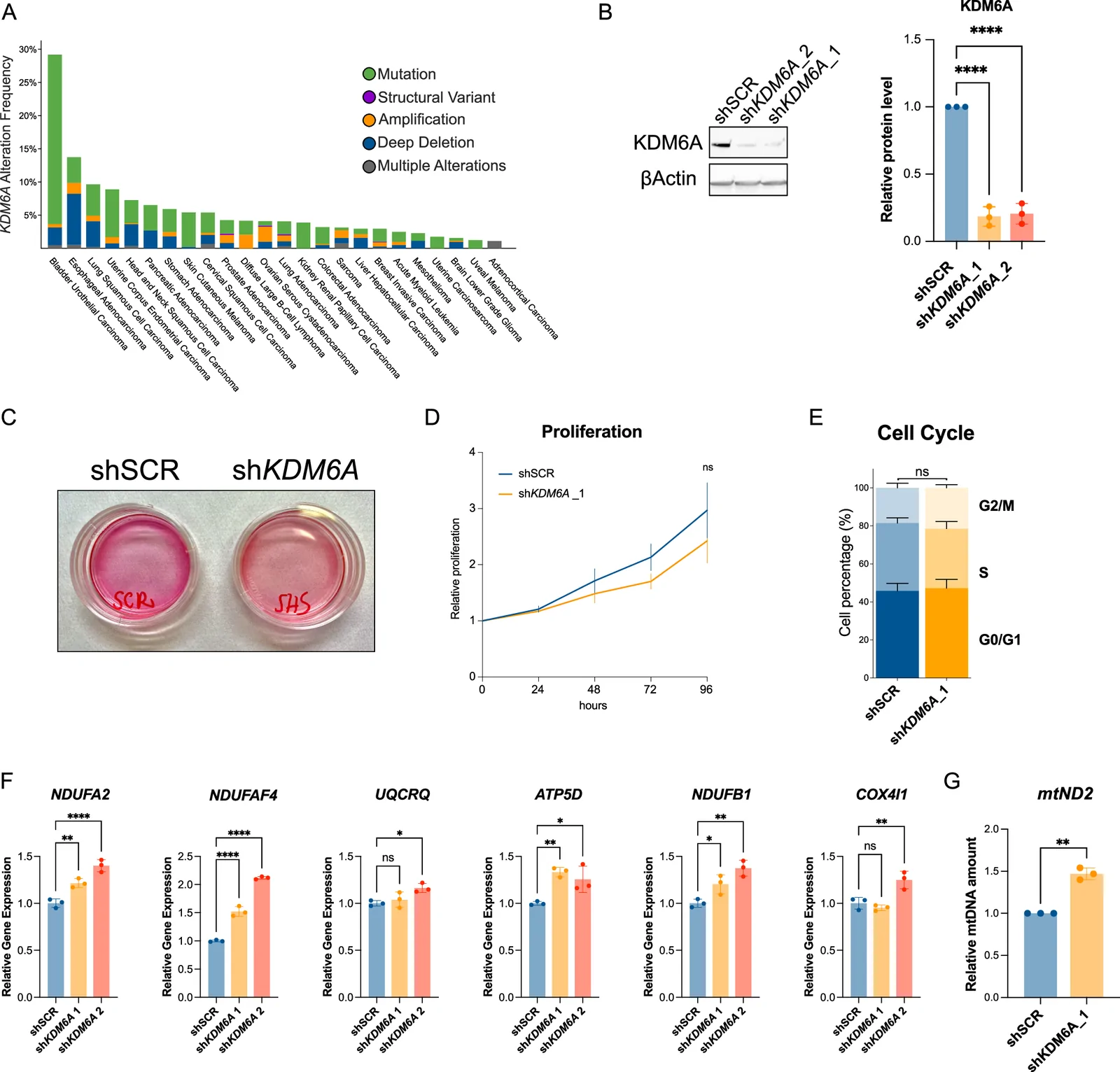
The loss od KDM6A increases medium acidification without perturbing proliferation and cell cycle but increasing ETC genes expression. (**A**) *KDM6A* alteration frequency from several patient datasets from TCGA, PanCancer Atlas (minimum frequency > 1%). Bar chart generated in cBioPortal browser. (**B**) One representative out of three western blot of KDM6A protein level on shSCR (scrambled short hairpin RNA) and sh*KDM6A* conditions. Normalization on βActin and shSCR. Statistical significance was assessed by applying one-way ANOVA test. Mean +/− SD, *n* = 3 (****p = 0.0000032, *p* = 0.0000037). (**C**) Representative images of cell culture media acidification of U-2 OS shSCR compared to U-2 OS *KDM6A* KD. (**D**) Relative proliferation at 24, 48, 72, and 96 h of shSCR and *KDM6A* KD cells of three independent biological replicates. Mean +/− SEM. Unpaired t-test (ns *p* = 0.4352). (**E**) Cell cycle phases, percentage of shSCR and sh*KDM6A* cells. Data are represented as mean +/− SD of four independent biological replicates. Statistical significance was assessed by applying two-way ANOVA test (ns *p* = 0.533). (**F**) Gene expression of ETC genes on shSCR and sh*KDM6A* conditions evaluated by RT-qPCR. Data are represented as mean +/− SD of three independent biological replicates. Statistical significance was assessed by applying one-way ANOVA test (*NDUFA2* **p = 0.002751, ****p = 0.000098; *NDUFAF4* ****p = 0.0000212, ****p = 0.0000002; *UQCRQ* ns *p* = 0.4587, *p = 0.0135; *ATP5D* **p = 0.0034, *p = 0.0113; *NDUFB1* *p = 0.0218, **p = 0.0013; *COX4I1* ns *p* = 0.4038, **p = 0.004). (**G**) Abundance of mtDNA (*mtND2*) assessed by qPCR. Data are normalized to *NBPF9* and to shSCR condition and represented as mean +/− SD of three independent biological replicates. Significant differences were quantified by one sample t-test (**p = 0.0073).

**Figure 1:**
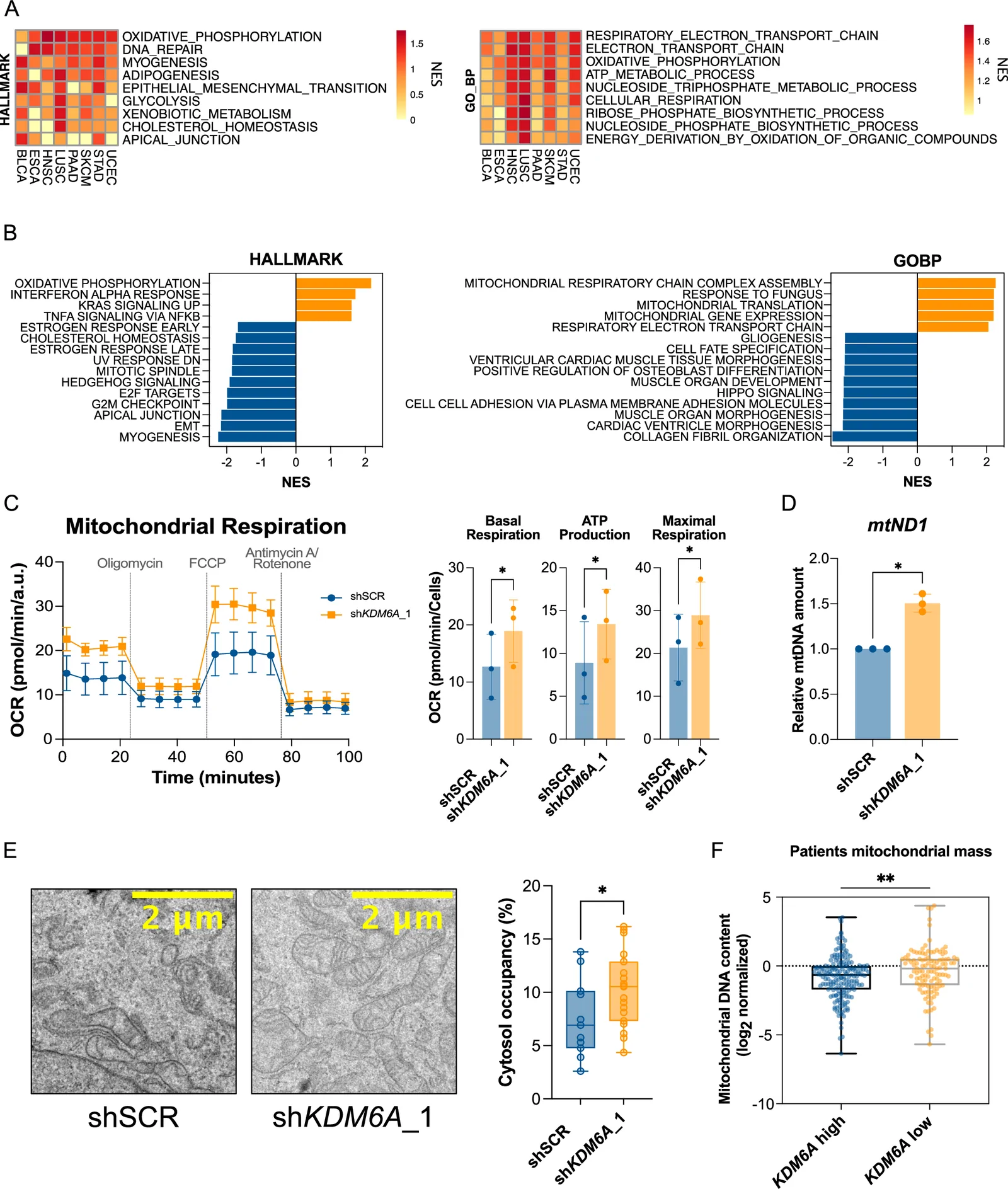
Transcriptional and functional enhancement of OXPHOS is associated with increased mitochondria in solid tumors upon KDM6A loss. (**A**) Top 8 tumors ranked by *KDM6A* alteration frequency selected for transcriptomics analysis by GSEA (v4.3.2). Positive NES indicates enriched pathways in *KDM6A* low expression patients (lower quartile). Pathways were derived from HALLMARK and Gene Ontology Biological Processes (GO_BP) (v2022.1). TCGA patient dataset: Urothelial Bladder Carcinoma (BLCA), Esophageal Carcinoma (ESCA), Head-Neck Squamous Cell Carcinoma (HNSC), Lung Squamous Cell Carcinoma (LUSC), Pancreatic Adenocarcinoma (PAAD), Skin Cutaneous Melanoma (SKCM), Stomach Adenocarcinoma (STAD), Uterine Corpus Endometrial Carcinoma (UCEC). (**B**) GSEA (v4.3.2) of differentially expressed genes upon *KDM6A* knock-down in U-2 OS cell line from RNAseq data. Pathways derived from HALLMARK and Gene Ontology Biological Processes (GOBP). (**C**) One representative analysis out of three independent biological replicates of OCR measurements in U-2 OS cell line in shSCR and sh*KDM6A* conditions, after sequential addition of oligomycin, FCCP and antimycin/rotenone. Bar plots of Basal Respiration, ATP Production and Maximal Respiration from three independent biological replicates (mean, +/− SEM). Significant differences were quantified by two-tailed paired Student’s t-test (*p = 0.0451, *p* = 0.0401, *p* = 0.0389). (**D**) Abundance of mtDNA (*mtND1*) in U-2 OS assessed by qPCR. Data are normalized to *NBPF9* and to shSCR condition and are represented as mean +/− SD of three independent biological replicates. Significant differences were quantified by one sample t-test (*p = 0.0124). (**E**) Transmission Electron Microscopy (TEM) representative images in shSCR or sh*KDM6A* U-2 OS cell line. Scale bar: 2 µm. Box-plot showing the cytosolic occupancy of mitochondria in shSCR (*n* = 11, Min = 2.61, Q1 = 4.78, Median=6.93, Q3 = 10.12, Max = 13.8) and sh*KDM6A* (*n* = 19, Min = 4.36, Q1 = 7.33, Median = 10.54, Q3 = 12.86, Max = 16.18). Significant differences were quantified by one-tailed Unpaired Student’s t-test (*p = 0.0277). (**F**) Box-plot representation of mitochondrial mass comparison between *KDM6A* high (upper quartile, *n* = 183, Min = −6.363, Q1 = −1.698, Median = −0.6493, Q3 = −0.00675, Max=3.537) and low expression (lower quartile, *n* = 114, Min = −5.687, Q1 = −1.373, Median = −0.1779, Q3 = 0.5041, Max = 4.384) patients from TCGA dataset. Samples were analyzed through normality and log-normality tests. Significant differences were quantified by Man-Whitney test (**p = 0.0047). Source data are available online for this figure.

To identify a role for *KDM6A* in the cancer types where it is most frequently altered (Fig. EV1A), we leveraged the TCGA PanCancer Atlas (Hoadley et al, 2018). Collating the data from these tumor types, we found that pathways related to OXPHOS were the most pervasively upregulated in patient samples with low *KDM6A* expression, in all the tumors analyzed (Fig. 1A). To validate these results, we knocked down *KDM6A* expression by RNA interference in U-2 OS, a cancer cell line widely used for testing cancer phenotypes (Fig. EV1B). To our surprise, the cell culture media in *KDM6A* knockdown (KD) cells was indeed more acidified than the wild-type cells (Fig. EV1C), potentially associated to an increase in carbonic acid arising from OXPHOS (Mookerjee et al, 2015). This phenotype was not associated with a proliferation advantage (Fig. EV1D) nor altered cell-cycle (Fig. EV1E). RNAseq analysis on these cells confirmed that the most strongly upregulated pathways upon KDM6A loss again belonged to OXPHOS and mitochondria-related genes (Fig. 1B), correlating with the phenotype detected in the TCGA data. Quantitative reverse transcription PCR (RT-qPCR) analysis confirmed that the genes involved in the electron transport chain (ETC) were upregulated upon *KDM6A* KD (Fig. EV1F). Seahorse Mito Stress Test was performed to determine if *KDM6A* KD led to the functional activation of OXPHOS. Indeed, cells depleted for *KDM6A* displayed a higher oxygen consumption rate (OCR), with a significant increment in basal respiration, ATP production, and maximal respiration (Fig. 1C). Furthermore, mitochondrial DNA (mtDNA) (Figs. 1D and EV1G) and electron microscopy (cytosol occupancy; Fig. 1E) revealed that mitochondrial mass was robustly increased upon *KDM6A* KD. Finally, we analyzed published mtDNA quantification data from several cancer patients samples (Reznik et al, 2016). This analysis revealed that samples with low *KDM6A* expression presented higher mitochondrial DNA content (Fig. 1F). Collectively, these data provide evidence that KDM6A loss increases mitochondrial mass and activity.

### PPARGC1A is upregulated to induce mitochondria biogenesis upon *KDM6A* KD

**Figure 2:**
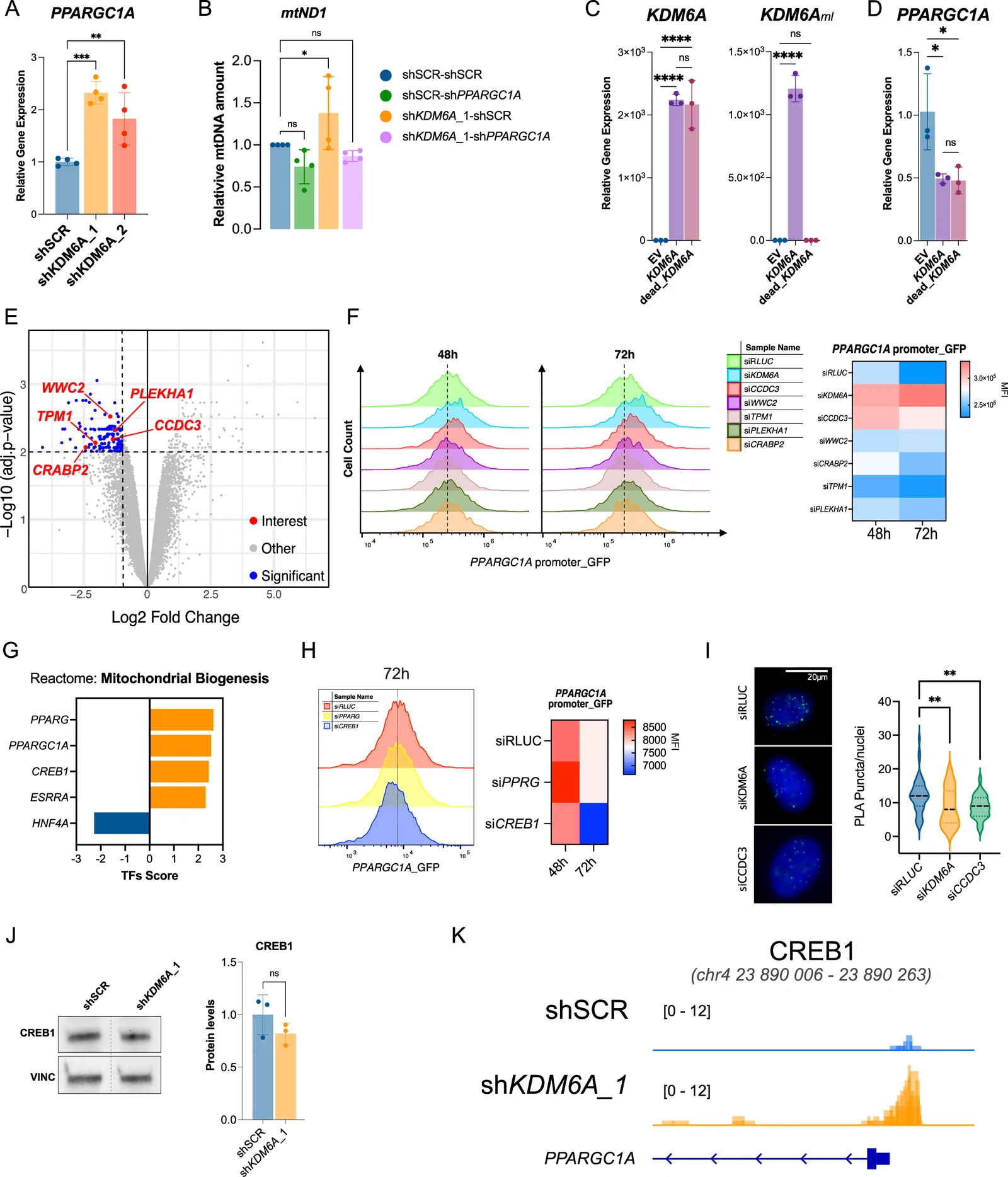
KDM6A loss dowregulates *CCDC3* eliciting *PPARGC1A* expression induced by CREB1 in solid tumors. (**A**) Gene expression levels of *PPARGC1A*, assessed by RT-qPCR on U-2 OS cell line silenced with two different shRNAs targeting *KDM6A*. Data are normalized to *TBP* and to shSCR condition and represented as mean (+/− SD) of four independent biological replicates. One-way ANOVA test was performed (***p = 0.0002, **p = 0.0053). (**B**) Abundance of mtDNA (*mtND1)* measured by qPCR in controls or silenced U-2 OS cells using shSCR, sh*KDM6A*, sh*PPARGC1A, alone or combined*. Data are normalized to *NBPF9* and to shSCR-shSCR condition and represented as mean +/− SD of four independent biological replicates. One-way ANOVA test was performed (ns *p* = 0.1547, *p = 0.0459, ns *p* = 0.448). (**C**) Gene expression of *KDM6A* on the WT and specifically on the mutation *locus* (KDM6Aml) and *PPARGC1A* (**D**), assessed by RT-qPCR on U-2 OS cells. Data are normalized to *TBP* and to Empty Vector (EV) control and represented as mean +/− SD of three independent biological replicates. One-way ANOVA test was performed (*KDM6A*: ****p = 0.000019, ****p = 0.000023, ns *p* = 0.70729; *KDM6Aml*: ****p = 0.0000006, ns *p* = 0.9999974; *PPARGC1A*: *p = 0.013, *p = 0.0116, ns *p* = 0.9292). (**E**) Volcano Plot showing differentially expressed genes (DEGs) in sh*KDM6A* vs shSCR conditions. Top 5 genes ranked by KDM6A Binding Score are highlighted in red. Significantly downregulated genes are represented in blue (*n* = 3, limma, adj. *p*-value < 0.01). (**F**) *PPARGC1A* promoter-driven GFP fluorescence was assessed by flow cytometry (FACS) at 48 h and 72 h after silencing the expression of *KDM6A*, *CCDC3*, *WWC2*, *TPM1*, *PLEKHA1* or *CRABP2*, respectively. As non-targeting control, siRLUC was used. On the right, an heatmap showing the Median Fluorescence Intensity (MFI) of GFP in U-2 OS cells at 48 h and 72 h after silencing of the genes. (**G**) Transcription factors (TFs) score calculated from sh*KDM6A* *vs* shSCR RNAseq. TFs derive from Reactome: Mitochondrial Biogenesis database. (**H**) *PPARGC1A* promoter-driven GFP fluorescence measured by FACS at 48 h and 72 h after silencing the expression of *PPARG* and *CREB1*. As non-targeting control, siRLUC was used. Heatmap showing MFI. (**I**) Representative images and quantification of Proximity Ligation Assay (PLA) foci quantified as PLA puncta/nuclei in U-2 OS siRLUC, si*KDM6A*, and si*CCDC3* conditions (Scale bar: 20 μm). Significant differences among groups were quantified by applying two-sided Student’s t-test (*n*: siLUC = 47, si*KDM6A*  =  34, si*CCDC3*  =  29; **p = 0.0073, **p = 0.0095). (**J**) One representative western blot of CREB1 upon shSCR and sh*KDM6A*. Three independent biological replicates, two-tailed unpaired Student’s t-test (ns *p* = 0.2184). (**K**) CUT&TAG peak of CREB1 on the *PPARGC1A* promoter region in shSCR and sh*KDM6A* conditions from three independent biological replicates (overlay of three peaks). Source data are available online for this figure.

**Figure EV2:**
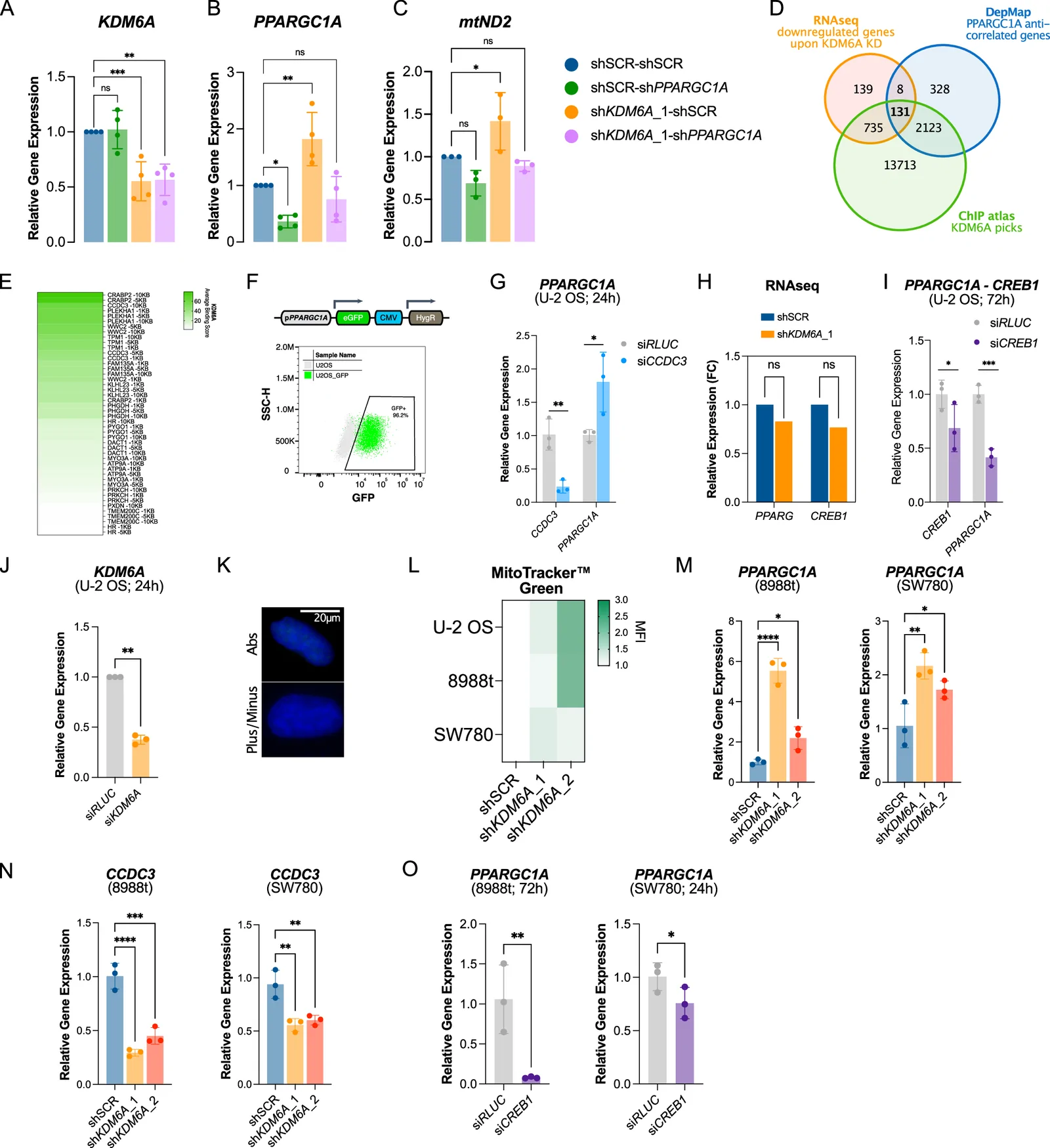
CCDC3 loss induces *PPARGC1A* expression mirroring the effect of *KDM6A* KD in PaTu8988t and SW780 as observed in U-2 OS cells. (**A**) Gene expression levels of *KDM6A* and (**B**) *PPARGC1A* in U-2 OS with double shRNAs assessed by RT-qPCR. Data are normalized to *TBP* and to shSCR-shSCR condition and represented as mean +/− SD of four independent biological replicates. Statistical significance was assessed by applying one-way ANOVA test (*KDM6A*: ns *p* = 0.8367, ***p = 0.0008, **p = 0.001; *PPARGC1A*: *p = 0.0141, **p = 0.0031, ns *p* = 0.2954). (**C**) mtDNA quantification (*mtND2*) was measured through qPCR. Data are normalized to *NBPF9* and to shSCR-shSCR condition and represented as mean +/− SD of three independent biological replicates. Statistical significance was assessed by applying one-way ANOVA test (ns *p* = 0.0761, *p = 0.0259, ns = 0.4932). (**D**) Venn diagram of shared significant genes between  RNAseq, DepMap, and ChIPAtlas. (**E**) Genes ranked by *KDM6A* average binding score. (**F**) GFP-positive cells after transduction with *PPARGC1A* expression reporter. (**G**) Gene expression levels measured by RT-qPCR of *PPARGC1A* and *CCDC3* in U-2 OS after 24 h silencing with siRNA. Data are normalized to *TBP* and to siRLUC control and represented as mean +/− SD of three independent biological replicates. Statistical significance was assessed by applying multiple unpaired t-test (**p = 0.0058, *p = 0.0386). (**H**) Gene expression of *PPARG* and *CREB1* from RNAseq (*n* = 3, limma, ns adj. *p* = 0.51, ns adj. *p* = 0.14). (**I**) Gene expression levels analyzed by RT-qPCR of *CREB1* and *PPARGC1A* in U-2 OS after 72 h silencing with siRNA. Data are normalized to *TBP* and to siRLUC control and represented as mean +/− SD of three independent biological replicates. Statistical significance was assessed by applying one-tailed unpaired t-test (*p = 0.05, ***p = 0.0004). (**J**) Expression levels of *KDM6A* measured by RT-qPCR in U-2 OS after 24 h silencing with siRNA. Mean +/− SD, *n* = 3. Statistical significance was assessed by applying one sample t-test (**p = 0.0017). (**K**) PLA negative controls. Abs = only antibodies. Plus/Minus = only probes. (**L**) Heatmap showing the median fluorescence intensity (MFI) of MitoTracker™ Green in U-2 OS, PaTu8988t, and SW780 cell lines. (**M**) Expression levels assessed by RT-qPCR of *PPARGC1A* and (**N**) *CCDC3*, on PaTu8988t and SW780 cell lines silenced with two different shRNAs targeting *KDM6A*. Data are normalized to *TBP* and to shSCR condition and represented as mean +/− SD of three independent biological replicates. One-way ANOVA test was performed (*PPARGC1A*: (8988t) ****p = 0.000028, *p = 0.024629; (SW780) **p = 0.0033, *p = 0.0304. *CCDC3*: (8988t) ****p = 0.000049, ***p = 0.000198; (SW780) **p = 0.0018, **p = 0.0034). (**O**) Gene expression levels measured by RT-qPCR of *CREB1* in PaTu8988t and SW780 cell line after *KDM6A* silencing. Data are normalized to *TBP* and to control condition (siRLUC) and represented as mean +/− SD of three independent biological replicates. Data were collected at 72 h and 24 h, respectively. Statistical significance was assessed by one-tailed Unpaired t-test (8988t: **p = 0.0083; SW780 *p = 0.0476).

PPARG Coactivator 1 Alpha (*PPARGC1A/PGC1α*) drives mitochondria biogenesis (Puigserver et al, 1998). We explored whether the increase in mitochondrial mass elicited by *KDM6A* KD could be linked to the modulation of *PPARGC1A* expression. We indeed detected a robust increase on *PPARGC1A* expression upon *KDM6A* loss (Fig. 2A). Notably, knocking down *PPARGC1A* alongside *KDM6A* (Fig. EV2A,B) prevented the increase in mitochondria biogenesis elicited by *KDM6A* KD (Figs. 2B and EV2C), suggesting a role for PPARGC1A in mediating the increase in mitochondrial mass induced by *KDM6A* loss.

Interestingly, KDM6A activity is often independent from its catalytic activity (Gozdecka et al, 2018; Pacella et al, 2025). In order to assess the relevance of the catalytic activity of KDM6A in U-2 OS we transfected a catalytically mutant KDM6A (Wang et al, 2012), or a wild-type (WT) isoform (Fig. 2C), and assessed *PPARGC1A* expression. Overexpression of WT *KDM6A* downregulated *PPARGC1A* expression, as anticipated. However, also the expression of the catalytically dead KDM6A resulted in a similar *PPARGC1A* downregulation (Fig. 2D), suggesting that the effect of KDM6A on *PPARGC1A* is not explained by its catalytic function, but more likely by its scaffolding activity.

Since KDM6A is generally a gene activator, and KDM6A loss led to increased *PPARGC1A* transcription, we anticipated that a transcriptional repressor, directly regulated by KDM6A, may modulate *PPARGC1A* expression levels. To identify a KDM6A target able to inhibit *PPARGC1A* expression, we integrated our own RNA-seq data from U-2 OS cells depleted for KDM6A with information obtained from the DepMap portal (Tsherniak et al, 2017), as well as from the ChIP Atlas website (Oki et al, 2018) (Fig. EV2D and see Methods). Specifically, we selected the genes that were downregulated in our dataset upon *KDM6A* KD, that were inversely correlated with *PPARGC1A* expression in DepMap, and that were bound at their promoters or regulatory regions by KDM6A in the ChIP Atlas website (Fig. 2E). Among the emerging genes, we focused on *CRABP2*, *CCDC3*, *PLEKHA1*, *WWC2* and *TPM1*, the top 5 genes that were ranked by KDM6A average binding score (MACS2 score from Peak-call data) (Fig. EV2E).

We then asked whether the downregulation of any of these genes could elicit *PPARGC1A* expression, similar to the result obtained with *KDM6A* KD. To this end, we engineered a U-2 OS cell line harboring a reporter cells system including GFP under the control of the *PPARGC1A* promoter (Fig. EV2F). *KDM6A* KD increased the expression of GFP in these cells. We then downregulated the expression of these 5 genes using specific small interfering RNAs (siRNAs). We found that the KD of one gene, coding for the coiled-coil domain containing 3 (CCDC3) protein increased GFP at levels comparable to si*KDM6A* (Fig. 2F) as well as the RNA level of *PPARGC1A* (Fig. EV2G). CCDC3 is a small, secreted protein with a coiled-coil domain, which mediates protein-protein interactions. The role of CCDC3 remains largely unknown, besides its regulation of the transcription of adipogenic and lipogenic genes, through an unknown mechanism (Kobayashi et al, 2015). CCDC3 also impairs the MDM2-mediated recruitment of p53 to the 26S proteosome (Li et al, 2023). We reasoned that CCDC3 might interfere with the activity of a transcription factor inducing the expression of *PPARGC1A*. We then sought to identify the transcription factors implicated in mitochondria biogenesis whose activity was enhanced upon *KDM6A* KD in our RNA-seq data. Besides *PPARGC1A* itself, and the Estrogen Related Receptor Alpha (ESRRA), a downstream target of PPARGC1A (Schreiber et al, 2003), we identified two other transcription factors, PPARG and CREB1 (Fig. 2G). Previous studies demonstrated how PPARG coactivates (Puigserver et al, 1998), and CREB1 induces *PPARGC1A* (Herzig et al, 2001). Neither of these transcription factors were upregulated upon *KDM6A* KD (Fig. EV2H); thus, we speculate that the loss of a putative binding with CCDC3 could increase their activity. To ascertain which of the two TFs was modulated by CCDC3, we exploited the engineered cell lines described above and tested whether the downregulation of any of these transcription factors by siRNAs was able to elicit *PPARGC1A* expression. si*CREB1* strongly reduced the GFP signal and *PPARGC1A* RNA level (Figs. 2H and EV2I) supporting the notion that CREB1 regulates the transcription of *PPARGC1A* also in our system. We next explored whether CCDC3 and CREB1 interacted. A proximity ligation assay (PLA) revealed a co-localization signal for CCDC3 and CREB1 mostly in the nucleus, suggesting that the two proteins interacted. This interaction was prevented by *KDM6A* (Fig. EV2J) as well as *CCDC3* siRNAs (Figs. 2I and EV2K). Notably, CREB1 protein levels were not modified upon *KDM6A* KD (Fig. 2J), suggesting that the loss of KDM6A does not impair CREB1 stability. We hence explored whether *KDM6A* KD might impact on CREB1 localization on the *PPARGC1A* locus. Indeed, upon KDM6A loss, there was an enrichment of CREB1 on the promoter region of the *PPARGC1A* gene (Fig. 2K).

In all, we conclude that *KDM6A* silencing prevents *CCDC3* transcription, unleashing CREB1 transcriptional activity throughout its recruitment on *PPARGC1A* locus.

We next expanded our observations to cancer types where KDM6A is frequently inactivated, namely urothelial bladder carcinoma and pancreatic cancer. *KDM6A* KD in the pancreatic cancer cell line PaTu 8988t and bladder carcinoma cell line SW780 resulted in increased mitochondrial mass (Fig. EV2L) and *PPARGC1A* expression (Fig. EV2M), confirming results obtained in U-2 OS cells. Accordingly, *CCDC3* expression levels were reduced upon *KDM6A* KD (Fig. EV2N) in both cell lines and *CREB1* KD also led to a reduction in *PPARGC1A* levels (Fig. EV2O) in both cell lines.

### *KDM6A* KO elicits OXPHOS in multiple myeloma patient samples

**Figure 3:**
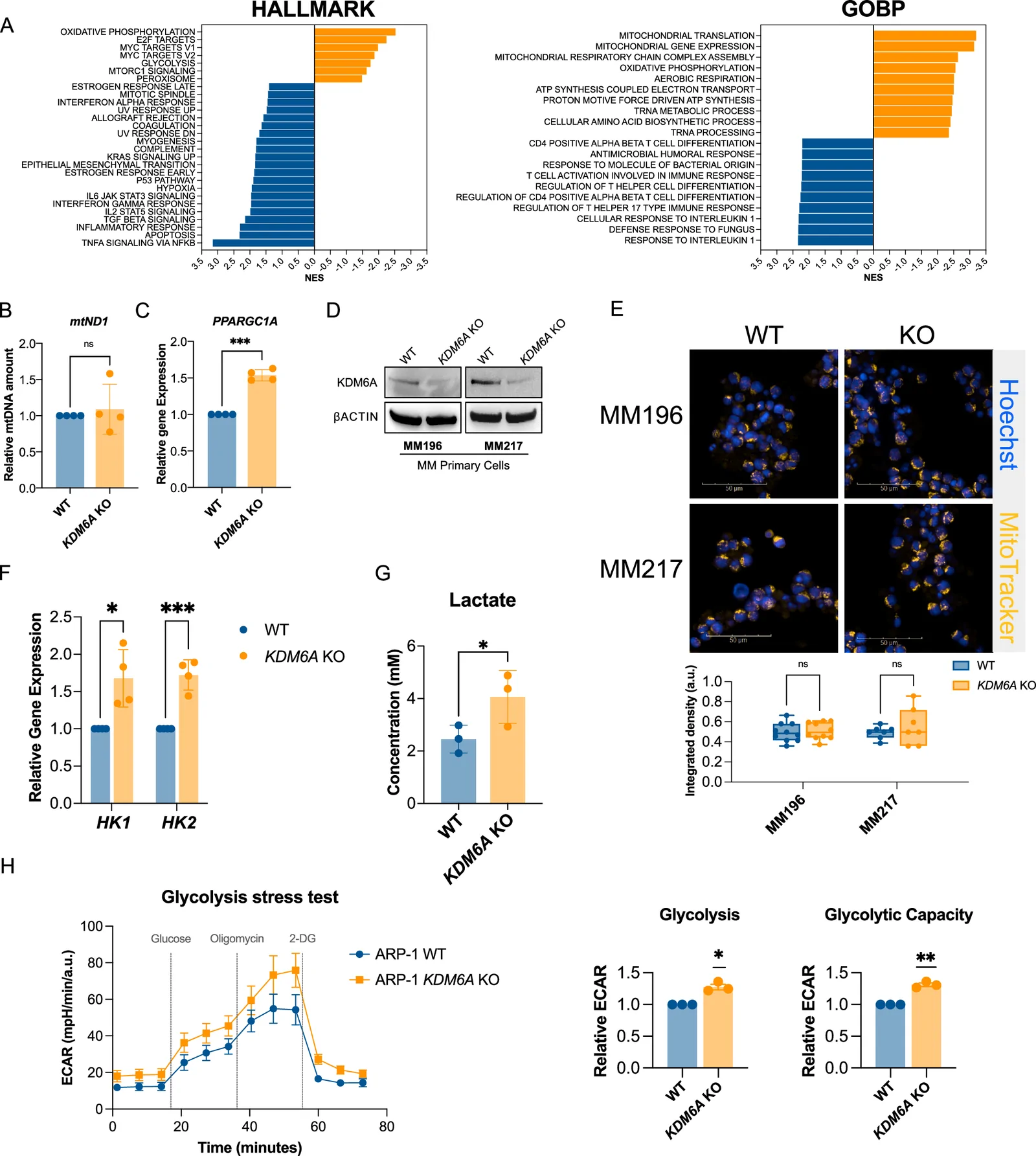
Glycolysis induction predominates in *KDM6A* KO MM cell lines. (**A**) GSEA (v4.3.2) analysis of transcriptome data from MMRF CoMMpass™ study patients’ cohort. Negative NES represent pathways anti-correlated with *KDM6A* expression in MM patients. Pathways derived from HALLMARK and Gene Ontology Biological Processes (GOBP). (**B**) Abundance of mtDNA (*mtND1*) in WT and *KDM6A* KO ARP-1 cell line measured by qPCR. Data are normalized to *NBPF9* and to WT condition and represented as mean +/− SD of four independent biological replicates. Significant differences were quantified by one sample t-test (ns *p* = 0.6262). (**C**) RNA levels of *PPARGC1A* analyzed by RT-qPCR in WT and *KDM6A* KO ARP-1 cell line. Data are normalized to *TBP* and to WT and represented as mean +/− SD of four independent biological replicates. Significant differences were quantified by one sample t-test (***p = 0.000008). (**D**) Western blot of KDM6A protein level in primary MM cell lines at 48 h after *KDM6A* KO by CRISPR-Cas9. (**E**) Measurement of the median integrated density of Mitotracker Red signal. Images acquired using the Opera confocal microscope, 7 to 10 different acquisitions coming from three independent biological replicates (multiple unpaired t test, ns *p* = 0.712442, ns *p* = 0.420328). MM196 WT: *n* = 10, Min = 0.36, Q1 = 0.415, Median = 0.484, Q3 = 0.581, Max = 0.664; MM196 KO: *n* = 10, Min = 0.374, Q1 = 0.443, Median = 0.495, Q3 = 0.599, Max = 0.609; MM217 WT *n* = 7, Min = 0.387, Q1 = 0.443, Median = 0.498, Q3 = 0.526, Max = 0.581; MM217 KO: *n* = 7, Min = 0.36, Q1 = 0.36, Median = 0.498, Q3 = 0.719, Max = 0.858. (**F**) *Hexokinases* (*HK1 and 2*) RNA levels were measured by RT-qPCR in WT and *KDM6A* KO ARP-1 cell line. Data are represented as mean +/− SD of four independent biological replicates and are normalized on the WT condition. Significant differences were quantified by applying multiple unpaired t tests (*p = 0.0124, ***p = 0.0004). (**G**) Lactate concentration was measured in ARP-1 medium after 96 h of culture. Data are represented as mean +/− SD of three independent biological replicates. Significant differences were quantified by one-tailed Paired Student’s t-test (*p = 0.0305). (**H**) Representative analysis of ECAR measurements and bar plots of glycolysis and glycolytic capacity in ARP-1 cells. Mean +/− SEM. Significant differences of three independent biological replicates were quantified by one sample t-test (*p = 0.0234, **p = 0.0079). Source data are available online for this figure.

**Figure EV3:**
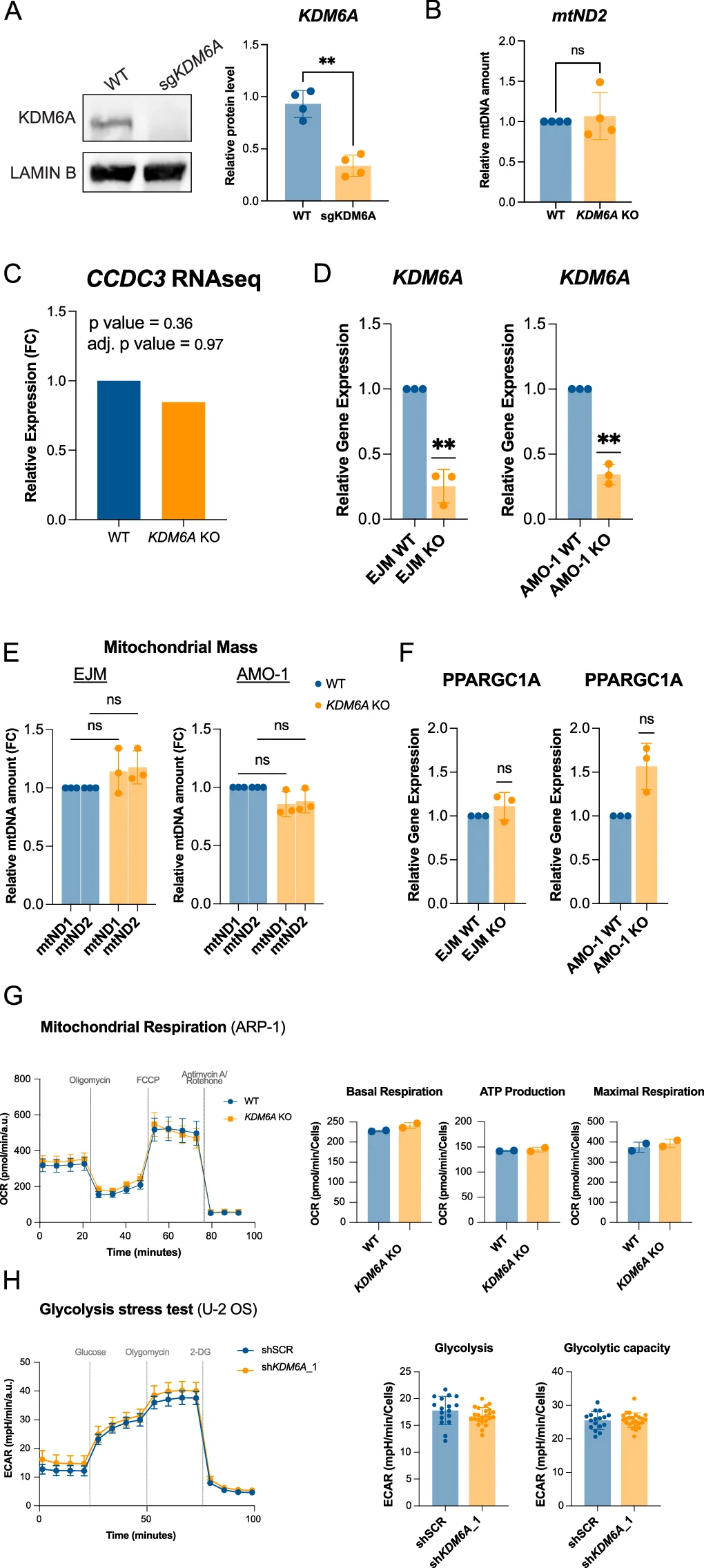
MM cell lines do not exhibit increased mitochondrial mass, *PPARGC1A* expression or OXPHOS upon *KDM6A* KO. (**A**) One representative out of four western blot and quantification of KDM6A after CRISPR Cas9 KO in ARP-1 cells. Protein levels of KDM6A were measured in four independent biological replicates and normalized to LAMIN B. Mean +/− SD. Two-tailed Paired Student’s t-test (**p = 0.0049). (**B**) Abundance of mtDNA (*mtND2*) measured by qPCR in WT and KO ARP-1 cell line. Data are normalized to *NBPF9* and to WT condition and represented as mean +/− SD of four independent biological replicates. Statistical significance was assessed by one sample t-test (ns *p* = 0.6783). (**C**) *CCDC3* expression from RNAseq in WT and *KDM6A* KO ARP-1 cells (*n* = 4, limma). (**D**) Gene expression of *KDM6A* in WT and KO EJM and AMO-1 cell lines analyzed by RT-qPCR. Data are normalized to *TBP* and to WT and represented as mean +/− SD of three independent biological replicates. Statistical significance was assessed by one sample t-test (**p = 0.0097, **p = 0.0045). (**E**) Abundance of mtDNA (*mtND1* and *mtND2*) in WT and KO EJM and AMO-1 cell lines measured by qPCR. Data are normalized to *NBPF9* and to WT condition and represented as mean +/− SD of three independent biological replicates. Statistical significance was assessed by multiple unpaired t-test (EJM: ns *p* = 0.276, ns *p* = 0.0978; AMO-1: ns *p* = 0.083, ns *p* = 0.1046). (**F**) Expression of *PPARGC1A* in WT and *KDM6A* KO EJM and AMO-1 cell lines analyzed by RT-qPCR. Data are normalized to *TBP* and to WT and represented as mean +/− SD of three independent biological replicates. Statistical significance was assessed by one sample t-test (ns *p* = 0.3467, ns *p* = 0.0648). (**G**) One representative analysis out of two independent biological replicates of OCR measurements. Bar plots of basal respiration, ATP production, and Maximal Respiration of two independent biological replicates. Mean +/− SD. (**H**) Analysis of ECAR measurements and bar plots of glycolysis and glycolytic capacity on U-2 OS cell line. Mean +/− SD (*n* = 17, *n* = 24; *n* = 17, *n* = 24).

We next explored the role of KDM6A loss in the blood cancer multiple myeloma. We started analyzing the transcriptomic data obtained in patients enrolled into the CoMMpass^SM^ dataset from the Multiple Myeloma Research Foundation (MMRF^®^) (Skerget et al, 2024). Also in this cancer type, the OXPHOS pathway was the most prominent pathway activated (Fig. 3A). However, to our surprise, when we assayed the impact of *KDM6A* KO in the ARP-1 MM cell line (Fig. EV3A), we did not detect any increase in mitochondrial mass (Figs. 3B and EV3B) and only a modest or not significative increase in *PPARGC1A* RNA levels (Fig. 3C). These data were confirmed in two additional MM cell lines (EJM and AMO-1) knocked out for *KDM6A* (Fig. EV3D–F). Additionally, we knocked out *KDM6A* in two MM primary samples (Bruno et al, 2020) (Fig. 3D), where accordingly mitochondrial mass (Fig. 3E) was also unchanged.

Furthermore, in ARP-1 cell line, *KDM6A* KO did not reduce *CCDC3* expression (Fig. EV3C). Accordingly, the oxygen consumption rate (OCR) measured by Seahorse (Fig. EV3G) did not increase upon *KDM6A* KO. In all, these results suggest that in MM cells, *KDM6A* downregulation per se does not lead to an increase in OXPHOS. Conversely, we detected an increase in the expression of *Hexokinase1 and 2* (*HK1/2*), enzymes implicated in the early glycolytic pathway, upon *KDM6A* KO (Fig. 3F), alongside lactate, a byproduct of the pyruvate reduction at the end of the glycolytic pathway (Fig. 3G). Finally, Seahorse analysis, assaying extracellular acidification rate (ECAR), confirmed that both glycolysis and glycolytic capacity were increased upon *KDM6A* ablation (Fig. 3H) in MM cells. By contrast, ECAR was not increased by *KDM6A* KD in U-2 OS cells (Fig. EV3H). In all, these results strongly suggest that in MM *KDM6A* depletion increases glycolysis, an entirely different phenotype from what observed in solid tumor cell lines.

### KDM6A loss drives mitochondrial transfer from cocultured bone marrow stromal cells

**Figure 4:**
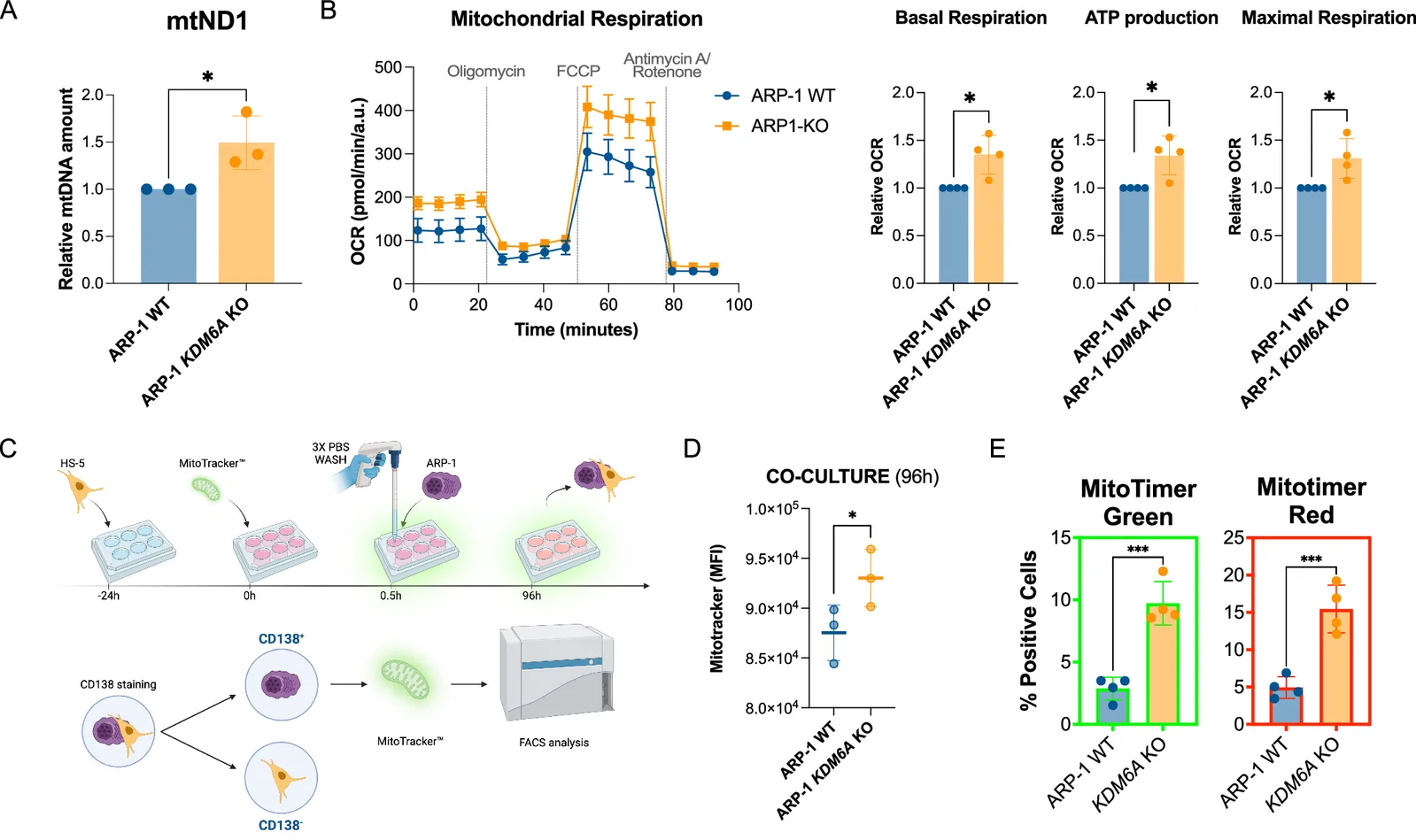
Mitochondria originating from bone marrow stromal cells fuel OXPHOS in MM cells KO for *KDM6A*. (**A**) mtDNA measured through qPCR in ARP-1 cells following co-culture with HS-5. Data are normalized to *NBPF9* and to WT condition and represented as mean +/− SD of three independent biological replicates. Significant differences were quantified by one-tailed one sample t-test (*p = 0.048). (**B**) One representative analysis out of four independent biological replicates of OCR measurements in ARP-1 cells following co-culture with HS-5. Bar plots of basal respiration, ATP production, and Maximal Respiration from four biological replicates (mean, +/− SEM). Significant differences were quantified by one-tailed one sample t-test (*p = 0.021, *p = 0.022, *p = 0.03). (**C**) Schematic representation of plating and coculture of HS-5 and ARP-1 cell lines with MitoTracker^TM^ staining and FACS analysis. (**D**) Measurement of MitoTracker^TM^ green Median Fluorescence Intensity (MFI) in CD138+ cells after 96 h coculture. Mean +/− SD, *n* = 3. One-tailed paired t test (*p = 0.0256). (**E**) Percentage of HS-5 cocultured ARP-1 cells positive to Mitotimer green and red. Mean +/− SD (*n* = 4). Unpaired t test (***p = 0.0004, ***p = 0.001). Source data are available online for this figure.

**Figure EV4:**
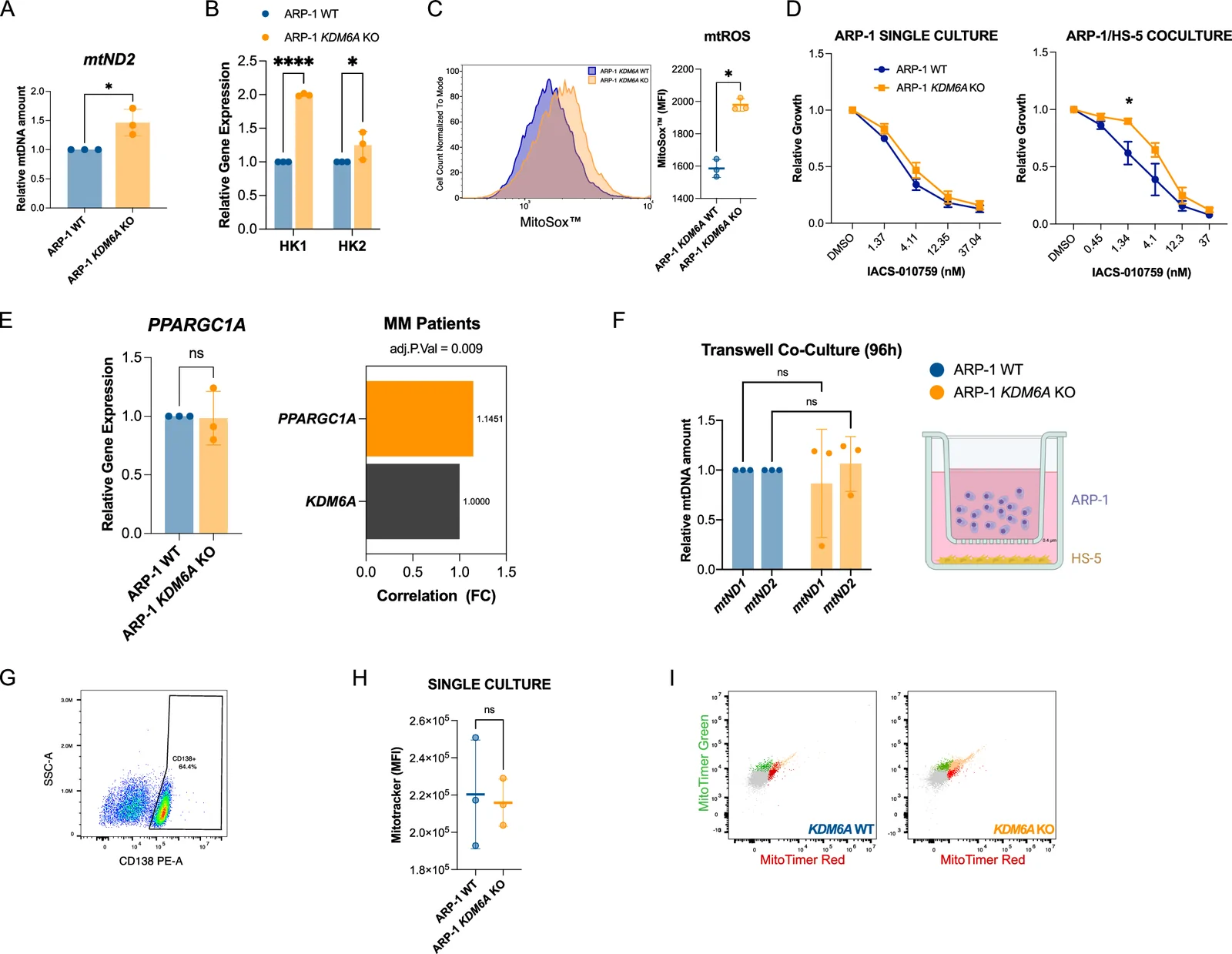
KDM6A KO in MM cells increases glycolysis, ROS production and resistance to OXPHOS inhibitors upon coculture as a result of cell-to-cell contact. (**A**) Abundance of mtDNA (*mtND2*) measured by qPCR after coculture. Data are normalized to *NBPF9* and to WT condition and represented as mean +/− SD of three independent biological replicates. Statistical significance was assessed by one sample t-test (*p = 0.036). (**B**) Gene expression of *HK1* and *HK2* in ARP-1 cells following co-culture with HS-5. RT-qPCR data are normalized to *TBP* and to WT and represented as mean +/− SD of three independent biological replicates. Statistical significance was assessed by two-way ANOVA (****p = 0.00002, *p = 0.019342). (**C**) One representative histogram and quantification of MitoSox™ staining of ARP-1 cells following co-culture with HS-5. MFI measurements from three independent biological replicates. Mean +/− SD. Statistical significance was assessed by applying paired Student’s t-test (*p = 0.0114). (**D**) ARP-1 single culture and coculture treated with IACS-010759 at different concentrations. Mean +/− SEM (*n* = 3). Unpaired t test (*p = 0.0265). (**E**) On the left, gene expression measured by RT-qPCR of *PPARGC1A* in ARP-1 cells following co-culture with HS-5. Data are normalized to *TBP* and to WT and represented as mean +/− SD of three independent biological replicates. Statistical significance was assessed by one sample t-test (ns *p* = 0.9128). On the right, linear regression of *PPARGC1A* and *KDM6A* in MM patients. Data shown as fold change of the correlation. (**F**) Abundance of mtDNA (*mtND1*, *mtND2*) analyzed by qPCR in ARP1 cells when cocultured with HS-5 in a transwell system. Data are normalized to *NBPF9* and to WT condition and represented as mean +/− SD of three independent biological replicates. Statistical significance was assessed by two-tailed Unpaired Student’s t-test (ns *p* = 0.691167, ns *p* = 0.717478). Schematic representation of transwell (0.4 µm) coculture. (**G**) Gated CD138 positive cells after ARP-1/HS-5 coculture. (**H**) MFI of MitoTracker™ green stained ARP-1 *KDM6A* WT and KO cell line in single culture. Mean +/− SD, *n* = 3. One-tailed paired t test (ns *p* = 0.343). (**I**) Dot plot of Mitotimer green and red positive *KDM6A* WT and KO ARP-1 cells after HS-5 coculture.

To explain this prominent discrepancy between MM cell lines and patient samples, we reasoned that the main difference between the two conditions ought to do with the presence of a tumor microenvironment in the patients’ bone marrow where plasma cells reside. To test this hypothesis, we cocultured ARP-1 with an established microenvironment model system, the bone marrow stromal cell line (HS-5). Intriguingly, upon co-culture with stromal cells, mtDNA increased in *KDM6A* KO cells compared to *KDM6A* WT cells (Figs. 4A and EV4A). Of note, also glycolysis remained active after co-culture, as indicated by the overexpression of hexokinases (HKs) (Fig. EV4B). Notably, Seahorse analysis demonstrated that the increase in mitochondria was associated with increased OXPHOS (Fig. 4B). In accordance with these findings, mitochondrial ROS was also increased in these cells, assayed with MitoSox™ (Fig. EV4C). To further substantiate the critical role exerted by OXPHOS activation in MM cells grown in the presence of stromal cells, we treated MM cells with the OXPHOS inhibitor IACS-010759 (Saito et al, 2021). In the absence of stromal cells, WT and *KDM6A* KO cells were equally sensitive to this treatment. By contrast, when stromal cells were added, *KDM6A* KO ARP-1 cells were more resistant to IACS-010759 than WT cells (Fig. EV4D). Of note, *PPARGC1A* was not upregulated in MM cell lines upon *KDM6A* KD, nor the two genes were inversely correlated in MM patients (Fig. EV4E), suggesting that there was no new mitochondria biogenesis.

We reasoned that the increase in mitochondrial mass and OXPHOS in MM cells, detected in patients and present in co-cultured MM cell lines, might depend on a soluble factor emanating from the stromal cells, signaling to the MM cells. However, when MM and stromal cells were grown in a trans-well system, mtDNA in *KDM6A* KO MM cells did not increase (Fig. EV4F), suggesting that direct interactions between MM and HS-5 cells are required to increase MM OXPHOS.

We then hypothesized that the increased OXPHOS in *KDM6A* KO MM cells could be due to cell-to-cell transfer of mitochondria from the stromal to the MM cells. To test this notion, HS-5 cells were stained with Mitotracker™ Green, grown with ARP-1 MM cells (Fig. 4C) and analyzed by flow cytometry. After coculture and the MM surface marker CD138^+^ gating (Fig. EV4G), more fluorescent mitochondria were detected in *KDM6A* KO compared with WT MM cells (Fig. 4D). By contrast, simply staining *KDM6A* KO and WT cells with Mitotracker™ cultured without stromal cells revealed no differences in mitochondria mass (Fig. EV4H). To confirm these data, we transfected HS-5 cells with a plasmid encoding MitoTimer, a fluorescent reporter protein which tracks the mitochondrial maturation (Hernandez et al, 2013) and measured the mitochondrial content in ARP-1 cells. Both newly expressed (green) and mature (red) MitoTimer stained mitochondria were significantly increased in *KDM6A* KO ARP-1 cells compared to WT counterparts (Figs. 4E and EV4I), confirming that a direct transfer of mitochondria from stromal cells to MM cells is driven by KDM6A loss.

### *KDM6A* KO MM cells induce TNTs connection with bone marrow stromal cells

**Figure EV5:**
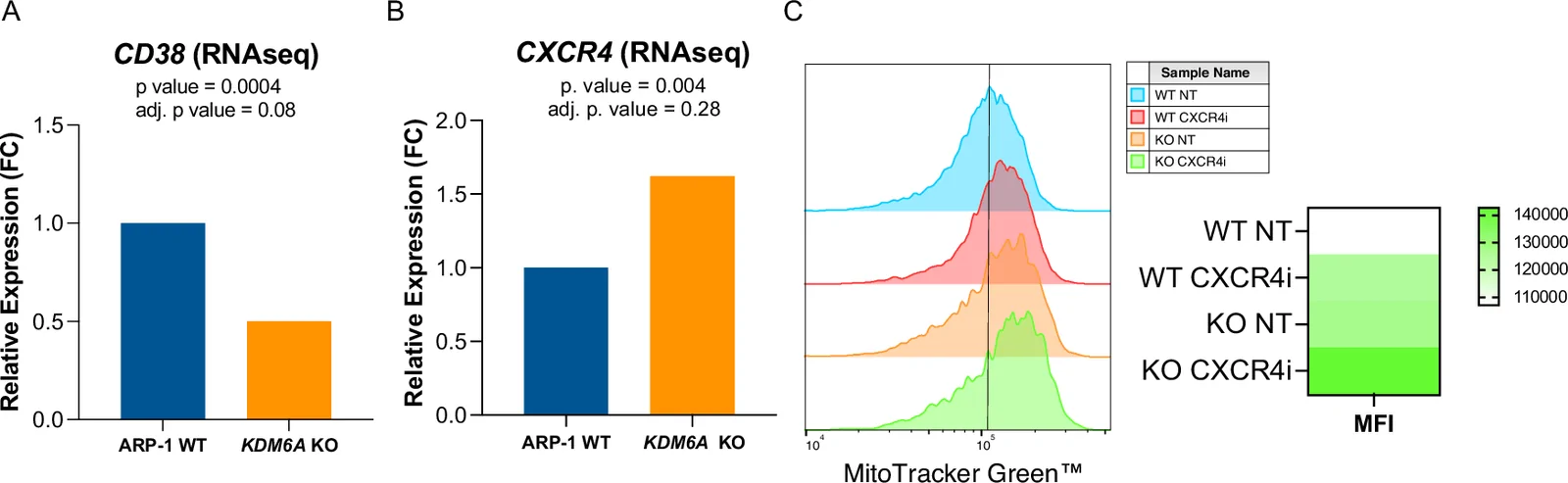
Mitochondrial trafficking regulators *CD38* and *CXCR4* are not induced by KDM6A loss. (**A**) Expression of *CD38* from RNAseq in ARP-1 cells. (**B**) *CXCR4* expression from RNAseq in WT and *KDM6A* KO ARP-1 cells. (**C**) MitoTracker™ green fluorescence measured by FACS in CXCR4i treated HS-5 cocultured ARP-1 cells.

**Figure 5:**
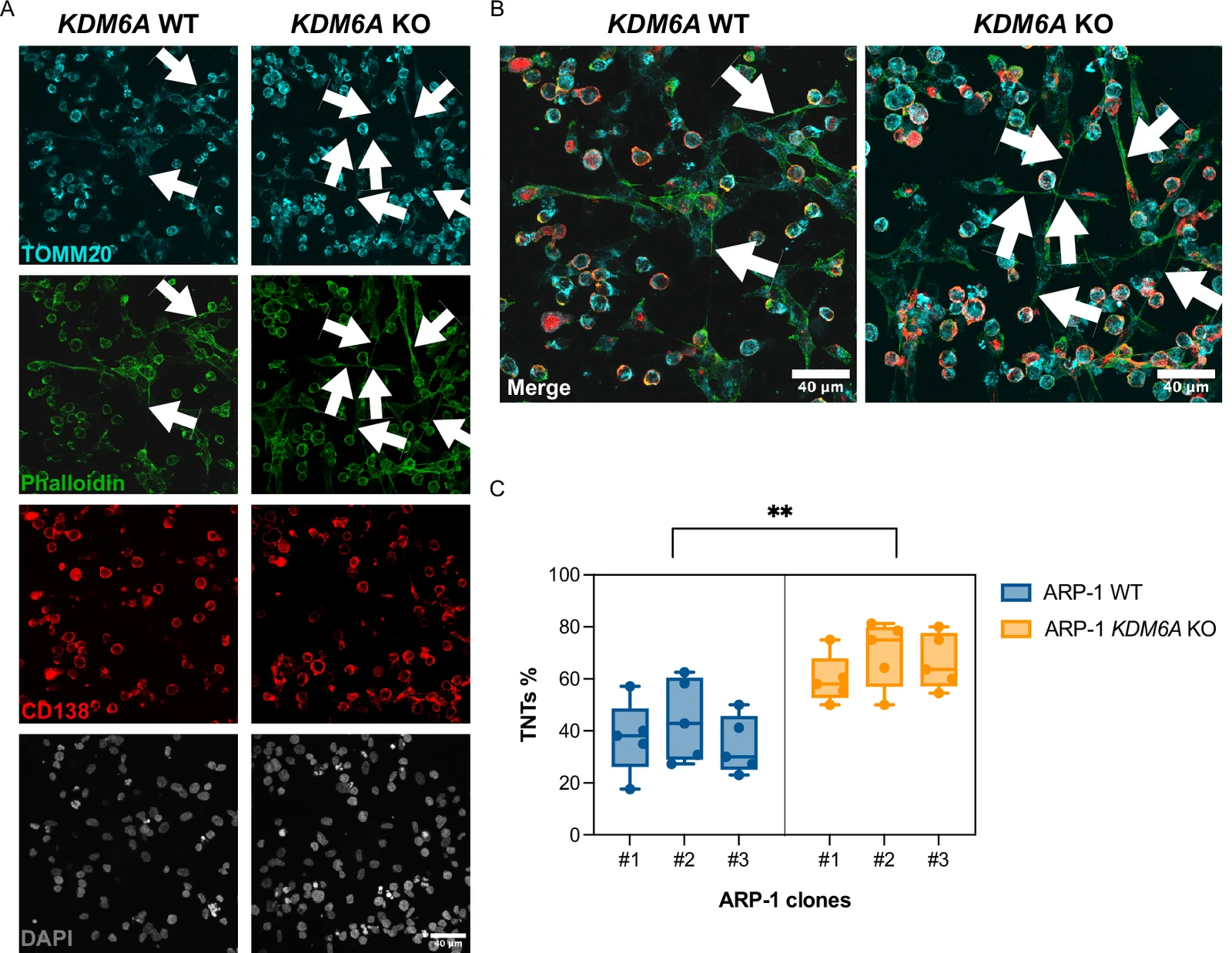
TNTs increase upon *KDM6A* KO in MM cells. (**A**) One representative image of TNTs in ARP-1 and HS-5 coculture. White arrows indicate TNTs. Anti-TOMM20 antibody was used for mitochondria staining, CD138 characterizes ARP-1 cell line, phalloidin stains F-actin, and DAPI the nucleus (Scale bar: 40 μm). (**B**) Merged images of TOMM20, CD138, and phalloidin. Arrows indicate TNTs between ARP-1 and HS-5 (Scale bar: 40 μm). (**C**) TNTs were quantified from three independent biological replicates. Significant differences were quantified by two-tailed Nested Student’s t-test (**p = 0.0041). WT#1: *n* = 5, Min = 17.65, Q1 = 26.32, Median = 38.10, Q3 = 48.57, Max = 57.14; WT#2: *n* = 5, Min = 27.27, Q1 = 29.02, Median = 42.86, Q3 = 60.42, Max = 62.50; WT#3: *n* = 5, Min = 23.08, Q1 = 25.17, Median = 30, Q3 = 45.59, Max = 50; KO#1: *n* = 5, Min = 50, Q1 = 52.78, Median = 58.06, Q3 = 67.76, Max = 75; KO#2: *n* = 5, Min = 50, Q1 = 57.14, Median = 75, Q3 = 79.91, Max = 81.25; KO#3: *n* = 5, Min = 54.55, Q1 = 57.27, Median = 63.64, Q3 = 77.5, Max = 80. Source data are available online for this figure.

We then tested receptors implicated in mitochondrial trafficking, as for example the MM-specific surface protein CD38 (Marlein et al, 2019). Our RNAseq data, however, showed a reduction of CD38 upon *KDM6A* KO (Fig. EV5A). The chemokine receptor CXCR4 after its binding to CXCL12 enhances mitochondrial uptake through gap junctions (Giallongo et al, 2022). From RNAseq CXCR4 was not significantly deregulated upon *KDM6A* KO (Fig. EV5B), nor the CXCR4 inhibitor AMD3100 impacted on the transfer of mitochondria from stroma to MM cells as MitoTracker™ staining of the cocultured MM cells remained unchanged (Fig. EV5C). Tunneling nanotubes (TNTs) represent another mechanism of mitochondrial trafficking, whereby these membranous structures are established between bone marrow stromal cells and MM cells (Boise and Shanmugam, 2019; Iorio et al, 2024; Lin et al, 2024). We assessed the presence of TNTs in cocultured ARP-1 and HS-5 by immunofluorescence, staining with an antibody against TOMM20 for mitochondria detection and phalloidin bound to F-actin for TNTs (MM ARP-1 cells were detected with CD138) (Fig. 5A,B). *KDM6A* KO cells established a significantly higher number of connections with HS-5 cells through TNTs than WT cells (Fig. 5C).

We conclude that KDM6A is pivotal in regulating mitochondrial flux from stromal to MM cells via the establishment of a thick net of TNTs between cancer cells and the surrounding environment.

### TRAF3IP3 is a regulator of mTORC1 activation in *KDM6A* KO MM cells

mTORC1 belongs to the few pathways able to control both glycolysis (Pollizzi et al, 2016) and mitochondrial transport (Wang et al, 2011). We hence assessed whether the mTORC1 pathway was activated upon *KDM6A* ablation in MM cells.

**Figure 6:**
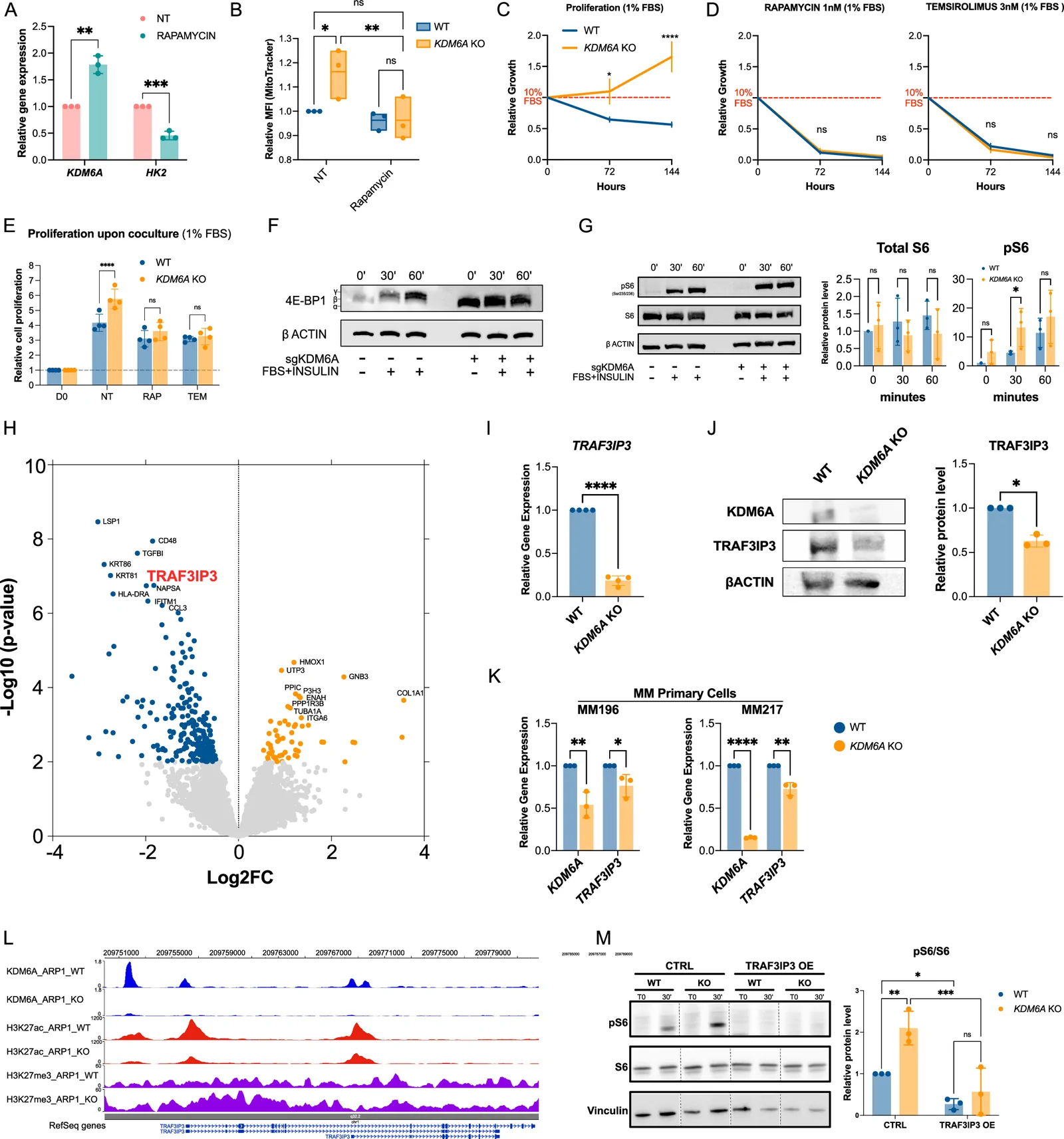
TRAF3IP3 is a KDM6A target regulating mTOR activity. (**A**) Gene expression of *KDM6A* and *HK2* analyzed by RT-qPCR after treatment with Rapamycin (50 nM) for 24 h in ARP-1 cells. Data are normalized to *TBP* and to non-treated (NT) condition and represented as mean +/− SD of three independent biological replicates. Statistical significance was assessed by two-tailed unpaired t-test (**p = 0.0012, ***p = 0.0003). (**B**) MFI of MitoTracker^TM^ measured on cocultured ARP-1 cells treated with Rapamycin 50 nM. Data are normalized to NT-WT. Significant differences were quantified by applying two-way ANOVA test (*n* = 3; *p = 0.0212, **p = 0.0081, ns *p* = 0.5391 and *p* = >0.9999). NT-WT: Min = 1, Median = 1, Max = 1; NT-KO: Min = 1.05, Median = 1.19, Max = 1.25; Rapa-WT: Min = 0.92, Median = 0.98, Max = 0.99; Rapa-KO: Min = 0.89, Median = 0.94, Max = 1.06. (**C**) Proliferation evaluation of WT and *KDM6A* KO ARP-1 cell line in 1% FBS growth medium, normalized on 10% FBS growth medium results. Proliferation was also assessed after treatments administration with (**D**) Rapamycin 1 nM and Temsirolimus 3 nM. Significant differences were quantified by applying two-way ANOVA test (**C**: *p = 0.03122, ****p = 0.00008; **D**: ns *p* = 0.3263, ns *p* = 0.3809, ns *p* = 0.2048, ns *p* = 0.4532). (**E**) Proliferation differences in ARP-1 cell line co-cultured with HS-5 in medium with 1% of FBS, after treatment with 1 nM Rapamycin (RAP) and 3 nM Temsirolimus (TEM). Significant differences from four independent biological replicates were quantified by applying two-way ANOVA test (****p = 0.00007, ns *p* = 0.15793, ns *p* = 0.61893). (**F**) One representative out of three western blot evaluations of the mTORC1 target 4E-BP1 after starvation and induction with FBS and insulin at different time points. Three phosphorylation isoforms of 4E-BP1, and the β ACTIN control are showed. (**G**) Representative western blot evaluation and quantification of the mTORC1 target S6 after starvation and induction with FBS and insulin at different time points. Relative protein levels of S6 and pS6, of three independent biological replicates normalized to βACTIN. Mean +/− SD. Significant differences were quantified by applying unpaired one-tailed t-test (S6: ns *p* = 0.3455, ns *p* = 0.2241, ns *p* = 0.1653; pS6 ns *p* = 0.0957, *p = 0.0427, ns *p* = 0.2063). (**H**) Volcano Plot showing DEGs in *KDM6A* KO compared to WT ARP-1 cell lines (*n* = 4, limma, *p* < 0.01). (**I**) RNA levels of *TRAF3IP3* were measured through RT-qPCR in ARP-1 cells. Data are normalized to *TBP* and to WT and represented as mean +/− SD of four independent biological replicates. Significant differences were quantified by one sample t-test (****p = 0.00008). (**J**) One representative western blot evaluation of KDM6A and TRAF3IP3 in ARP-1 cells. Relative protein levels of TRAF3IP3 were measured in three independent biological replicates and normalized to βACTIN. Mean +/− SD. Significant differences were quantified by one sample t-test (*p = 0.0108). (**K**) *KDM6A* and *TRAF3IP3* expression evaluation by RT-qPCR on MM196 and MM217 MM primary cell lines. Normalization on *ACTB* and WT control. Mean +/− SD; multiple unpaired t test (MM196: **p = 0.0062, *p = 0.0381; MM217: ****p = 0.000000004, **p = 0.003406752). (**L**) ChIPseq peaks of KDM6A, H3K27ac and H3K27me3 on *TRAF3IP3* genomic region in ARP-1 WT and *KDM6A* KO. (**M**) One representative out of three western blot evaluations of mTORC1 targets in control (CTRL) and *TRAF3IP3* overexpressed WT and *KDM6A* KO ARP-1 cell lines. Targets were evaluated after starvation and mTORC1 induction with FBS and insulin at different time points. Protein levels of pS6 were normalized on S6 and Vinculin. Mean +/− SD. Significant differences were quantified by applying two-way ANOVA test. Mean +/− SD, *n* = 3 (**p = 0.0054, *p = 0.036, ***p = 0.0008, ns *p* = 3351.). Source data are available online for this figure.

**Figure EV6:**
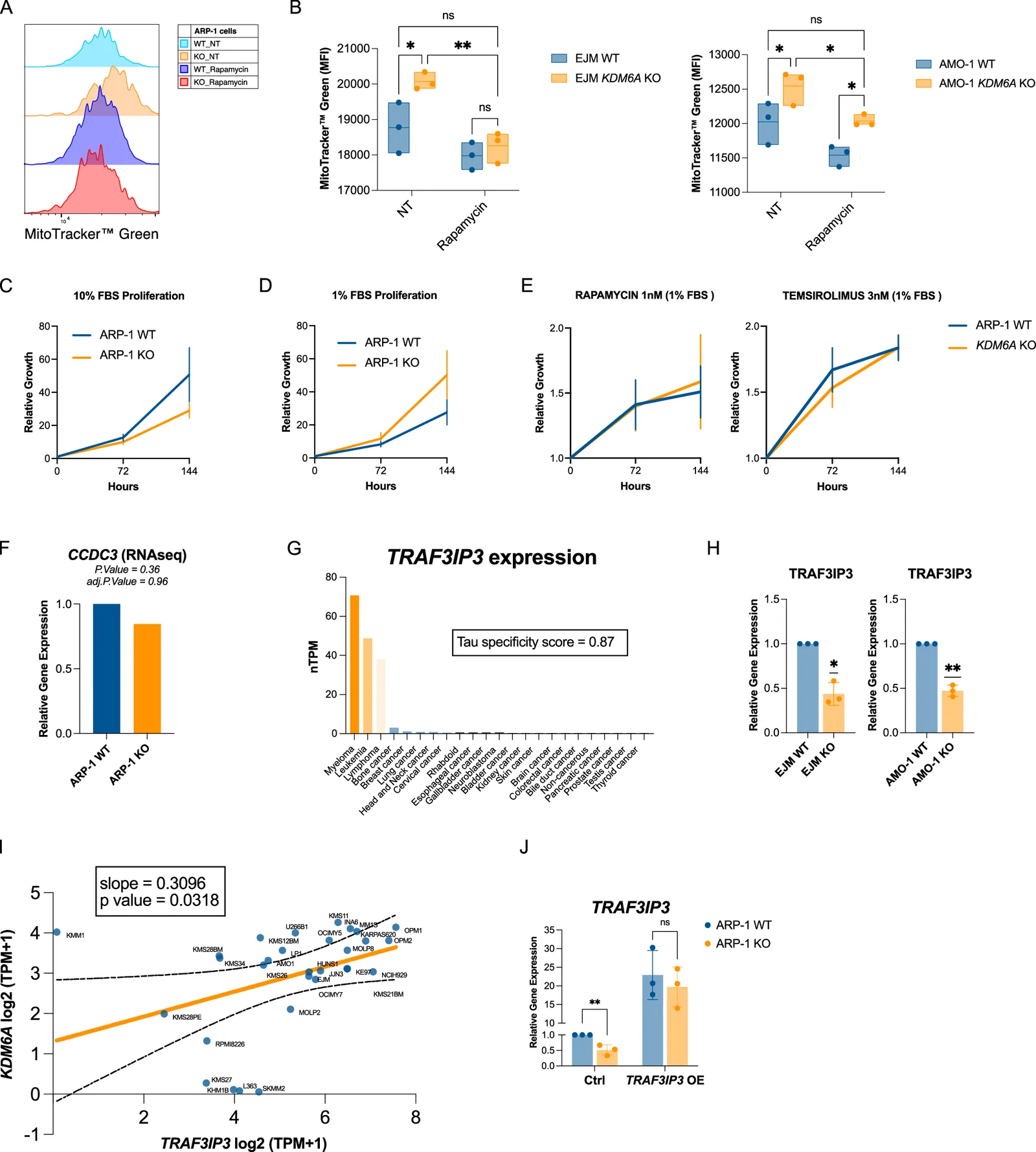
*KDM6A* KO in MM cell lines downregulates the mTOR inhibitor TRAF3IP3 to induce proliferation and mitochondrial trafficking. (**A**) One representative MFI measurement by FACS of MitoTracker^TM^ Green in ARP-1 cells upon *KDM6A* KO and Rapamycin 50 nM treatment. (**B**) MFI of MitoTracker^TM^ measured on HS-5 cocultured EJM and AMO-1 cells treated with Rapamycin 50 nM. Data are normalized to WT and represented as mean +/− SD of three independent biological replicates. Significant differences were quantified by applying two-way ANOVA test (EJM: *p = 0.0103, ns *p* = 0.2228, **p = 0.0016, ns *p* = 4926; AMO-1: *p = 0.018, ns *p* = 0.9458, *p = 0.0201, *p = 0225). (EJM) NT-WT: Min = 18,053, Median = 18,784, Max=19,481; NT-KO: Min = 19,873, Median = 20,005, Max = 20,340; Rapa-WT: Min = 17,583, Median = 17,994, Max = 18,354; Rapa-KO: Min = 17,758, Median = 18,415, Max= 18,599. (AMO-1) NT-WT: Min = 11,690, Median = 12,092, Max=12290; NT-KO: Min = 12,259, Median = 12,664, Max = 12,712; Rapa-WT: Min = 11,373, Median = 11,588, Max = 11,661; Rapa-KO: Min = 11,986, Median = 11,986, Max = 12,137. (**C**) Proliferation evaluation of WT and KO ARP-1 cell line in 10% FBS growth medium. Mean +/− SEM (*n* = 3). (**D**) Proliferation evaluation of WT and KO ARP-1 cell line in 1% FBS growth medium. Mean +/− SEM (*n* = 3). (**E**) Proliferation evaluation of WT and KO ARP-1 cell line in 1% FBS growth medium during treatment with Rapamycin 1 nM and Temsirolimus 3 nM. Mean +/− SEM (*n* = 3). (**F**) Differential gene expression of *CCDC3* from RNAseq in ARP-1 cells upon *KDM6A* KO (*n* = 4, limma). (**G**) Normalized Transcript per Million (nTPM) values of cancer cell lines categories ranked for *TRAF3IP3* expression levels. Tau specificity score is indicated. (**H**) Gene expression levels of *TRAF3IP3* were measured through RT-qPCR. Data are normalized to *TBP* and to WT and represented as mean +/− SD of three independent biological replicates. Significant differences were quantified by one sample t-test (*p = 0.0167, **p = 0.0049). (**I**) Linear regression of *KDM6A* and *TRAF3IP3* expression across different MM cell lines from DepMap. (**J**) Gene expression analyzed by RT-qPCR of *TRAF3IP3* in control and *TRAF3IP3* overexpressed ARP-1 cells. Data are normalized to *TBP* and to WT and represented as mean +/− SD of three independent biological replicates. Statistical significance was assessed by applying multiple unpaired t tests (**p = 0.0074, ns *p* = 0.5532).

Treatment of the MM cells with mTORC1 inhibitor Rapamycin blunted the expression of *HK2*, suggesting an involvement of mTORC1 in regulating the deregulation of glycolysis (Fig. 6A). Intriguingly, mTORC1 inhibition also elicited the expression of *KDM6A*, further suggesting a contrasting activity of mTOR and KDM6A in controlling glycolysis. mTORC1 inhibition even blocked the increase in transferred mitochondria, bringing them back to basal levels in *KDM6A* KO MM cells cocultured with HS-5 (Figs. 6B and EV6A). This phenotype was also observed in two additional MM cell lines (EJM and AMO-1) (Fig. EV6B).

mTORC1 activation increases cellular proliferation and growth (Saxton and Sabatini, 2017), and yet *KDM6A* KO cells proliferated more slowly than *KDM6A* WT when cells were grown at 10% FBS (Fig. EV6C). Since mTORC1 is activated by FBS addition we lowered FBS concentration. Under these conditions, *KDM6A* KO cells grew much faster than WT cells (Figs. 6C and EV6D). Treatment with the mTORC1 inhibitors Rapamycin or Temsirolimus ablated the increased proliferation elicited by *KDM6A* (Figs. 6D and EV6E), also in coculture conditions (Fig. 6E).

Prompted by these results, we then sought to confirm the activation of mTORC1 after *KDM6A* KO. We detected an increase on the phosphorylation status of the mTORC1 targets eIF4E-binding protein1 (4E-BP1) and of the ribosomal protein S6 (S6) under mTOR stimulation by serum starvation and re-addition. Specifically, the 3 phosphorylated forms (α, β, γ) of 4E-BP1 were strongly increased at baseline in *KDM6A* KO cells compared to WT cells (Fig. 6F) and were further increased after serum stimulation. Similarly, phospho-S6 (pS6) levels were significantly higher starting at 30’ after serum addition in KO cells (Fig. 6G), suggesting that *KDM6A* loss triggers a robust mTORC1 activation in MM cells.

To determine how *KDM6A* loss activates mTORC1 in MM, we examined RNAseq data, generated in *KDM6A* KO and WT ARP-1 cells. Unlike the findings reported in U-2 OS cells, no changes were evident on *CCDC3* expression levels after *KDM6A* KO (Fig. EV6F). We thus expanded the analysis to the entire dataset and identified TRAF3 Interacting Protein 3 (*TRAF3IP3*) among the top downregulated genes in *KDM6A* KO cells (Fig. 6H). The adapter protein TRAF3IP3 has been shown to reduce mTORC1 in regulatory T cells (Yu et al, 2018), through the localization of the catalytic subunit of the phosphatase PP2 (PP2ac) on the lysosome surface, thus impeding mTORC1 activity (Yu et al, 2018). Intriguingly, a search in the Human Protein Atlas uncovered that the TRAF3IP3 protein is specifically expressed by myeloma, leukemia, and lymphoma, but not by epithelial cancer cell lines (Tau specificity score = 0.87) (Fig. EV6G).

We first confirmed that *TRAF3IP3* RNA and protein levels were reduced in *KDM6A* KO ARP-1 cells (Fig. 6I,J). To further support the connection between KDM6A and *TRAF3IP3* expression, we validated the downregulation of *TRAF3IP3* in EJM and AMO-1 cell lines (Fig. EV6H). Moreover, we explored the association of expression of these two genes in large datasets, using the DepMap repository (Tsherniak et al, 2017). This analysis revealed a significant linear correlation between the expression of the two genes specifically in MM cell lines (Fig. EV6I). Finally, we confirmed *TRAF3IP3* downregulation upon *KDM6A* KO in the two primary MM cell lines MM196 and MM217 (Fig. 6K).

We then ascertained whether KDM6A bound to the *TRAF3IP3* locus on the genome. To this end, we reanalyzed our prior ChIPseq data of KDM6A, H3K27me3, and H3K27ac on ARP-1 cells (Dupéré-Richer et al, 2024). On the *TRAF3IP3* locus, KDM6A bound to three regions upstream and within the *TRAF3IP3* gene, and colocalized with loci of increased H3K27 acetylation (Fig. 6L), suggesting that KDM6A binds to enhancers controlling *TRAF3IP3* expression, in line with our previous findings highlighting a role of KDM6A on preferentially modulating enhancers (Dupéré-Richer et al, 2024). In *KDM6A* KO cells, H3K27ac was reduced at the *TRAF3IP3* locus, while the repressive histone mark H3K27me3 was increased throughout the *TRAF3IP3* gene body (Fig. 6L), suggesting in all that KDM6A controls *TRAF3IP3* transcription through the modulation of both histone acetylation and methylation at these enhancer and promoter sites.

To conclusively demonstrate that KDM6A controls mTORC1 through TRAF3IP3 in MM cells, we overexpressed *TRAF3IP3* in *KDM6A* KO ARP-1 cells. Overexpression of *TRAF3IP3* (Fig. EV6J) prevented the phosphorylation of the mTORC1 target S6 elicited by *KDM6A* KO (Fig. 6M).

All together, these data suggest that KDM6A reduces mTORC1 signaling via TRAF3IP3 in MM cells. Loss of KDM6A is associated with a reduction of TRAF3IP3, which in turn unleashes mTORC1, triggering glycolysis and stimulating the import of mitochondria from adjacent stromal cells, boosting MM oxidative metabolism and cell growth.

### Kdm6a controls OXPHOS also in normal tissues

**Figure EV7:**
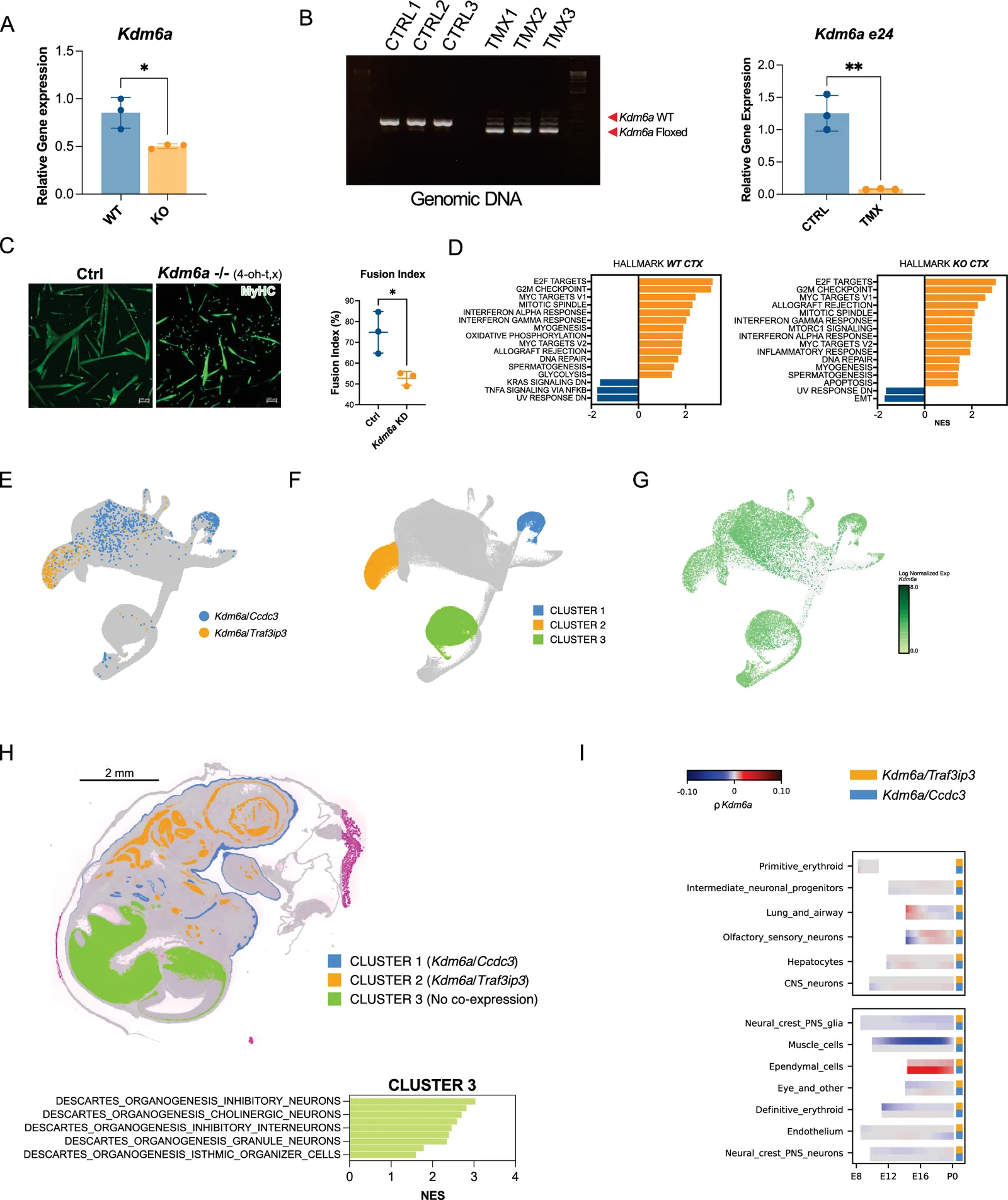
*Kdm6a* is central for muscle differentiation and its co-expression with *Ccdc3 or* *Traf3ip3 *identifies specific clusters of cells belonging to different embryonic tissues. (**A**) Expression levels assessed by RT-qPCR of murine *Kdm6a* in C2C12 cell line. Mean +/− SEM (*n* = 3). Unpaired t test (*p = 0.02). (**B**) On the left, electrophoretic run of genomic DNA highlighting *Kdm6a* exon24 deletion after tamoxifen treatment. On the right, gene expression of *Kdm6a* (exon 24) assessed by RT-qPCR. Normalized on *GAPDH*. Mean +/− SD of three independent biological replicates. Unpaired t test (**p = 0.0018) (**C**) One representative image of MyHC (Myosin Heavy Chain) staining on satellite cells from mice. Fusion index percentage of control and *Kdm6a* KD cells. Mean +/− SD (*n* = 3). Unpaired t test (*p = 0.0222). (**D**) GSEA (v4.3.2) of DEGs from RNAseq data collected in WT and *Kdm6a* KO SCs after CTX treatment. Pathways derived from HALLMARK. (**E**) UMAP representation of spatial transcriptomic data showing *Kdm6a/Ccdc3* and *Kdm6a/Traf3ip*3 co-expressions, while (**F**) shows the *Kdm6a/Ccdc3*, *Kdm6a/Traf3ip3* and a third unknown cluster and (**G**) represents *Kdm6a* expression. (**H**) H&E staining and clustering of spatial transcriptomics data from mouse embryo at day 15.5. GSEA, v4.3.2 was performed on the genes differentially expressed within the cluster 3. (**I**) Heatmap showing the co-expression patterns of *Kdm6a* (ρKdm6a) with *Traf3ip3* and *Ccdc3* across others embryonic tissues during development.

**Figure 7:**
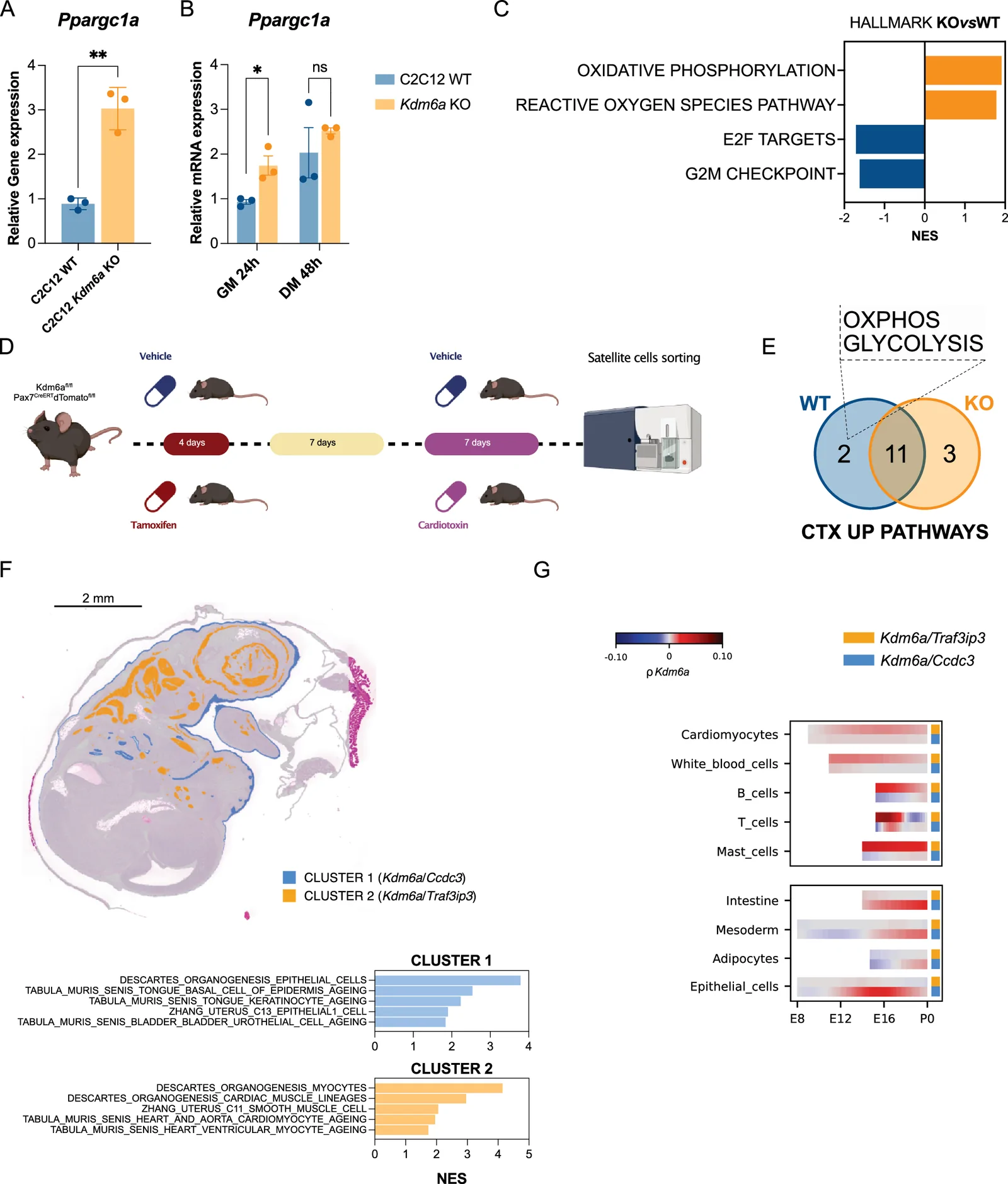
Kdm6a tames OXPHOS in muscle development and evokes convergent evolution at a tissue level. (**A**) RNA levels measured by RT-qPCR of murine *Ppargc1a* in WT and *Kdm6a* KO C2C12 cells. Three independent biological replicates, mean +/− SEM. Unpaired t test (**p = 0.0017). (**B**) *Ppargc1a* expression assessed by RT-qPCR in C2C12 from growth medium (GM) to differentiation medium (DM). Three independent biological replicates, mean +/− SEM. Multiple unpaired t test (*p = 0.0217, ns *p* = 0.4353). (**C**) GSEA (v4.3.2) analysis of transcriptome data from KO and WT mice comparison. Pathways were derived from HALLMARK. (**D**) Schematic representation of in vivo *Kdm6a* KO and cardiotoxin (CTX) treatments. (**E**) Venn Diagram recapitulating common and uncommon upregulated pathways from GSEA (v4.3.2) analysis performed on CTX treated mice. (**F**) H&E staining and clustering of spatial transcriptomics data from mouse embryo at day 15.5. Each cluster represents a group of cells in which *Ccdc3* or *Traf3ip3* is co-expressed with *Kdm6a*. GSEA, v4.3.2 was performed on the genes differentially expressed within the two identified clusters. (**G**) Heatmap showing the co-expression patterns of *Kdm6a* (ρKdm6a) with *Traf3ip3* and *Ccdc3* across various embryonic tissues. Co-expression levels are shown along the embryonic developmental timeline from embryonic day (E) 8 to postnatal day (P) 0. Source data are available online for this figure.

To ascertain whether *KDM6A* regulation of OXPHOS is a feature limited to cancer cells or is present also in healthy tissues, we exploited the immortalized but non-transformed C2C12 murine myoblast cell line (Blau et al, 1983), given the established role of Kdm6a in driving muscle differentiation (Seenundun et al, 2010). CRISPR-Cas9 was used to disrupt the *Kdm6a* gene in C2C12 (Fig. EV7A). Notably, as in cancer cells, *Kdm6a* KO in the undifferentiated muscle cell line increased *Ppargc1a* expression (Fig. 7A). During C2C12 differentiation, *Ppargc1a* RNA were comparable between WT cells and *Kdm6a* KO cells (Fig. 7B).

To confirm these in vitro data also in an in vivo, physiological setting, we exploited a mouse model employed to study the effect of Kdm6a on muscle differentiation (Faralli et al, 2016). This is a mouse model where *Kdm6a* is conditionally deleted by tamoxifen (TMX) administration in Pax7 positive satellite cells (SCs), the adult stem cells responsible for muscle regeneration. SCs obtained from mice treated with TMX (Fig. EV7B) were unable to differentiate into myotubes (Fig. EV7C). RNA-seq analysis of WT and *Kdm6a* KO SCs revealed again that OXPHOS was the main upregulated pathway upon *Kdm6a* deletion (Fig. 7C). To induce in vivo muscle differentiation, WT and conditional KD mice were treated with cardiotoxin (CTX) (Fig. 7D). Seven days after CTX treatment, SCs were isolated from regenerating muscle and RNAseq performed. Interestingly, treatment-induced muscle regeneration modulated very similar pathways in both WT and *Kdm6a* KD cells (Fig. EV7D), with two exceptions, the OXPHOS and the glycolytic pathways, which were induced in WT cells but not in *Kdm6a* KD cells (Fig. 7E), mimicking the results obtained with C2C12 cells.

In all, these results suggest that Kdm6a also controls OXPHOS in normal tissues.

### Convergent tissue-level evolution on OXPHOS through Kdm6a

Our results in various cancer types suggest that KDM6A tames OXPHOS through two alternative pathways, one centered on CCDC3, and another one leveraging TRAF3IP3 to control mTOR activation. Therefore, we questioned whether a similar, differential engagement was evident also during development. To this end, we exploited spatial transcriptomics mouse embryo (E15.5) data and assessed the co-expression of *Kdm6a* with *Ccdc3* and *Traf3ip3*, respectively. This analysis revealed that cells expressing *Kdm6a* with *Ccdc3* or *Traf3ip3* belong to independent clusters (Fig. EV7E). We mainly identified 3 clusters (Fig. EV7F) all expressing *Kdm6a* (Fig. EV7G). The first cluster was characterized by cells presenting the concomitant expression of *Kdm6a* and *Ccdc3* and it was for a large part of epithelial origin. A second cluster included cells expressing *Kdm6a* alongside *Traf3ip3*: this belonged to the muscle tissues (Fig. 7F). A third cluster showed cells expressing *Kdm6a*, but not *Ccdc3* nor *Traf3ip3*, with the central nervous system as reference tissue (Fig. EV7H).

We then extended the analysis following the entire span of murine development through single-cell RNAseq data (Qiu et al, 2024). Several alternative patterns of co-expression between *Kdm6A, Ccdc3* and *Traf3ip3* were apparent. Tissues in which *Kdm6a*/*Traf3ip3* co-expression was dominant included cardiomyocytes, white cells, B cells, T cells, mast cells. In other tissues like intestine, adipocytes, epithelial cells and not fully differentiated mesoderm, *Kdm6a*/*Ccdc3* co-expression was predominant showing a pattern of mutual exclusivity with *Traf3ip3* (Fig. 7G). Other tissues mainly involved in the nervous system displayed a less defined behavior (Fig. EV7I). In all, these results suggest that a single protein (KDM6A) has been selected to control the same pathway (OXPHOS) but can do so by partnering with different proteins. This indicates a differential strategy of gene and metabolic regulation acting at the tissue level during development, and not at the organism level. We could define this phenomenon as a “tissue-level convergent evolution”.

## Discussion

A large body of literature has conclusively demonstrated that metabolic changes can profoundly impact on chromatin landscape (Dai et al, 2020), often through the modulation of histone demethylases (Chang et al, 2019). Much less evidence is available on whether, and how, chromatin modifiers may affect metabolism. In this study, we have found that both in cancer and normal tissues KDM6A controls OXPHOS through different mechanisms, depending on the tissue context. As such, KDM6A appears to be a central hub upon which selection has acted to modulate OXPHOS.

For almost a century, the Warburg effect, that is, enhanced aerobic glycolysis, has been considered the defining metabolic feature of cancer cells (Semenza et al, 2001). However, emerging results indicate that OXPHOS in specific tumor types and conditions is also essential in driving tumorigenesis (Kuntz et al, 2017; Rao et al, 2019; Lissanu Deribe et al, 2018). Our results suggest that cancer cells harness KDM6A loss to elicit OXPHOS, though two entirely different paths, depending on the cellular context. One is straightforward and is exploited by solid tumors. Through the loss of KDM6A, cells harness CREB1 via a poorly studied protein, CCDC3, to increase the expression of *PPARGC1A*, thus increasing mitochondrial mass and OXPHOS.

MM cells instead exploit a more cunning mechanism. In this setting, KDM6A loss triggers mTORC1, which per se activates glycolysis and in several contexts block OXPHOS (that is in fact usually elicited by AMPK, the mTOR antagonist). It is likely that within the bone marrow microenvironment, a strong selective pressure exists to increase OXPHOS in cancer cells, possibly related to proliferation advantages, as shown by the landmark study of Spees et al (2006), and extensively confirmed in subsequent studies (Berridge et al, 2025; Borcherding and Brestoff, 2023). MM cells hence exploit a less traveled, more obscure function of mTORC1, that is, promoting the retrieval of mitochondria from nearby healthy cells via TNTs, while not harnessing 4EBPs to increase mitochondrial biogenesis (Morita et al, 2013). While we do not have in vivo proofs, it is tempting to speculate that MM cancer cells therefore use KDM6A loss to exploit both increased glycolysis and OXPHOS, managing to concomitantly activate two pathways, often considered competing, leveraging a single, central node, mTORC1. Hence, MM cells may be able to fully leverage on two pathways covering a broad range of metabolic requirements, to become more aggressive and adaptable to the most severe environmental constrains. As to interfering with the OXPHOS activation and mitochondrial trafficking, we have attempted two pharmacological approaches. mTORC1 inhibitors were effective in hampering the growth of MM cells lacking KDM6A, however, their reported toxicity may limit their application in this context. Inhibiting OXPHOS with the IACS-010759 inhibitor paradoxically increased viability of MM cells KO for KDM6A, suggesting that alternative strategies would need to be implemented to exploit the potential metabolic liability conferred by KDM6A loss.

An additional layer of context-specificity rests on the mechanism exploited by KDM6A to regulate its downstream targets. In the case of solid tumors, our data show that the control of gene expression is not mediated by KDM6A demethylase activity, since a mutant lacking catalytic property was equally apt to modulate *PPARGC1A* levels than the WT isoform. These results are in line with a large body of literature, showing that in several instances KDM6A acts as a scaffolding protein and does not rely on its demethylase activity (Gozdecka et al, 2018; Pacella et al, 2025). Conversely, in MM, our results based on the ChIP-seq data suggest that KDM6A catalytic activity is required to control *TRAF3IP3* expression.

pVHL loss in clear cell renal cancers, hemangioblastomas and paragangliomas, generates a pseudo-hypoxic state, unleashing HIF expression despite a normoxic environment. Our findings, showing the association between KDM6A loss and increased OXPHOS, strongly suggest that in several cancer types KDM6A loss triggers the opposite phenotype, that is, a pseudo-hyperoxic state. Another example of this pseudo-hyperoxic state is provided by the SWI/SNF member SMARCA4, frequently altered by loss-of-function mutations in lung adenocarcinoma. Cancer cells lacking SMARCA4 display increased mitochondrial mass through an increase in *PPARGC1A/PGC-1α* and become dependent on OXPHOS for their survival (Lissanu Deribe et al, 2018). We propose a new paradigm used by cancer cells lacking KDM6A, SMARCA4A and potentially other genes that generates a pseudo-hyperoxic state, which fosters OXPHOS despite normal levels of oxygen within the tumors.

Our results also suggest that a histone demethylase, KDM6A has been selected to regulate a key cellular metabolic node, OXPHOS, leveraging two entirely different avenues in various tissues. Why has evolution chosen a histone demethylase as a key controller of such a crucial cellular crossroad? One potential explanation stems from the fact that in animals and land plants, there is a limited set of proteins bestowed with oxygen-sensing capabilities, and these are for the most part enzymes, ultimately controlling TF activity though the regulation of their protein stability (Hammarlund et al, 2020). KDM6A being α-ketoglutarate and Fe2^+^ dependent oxygenase with low oxygen affinity is an oxygen-sensing enzyme thus representing an ideal protein to control OXPHOS depending on the cellular context.

Natural selection is a constrained process: it works on already existing material, like the limited set of proteins with oxygen-sensing capabilities. Nevertheless, the molecular basis of adaptive change can be very different. According to Natarajan et al (2016), even in cases where adaptive phenotypic change is predictable (i.e., the increases in hemoglobin-oxygen binding affinity in alpine species of birds), the molecular changes underlying the hemoglobin changes were variable and unpredictable. Here we show that even when the starting protein is the same (KDM6A), the molecular pathways leading to the same function (OXPHOS) can be different and mutually exclusive, involving different tissues during development. Convergent evolution is the process by which unrelated or distantly related species evolve functionally analogous adaptive traits, due to similar selective pressures, starting from different initial conditions. In terms of proximate (mechanical) causes, we observe here a highly functional trait (OXPHOS) that is activated during development through two mutually exclusive pathways in different tissues, both starting with the same protein KDM6A. According to evolutionary developmental biology (Evo-Devo), this means, in terms of remote (evolutionary) causes, that different partner proteins have been recruited to reach the same adaptive pathway (KDM6A to OXPHOS). While phenotypic convergence posits that different combinations of transcription factors modulate the same effector genes in different cell types (Wang et al, 2025; Konstantinides et al, 2018), the case of KDM6A seems different. Tissue-specific constrains channeled evolution to control OXPHOS leveraging two different molecular paths, one through TRAF3IP3 and mTORC1, and a second exploiting the sticky coil-coil domain of a bystander (CCDC3) to harness an established controller of mitochondrial biogenesis such as CREB1 and PPARGC1A. To our knowledge, this evolutionary strategy, anchored at the start (KDM6A) and at the end points (OXPHOS) in different tissues of the same organism, has not been described previously. Given this double constraint, we named it tissue-level convergent evolution, where various partners interact with KDM6A to reach the same endpoint. Finally, cancer cells actively change the surrounding environment. In evolutionary biology, this is termed niche construction. Our results confirm that tumors lacking KDM6A are niche constructors (through TNTs in this case), as they not only adapt, but also change their tissue environment.

## Methods

**Table:** Reagents and tools table

Reagent/Resource	Reference or Source	Identifier or Catalog Number
**Experimental models**		
Human U-2 OS	ATCC	Cat# HTB-96
Human SW780	Bernassola lab	RRID:CVCL_1728
Human HS-5	Ghia lab	RRID:CVCL_3720
Human PaTu8988t	Kiemmelman lab	RRID:CVCL_1847
Mouse C2C12	Madaro lab	RRID:CVCL_0188
Human ARP-1	Licht lab	RRID:CVCL_D523
Human EJM	Licht lab	RRID:CVCL_2030
Human AMO-1	Licht lab	RRID:CVCL_1806
Human MM196	https://doi.org/10.1182/bloodadvances.2020002566.	N/A
Human MM217	https://doi.org/10.1182/bloodadvances.2020002566.	N/A
Human HEK293T	Naldini lab	RRID:CVCL_0063
Mouse *Pax7*^*CreER*^*Utx*^*fl/fl*^	https://doi.org/10.1172/JCI83239	N/A
**Recombinant DNA**		
pLenti-c-Myc-GFP	Addgene	Cat# 123299
pLenti-TRAF3IP3	Vector Builder	VB900018-7678emt
pTRE-Tight-MitoTimer	Addgene	Cat# 50547
pLenti-PPARGC1Ap-GFP	This paper	N/A
pLenti-PGK V5-LUC-Neo (w623-2)	Addgene	Cat# 21471
pCS2-UTX-F-MT2	Addgene	Cat# 40619
pCS2-UTX-F	Addgene	Cat# 17438
pCS2FLAG	Addgene	Cat# 16331
**Antibodies**		
Anti-CCDC3	Merck	Cat#SAB1301135
CREB (86B10) Mouse	Cell Signaling	Cat #9104
CREB (D76D11) Rabbit	Cell Signaling	Cat #4820
KDM6A/UTX (D3Q1I) AB_2721244	Cell Signaling	Cat# 33510
Alexa Fluor® 647 goat anti-rabbit	Invitrogen	Cat# A-21245
Alexa Fluor® 488 goat anti-mouse	Invitrogen	Cat# A-11001
CD138 (Syndecan-1) Monoclonal Antibody (MI15), eFluor™ 450, eBioscience™	Invitrogen	Cat# 48-1388-42
4E-BP1 (53H11) AB_2097841	Cell Signaling	Cat# 9644
S6 Ribosomal Protein (5G10) AB_331355	Cell Signaling	Cat# 2217
Phospho-S6 Ribosomal Protein (Ser235/236) AB_331679	Cell Signaling	Cat# 2211
Anti-Syndecan-1 AB_11150990	Abcam	Cat# Ab128936
Anti-Tom20 AB_399595	BD Transduction Laboratories	Cat# 612278
TRAF3IP3/T3JAM (B-7)	Santa Cruz	Cat# Sc-398781
Vinculin AB_477629	Sigma-Aldrich	Cat# V9131
βActin AB_476744/ AB_262011	Sigma-Aldrich	Cat# A5441/A3854
ECL Rabbit IgG, HRP-linked whole Ab	GE Healthcare	Cat# NA9310-1ML/NA934-1ML
CD45	Invitrogen	Cat# 48-0451-82
CD31	Invitrogen	Cat# 48-0311-82
TER119	Invitrogen	Cat# 48-5921-82
Sca1	Invitrogen	Cat# 11-5981-82
Itga7	AbLab	Cat# R2F2
MF20 antibody	Developmental Studies Hybridoma Bank	Cat# N/A
**Oligonucleotides and other sequence-based reagents**		
shRNA sequences	This study	Table EV1
PCR primers	This study	Table EV1
**Chemicals, Enzymes and other reagents**		
DMEM high glucose	Euroclone	Cat# ECM0728L
DMEM	Gibco	Cat# 41966-029
FBS	Gibco	Cat# 10270106
OPTIMEM	Thermo Fisher Scientific	Cat# 31985047
Horse Serum	Gibco	Cat# 26050070
Chicken Embryo Extract	US Biological Life Sciences	Cat# C3999
DMEM high glucose GlutaMAX™ Supplement	Thermo Fisher Scientific	Cat# 61965026
PFA	MilliporeSigma	Cat# P6148
BSA	MilliporeSigma	Cat# A7030-100G
Penicillin/Streptomycin	Euroclone	Cat# ECB3001D
RPMI	Euroclone	Cat# ECB2000L/ECB3001D
SYBR^®^ green	Applied Biosystems™	Cat# 4364346
Ponceau S solution	Sigma-Aldrich	Cat# P7170-1L
PanReac	AppliChem	Cat# A0830,1000
Tween® 20	Sigma	Cat# P1379-1L
PEG-it Virus Precipitation Solution	Systembio	Cat# LV825A-1
Puromycin	Invitrogen	Cat# A1113803
Blasticidin	Euroclone	Cat# EMR098005
Oligofectamine^TM^ Reagent	Invitrogen	Cat# 12252-011
Lipofectamine® RNAiMAX	Invitrogen	Cat# 13778-075
Lipofectamine^TM^ 3000	Invitrogen	Cat# L3000008
Xenolight d-luciferin – k+ salt bioluminescent substrate	Perkin Elmer	Cat# 122799
Alt-R^®^ CRISPR-Cas9 crRNA	IDT	Cat# 1075927
Alt-R^®^ CRISPR-Cas9 tracrRNA ATTO™ 550	IDT	Cat# 1075927
Nuclease Free Duplex Buffer	IDT	Cat# 11-05-01-12
Alt-R^®^ S.p. HiFi Cas9 Nuclease V3	IDT	Cat# 1081060
Alt-R^®^ Cas9 Electroporation Enhancer	IDT	Cat# 1075915
Neon™ Transfection System 100 μL Kit	Invitrogen	Cat# MPK10025
MitoTracker Green FM dye	Thermo Fisher Scientific	Cat# M46750
MitoTracker™ Red CMXRos	Thermo Fisher Scientific	Cat# M7512
Tetrahylrhodamine Isothiocyanate	Invitrogen	Cat# R415
Nucleofector X Kit L	Lonza	Cat# V4XP
DAPI	Thermo Fisher Scientific	Cat# 62248/D1306
Hoechst 33258 solution	Sigma-Aldrich	Cat# 94403-1ML
XF DMEM Medium pH 7.4	Agilent Technologies	N/A
AMD3100	Genzyme (Sanofi)	Mozobil
Insulin	Sigma	Cat# I0516
MitoSOX^TM^ Red Mitochondrial Superoxide Indicator	Invitrogen™	Cat# M36008
Cell-Tak	Corning™	N/A
Tamoxifen	Sigma	Cat# T5648
Cardiotoxin	Laxotan	N/A
Collagenase A	Roche	Cat# 10103586001
Dispase II	Roche	Cat# 04942078001
DNase I	Roche	Cat# 11284932001
RNeasy^®^ Mini Kit	Qiagen®	Cat# 69504
RNAqueous^®^ - Micro Kit	Thermofisher Scientific	Cat# AM1931
Nanodrop 8000 spectrophotometer	Thermofisher Scientific	Cat# ND-8000-GL
DNeasy^®^ Blood & Tissue Kit	Qiagen®	Cat# 69504
ImProm-II™ Reverse Transcription System	Promega	Cat# A3800
Thermal Cycler	Applied Biosystems™	Cat# 2720
VIIA 7 Real-Time PCR System	Applied Biosystems™	Cat# 4453534
Mini-PROTEAN^®^ Tetra Vertical Electrophoresis Cell	Bio-Rad	Cat# 1658004
Amersham™ Protran™ Premium 0.45 µm NC	Sigma-Aldrich	Cat# 10600003
ChemiDoc™ Imaging System	Bio-Rad	Cat# 12003153
Neon™ Transfection System	Invitrogen™	Cat# MPK5000
Seahorse XFe96 Analyzer	Agilent Technologies	N/A
CyQUANT Cell Proliferation Assays	ThermoFisher Scientific	N/A
Mithras LB 94 microplate reader	Berthold Technologies	N/A
Lactate colorimetric/fluorometric assay kit	Biovision	Cat# K607-100
iMark™ microplate reader	Bio-Rad	N/A
Amaxa 4D	Lonza	Cat# AAF
esiRNA human CCDC3	Merck Life	Cat# EHU150521
esiRNA human CREB1	Merck Life	Cat# EHU092951
esiRNA human KDM6A	Merck Life	Cat# EHU115561
esiRNA RLUC	Merck Life	Cat# EHURLUC
esiRNA human PPARG	Merck Life	Cat# EHU097711
esiRNA human CRABP2	Merck Life	Cat# EHU032221
esiRNA human PLEKHA1	Merck Life	Cat# EHU137611
esiRNA human TPM1	Merck Life	Cat# EHU081911
esiRNA human WWC2	Merck Life	Cat# EHU047041
**Software**		
Biorender	Biorender	https://www.biorender.com
Fiji	NIH	https://imagej.net/software/fiji
Prism v10.3.0	GraphPad	https://www.graphpad.com
DepMap portal	DepMap portal	https://depmap.org/portal/
Flowjo software	BD	https://www.flowjo.com
Rstudio	Posit	https://posit.co/download/rstudio-desktop/
Seahorse Wave software	Agilent	https://www.agilent.com/en/product/cell-analysis
ChIP Atlas	NIG SUPERCOMPUTER SYSTEM and JST NBDC JPMJND2202	https://chip-atlas.org

### Cell culture

U-2 OS cell line was purchased from ATCC. SW780 cell line was a gift from Dr. Francesca Bernassola’s laboratory (Faculty of Medicine and Surgery Department of Experimental Medicine University of Rome “Tor Vergata”), and HS-5 cell line was gifted from Dr. Paolo Ghia’s laboratory (San Raffaele Scientific Institute, Milan, Italy). PaTu8988t cell line was a gift from Dr. Alec Kiemmelman laboratory (Department of Radiation Oncology, Perlmutter Cancer Center, New York University Grossman School of Medicine, New York, NY, USA). These cells were cultured in DMEM high glucose medium complemented with 10% FBS and 1% penicillin/streptomycin. C2C12 cell line was a gift from Dr. Luca Madaro (Sapienza University of Rome, Italy). These cells were cultured in DMEM high glucose, GlutaMAX™ supplement and complemented with 10% FBS and 1% penicillin/streptomycin. ARP-1, EJM and AMO-1 cell lines were available in Dr. Jonathan Licht's Laboratory. Cells were cultured in RPMI medium complemented with 10% FBS (20% for AMO-1) and 1% penicillin/streptomycin. HEK293T cell line for lentiviral vector production was a gift from Dr. Luigi Naldini’s Laboratory (San Raffaele Telethon Institute for Gene Therapy, Milan, Italy). HEK293T were cultured in DMEM high glucose medium complemented with 10% FBS and 1% penicillin/streptomycin. All the cell lines analyzed are female in order to avoid potential UTY (Greenfield et al, 1998) compensation.

### Primary cells

Human primary cell lines isolated from patients MM196 and MM217 (Bruno et al, 2020) were cultured in Optimem (Thermo Fisher Scientific) supplemented with 15% inactivated Fetal Bovine Serum (FBS) (Thermo Fisher Scientific), 2 mM glutamine, and 40 μg/ml gentamicin and cultured at 37 °C in a humidified atmosphere with 5% CO_2_. Mycoplasma contamination was periodically checked by polymerase chain reaction (PCR) analysis. All the cell lines were not listed in the ICLAC database of commonly misidentified cell lines and were authenticated at The San Martino Hospital Genomics Facility using short tandem repeat DNA profiling. The presence of the Epstein-Barr virus (EBV) was detected by PCR. Transfections were carried out by nucleofection using Amaxa 4D- Nucleofector X Kit L (Lonza, IT) by following the manufacturer’s instructions. Cells were analyzed 48 h after transfection by western blot (WB) or quantitative real-time PCR.

### Primary cells mitochondrial content analysis

After 48h from nucleofection, the mitochondrial content of MM primary cells was assessed by staining the cells with MitoTracker Red CMXRos (Thermo Fisher Scientific, M7512) and 20 μg/ml Hoechst dye 33258 (Sigma-Aldrich) for 30 minutes at 37 °C. Following staining, the cells were washed twice with PBS, fixed in absolute methanol, resuspended in PBS, and transferred to a 96-well PhenoPlate (Perkin Elmer, USA). The analysis was performed using the Opera Phenix High-Content System. Confocal images were acquired with a 40× objective lens. Final images were analyzed using Fiji (Schindelin et al, 2012).

### Mouse strains

Mouse strains used in this study were Pax^7CreER^Utx^fl/fl^ mice, gently provided by F. Jeffrey Dilworth (Faralli et al, 2016). All mice were maintained in a pathogen-free animal facility under standard 12 h light/12 h dark cycle at 21 °C with access to red house and to standard chow and water ad libitum. Three-month-old mice were used for ex vivo experiments. For the SCs isolation experiments, female Pax^7CreER^Utx^fl/fl^ mice were used. 3 mg of Tamoxifen (Sigma, T5648) suspended in corn oil was injected intraperitoneally (i.p.) each day for 4 days. After 7 d from the last injection, to induce muscle damage, 10 μM cardiotoxin (CTX) (Latoxan, Valence, France) dissolved in phosphate-buffered saline (PBS) was injected into the tibialis anterior (TA) and gastrocnemius (GA) muscles. Tissues were harvested 7 days after injury for cell isolation.

### Nucleic acids extraction

RNA was extracted from approximately 1 × 10^6^ cells lysed in RLT buffer + 2-Mercaptoethanol (1:100). RNA from lysates was extracted with the RNeasy^®^ Mini Kit, alternatively, RNAqueous^®^-Micro Kit was used when the starting cell material was <5 × 10^5^ cells. RNA concentration and quality was evaluated by a Nanodrop 8000 spectrophotometer. DNA was extracted from approximately 1 × 10^6^ cells frozen as dry pellet. Thawed pellets were processed with DNeasy^®^ Blood & Tissue Kit. DNA concentration and quality was evaluated by a Nanodrop 8000 spectrophotometer.

### RT-qPCR

Reverse transcription was performed with the ImProm-II™ Reverse Transcription System starting from 1 µg of RNA. The reaction ran into the Thermal Cycler (Applied Biosystems, 2720 Thermal Cycler). To perform qPCR, 10 ng of cDNA were loaded as triplicates in 96/384 well plates and mixed with SYBR^®^ green (2X) and exon-spanning primers (200 nM). For mitochondrial mass evaluation, 2 ng of genomic DNA were loaded as triplicates in 96/384 well plates and mixed with SYBR^®^ green (2X) and nuclear and mitochondrial genomic primers (200 nM). QPCR was performed using the VIIA 7 Real-Time PCR System. Primer sequences are listed in Table EV1.

### Immunoblotting

Cells were lysed in Laemmli buffer (50% glycerol, 1 M Tris-HCl pH 6.8, 10% SDS) in order to obtain a concentration of 2 × 10^7^ cells/mL. Lysates were sonicated with the ultrasonic homogenizers BANDELIN SONOPULS (amplitude 12%, 1 pulse, 15 s). Protein lysates from 3 × 10^5^ cells were loaded on Bio-Rad precast 4–15% or 4–20% polyacrylamide gels. Electrophoretic separation was performed in Mini-PROTEAN^®^ Tetra Vertical Electrophoresis Cell with a running buffer containing 10% tris-glycine and 0.1% SDS. The power supply (Bio-Rad PowerPac™ basic, #1645050) was set at 250 V. Transfer was performed in Mini-PROTEAN^®^ Tetra Vertical Electrophoresis Cell with a transfer buffer containing 10% tris-glycine, 20% methanol, and 0.05% SDS. Proteins were transferred on nitrocellulose membranes (Amersham™ Protran™ Premium 0.45 µm NC) at 110 V for 1 h. To check the quality of the transfer, membranes were stained with Ponceau S solution. Membranes were blocked in PBS, 5% nonfat dried milk powder (PanReac), 0.1% Tween® 20 for 30’–60’. Incubation with primary antibodies was overnight (antibodies are listed in Reagents and Tools Table). Secondary antibodies incubation was performed for 45’. Membranes were developed with the ChemiDoc™ Imaging System.

### pLenti pPPARGC1A – GFP reporter system

This plasmid was cloned by Genscript using as a template pLenti c-Myc GFP adding the *PPARGC1A* promoter sequence controlling the expression of GFP. The *PPARGC1A* promoter sequence derives from SwitchGear Genomics website and was predicted by the SwitchGear Transcription Start Site (TSS) algorithm.

### Lentiviral vector production

For lentivirus production, HEK293T cells were transfected according to the CaCl_2_ protocol. A mix containing 10 μg of transfer vector, 6.5 μg of packaging vector ΔR8.74, 3.5 μg of Env VSV-G, 2.5 μg of REV, ddH_2_O to 450 μL, 50 μL of 2.5 M CaCl_2_, and 500 μL of 2X HBS were added dropwise to HEK293T cells seeded on a 15 cm dish. The medium was replaced after 16 h. 24 h later the medium containing virus particles was collected and pelleted at 1200 rpm. The supernatant was filtered on a 0.45 μm filter and then incubated with PEG-it Virus Precipitation Solution 5X overnight at 4 °C to precipitate viral particles. After a centrifugation step at 1500 × *g* at 4 °C, the lentiviral pellet was resuspended in 1x PBS and stored in aliquots at −80 °C. Alternatively, HEK293T were transfected using Lipofectamine™ 3000 transfection agent following manufacturing procedures.

### Viral transduction

For sh*KDM6A* and pLenti pPPARGC1A – GFP reporter, 180,000 U-2 OS cells were plated in a 6-well plate and infected the day after with 1 mL of diluted lentiviral vectors (polybrene 8 μg/mL). After 4 h incubation, 500 µL of transduction medium was discarded and replaced by 1.5 mL of fresh transduction medium and incubated overnight. The next day, medium was replaced by fresh growth medium, and cells were subsequently selected with 1 μg/mL puromycin for at least 3 days. After 48 h cells were collected for experimental procedures. In case of double transduction experiments, immediately after the 3-day puromycin selection cells were plated in the same conditions and the day after shSCR and sh*PPARGC1A* were used for viral transduction as above. In this case, the antibiotic selection was performed with 10 μg/mL blasticidin for 5 days. At the end of blasticidin selection cells were collected. ShRNAs sequences are listed in Table EV1.

ARP-1 cells were plated (1,000,000 cells/w) in a 24-well plate and transduced through spinoculation (1800 RPM, acceleration 2, no break) for 1 h at 25 °C. The media was refreshed upon transduction. An antibiotic selection was performed the following day. For gene expression evaluation, RNA was extracted 3 days after the end of selection. To generate luciferase-expressing cells, ARP-1 were transduced with pLenti PGK V5-LUC Neo (w623-2) (Botrugno et al, 2019; Frenquelli et al, 2020). For *TRAF3IP3* overexpression two different lentiviruses were used: pLenti c-Myc GFP and pLenti *TRAF3IP3* (see Reagents and Tools table).

### siRNAs transfection

U-2 OS, PaTu 8988t, and SW780 cell lines were plated at 30% confluency and transfected using Oligofectamine^TM^ Reagent. Cells were transfected following the protocol found at: www.lifetechnologies.com/rnai. Cells were collected at each time-point: 24 h, 48 h, and 72 h. EsiRNAs are listed in Reagents and Tools table.

### Plasmid transfection

U-2 OS cells were transfected with Lipofectamine 3000 according to manufacturer’s protocol. Cells were incubated for 48 h.

### CRISPR-Cas9 gene knock-out

To perform CRISPR-Cas9 *KDM6A* deletion in MM cells, we designed a guide RNA (gRNA) targeting exon 4 of *KDM6A* (CAGCAUUAUCUGCAUACCAGGUUUUAGAGCUAUGCU) and transfected cells with or without the gRNA. We annealed a crRNA (Alt-R^®^ CRISPR-Cas9 crRNA) and a tracrRNA (Alt-R^®^ CRISPR-Cas9 tracrRNA, ATTO™ 550) to obtain the specific gRNA. For the annealing, crRNA and tracrRNA were equimolarly mixed (from 100 to 10 µM) in duplex buffer (Nuclease Free Duplex Buffer) and heated to 95 °C for 5’ in a thermo-cycler and cooled to 25 °C at a rate of 2 °C/min. Next, Cas9 (Alt-R^®^ S.p. HiFi Cas9 Nuclease V3) was added to the RNA duplex to a final concentration of 10 µM for each component at 25 °C for 5’. An electroporation oligo enhancer (Alt-R^®^ Cas9 Electroporation Enhancer) was added to the reaction (10 µM). MM cells (3–5 × 10^6^) were washed once in PBS and resuspended in 100 µL of buffer R (Neon™ Transfection System 100 μL Kit). The electroporation event was performed in 100 µL tips (Neon™ Transfection System 100 μL Kit) at the following conditions: 1 pulse, 1600 V, 20 ms. Cells were immediately plated in 60 mm dishes with 3–5 mL culture medium without pen/strep. After 1 week, *KDM6A* KO was checked by immunoblot.

C2C12 cells were transfected with RNA iMAX according to manufactures procedures. We designed a guide RNA (gRNA) targeting exon 4 of *Kdm6a* (TAGCATTATCTGCATACCAG) and we transfected cells with or without the gRNA. Cells were plated on a 96-well for clonal selection by serial dilutions. Wells with 1 cell per well were identified.

### MitoTracker™ staining

Cells were plated and allowed to adhere to the culture dish when necessary. The culture medium was replaced with fresh pre-warmed medium with MitoTracker™ Green FM dye following manufacturer’s instructions. After incubation cells were collected and analyzed by flow cytometry.

### Seahorse metabolic flux analysis

We measured OCR and ECAR by Seahorse XFe96 Analyzer using SeaHorse XF Cell Mito Stress Test Kit and Glycolysis Stress Test Kit, following the manufacturer’s instructions. The day before the assay, cells were seeded in 96-wells Seahorse cell culture microplates and incubated in a 5% CO_2_ incubator at 37 °C overnight at a density of 13,000 cells/well and 40,000 cells/well for U-2 OS and ARP-1 cells, respectively. The day of the assay, the culture medium was changed with XF DMEM Medium pH 7.4. The medium was supplemented with 10 mM glucose, 1 mM sodium pyruvate, and 2 mM L-glutamine for Mito Stress Test, with 2 mM L-glutamine for Glycolysis Stress Test. The plate was incubated at 37 °C for 1 h in a non-CO_2_ incubator. For the Mito Stress Test assay, after OCR baseline measurements oligomycin A (O), carbonyl cyanide 4-(trifluoromethoxy)phenylhydrazone (FCCP), and antimycin A/rotenone (AA/R) were added sequentially to each well, to reach the final concentrations of 1 μM O, 1.5 μM FCCP for ARP-1 cells or 2.2 μM FCCP for U-2 OS cells, and 0.5 μM, AA/R. Respiratory states were calculated as follows: basal respiration by subtracting the minimum OCR measurement after AA/R injection minute from the last OCR measurement before O injection; ATP production by subtracting the minimum OCR measurement after O injection from the last OCR measurement before O injection; maximal respiration by subtracting the minimum OCR measurement after AA/R injection from the maximal OCR measurement after FCCP injection. For the Glycolysis Stress Test assay, after ECAR baseline measurements, glucose, O, and 2-deoxy-glucose (2-DG) were added sequentially to each well to reach the final concentrations of 10 mM, 1 μM, and 50 mM, respectively. Glycolysis was calculated by subtracting the last ECAR measurement before glucose injection from the maximum ECAR measurement before O injection; the glycolytic capacity by subtracting the last ECAR measurement before glucose injection from the maximum ECAR measurement before 2-DG injection. Results were normalized by cell number using CyQUANT Cell Proliferation Assays. Results are mean ± SD of technical replicates, OCR data are expressed as pmol of oxygen per minute per arbitrary units (pmol/min/a.u.), ECAR data as mpH per minute per arbitrary units (mpH/min/a.u.). All the analyses were performed with the Agilent Seahorse Wave software. The Mito Stress Test and the Glycolysis Stress Test on U-2 OS were performed with a minimal number of technical replicates equal to 11 and 12, respectively. The Mito Stress Test and the Glycolysis Stress Test on ARP-1 were performed with a minimal number of technical replicates equal to 15 and 11, respectively.

### Starvation and mTOR induction

ARP-1 cells were serum starved for 20 h. After FBS starvation, cells were further starved in PBS (10 mM D-glucose, 1 mM CaCl_2_, 0.5 mM MgCl_2_) or alternatively in HBSS 1x (Ca, Mg) at 37 °C for 30’. Then medium was switched to a 20% FBS 100 nM insulin RPMI medium for 30’ and 60’. At the end of the growth stimulation samples were collected for immunoblot analysis.

### mTOR inhibitors treatment

ARP-1 cells were serum-starved overnight, and then 1000 cells/well were plated in a 96-well plate, in 100 µL of culture medium with either DMSO, 3 nM Temsirolimus, or 5 nM Rapamycin, in triplicates. DMSO was used as a control. After plating, cells were collected at three different time points: 0, 72, and 144 h. Cells were incubated for 10 min with luciferin (200X). Cell proliferation was analyzed as luminescence utilizing a Mithras LB 94 microplate reader. Proliferation was measured at 10% and 1% serum concentrations. In case of coculture mTOR inhibitors treatments, HS-5 were plated 24 h before day 0, at which time an ARP-1 (2X) solution was added.

### Lactate measurement

Cells were cultured for 96 h without splitting. Subsequently, a cell medium aliquot was analyzed with the lactate colorimetric/fluorometric assay kit. Lactate concentrations (nmol) were calculated using a standard curve built analyzing lactate samples of known concentration with the iMark™ microplate reader and measuring the OD (595 nm). Lactate concentration was calculated as:

$$\left({nmol}\times {volume}\right)\div{dilution}$$

### Cocultures

The HS-5 cell line was plated at day 0 for 24 h. Subsequently, ARP-1 cells were added to the HS-5 cell line at a ratio of 5:1 and cultured for 96 h. Supernatant was considered for analysis.

### Mitochondrial trafficking evaluation

The HS-5 cell line and ARP-1 were cocultured as described before. Once HS-5 cells adhered, they were stained with MitoTracker™ Green FM dye. After staining, HS-5 cells were washed 3 times in PBS. ARP-1 cells were added and co-cultured for 96 h, then stained for CD138 and analyzed by flow cytometry.

### Mitochondrial trafficking impairment 

Same procedures as in the mitochondrial trafficking evaluation were performed. Stained HS-5 cells were washed twice with culture media. After that, ARP-1 cells were added and treated with Rapamycin 50 nM and AMD3100 10 µM for 24 h, then the supernatant was collected and analyzed by flow cytometry.

### MitoSOX™

ARP-1 cells were cocultured for 96 h and stained with MitoSOX^TM^ Red Mitochondrial Superoxide Indicator following manufacturer’s instructions. After incubation cells were collected and analyzed by flow cytometry.

### MitoTimer

To assess mitochondrial transfer from HS-5 to ARP-1 cells, we used pTRE-Tight-MitoTimer fluorescent reporter protein (pTRE-Tight-MitoTimer was a gift from Roberta Gottlieb), under the control of a tetracycline response element (Hernandez et al, 2013). After transfection of donor HS-5 cells, synchronized expression of MitoTimer was achieved by addition of doxycycline (Dox) pulse for 2 h, followed by removal of any residual Dox and co-culture with ARP-1 WT and *KDM6A* KO cells. After 72 h, MitoTimer expression was used to detect mitochondria in recipient cells by monitoring protein as it matures from green to red. Cells were acquired at CytoFLEX LX (Beckman Coulter), analyzed by FlowJo and differences in the content of both new (green) and old (red) mitochondria among WT and *KDM6A* KO cells were evaluated.

### Tunneling nanotubes quantification

A round glass coverslip was placed into a 24-well plate and coated using Cell-Tak dissolved in a 0.1 M NaHCO₃ solution for 20 min at RT. ARP-1 cells were then plated along with HS-5 cells at densities of 500,000 cells/well and 100,000 cells/well, respectively, and resuspended in RPMI growth medium without FBS. After plating, cells were attached by centrifugation (1800 RPM, acceleration 2, brake 0) for 15 min. The medium was then replaced with RPMI containing 1% FBS, and cells were incubated for 24 h. The following day, cells were fixed in 4% PFA, washed 2 times with PBS, then permeabilized and blocked using a solution of PBS, 0.5% Triton, 0.1% BSA, and 5% NGS for 30 min at RT. Subsequently, after washing with PBS, primary antibodies resuspended in blocking buffer with PBS, 1% BSA, and 5% NGS, were added overnight. After washing with PBS, secondary antibodies were added for 30 min. Finally, the coverslip was mounted onto a microscope slide using ProLong Gold Antifade Mountant. Images were acquired through LEICA SP5 confocal microscope and TNTs were manually quantified using Fiji.

### siRNAs selection

In silico analysis was conducted by integrating two different databases and the RNAseq data collected on U-2 OS KD cells. We selected the statistically significant downregulated genes (adj. *p*-value < 0.01) and merged this information with DepMap portal, were we retrieved the genes anti-correlated with PPARGC1A/PGC1α. Finally, these data were merged with the data collected on ChIP Atlas, to select the target genes of KDM6A. Once the data were merged, we ranked the genes by KDM6A average binding score, provided by ChIP Atlas.

### Proximity ligation assay (PLA)

20,000 U-2 OS cells transfected with siRNA to deplete KDM6A, CCDC3 or a RLUC control were plated on slides. The next day, cells were fixed with 4% paraformaldehyde for 10 min, permeabilized with 0.3% Triton-X for 1 h and then proximity ligation assay was performed according to the manufacturer’s instructions (PLA Duolink Merck). Briefly, cells were incubated with the primary antibodies against CCDC3 (Merck) and CREB1 (86B10; Cell Signaling) overnight at 4 °C. Then, incubation with the probes was performed. After ligation and amplification, slides were mounted by using in situ mounting media with DAPI and acquired at 100× magnification by Axio Imager.A2 fluorescence microscope (Zeiss). Fiji was used to process each image. The PLA puncta were reliably isolated from the background and then counted. Quantification of nuclei proceeds similarly. The result was expressed as PLA puncta/nuclei. Significant differences among groups were calculated by applying an unpaired Student t-test.

### Transcription factors analysis

Transcription factors activity was assessed with decoupleR (Badia-i-Mompel et al, 2022), OmnipathR (Türei et al, 2016), and viper (Alvarez et al, 2016). A regulatory network specific to a specific pathway (Mitochondrial Biogenesis, Reactome identifier R-HSA-1592230) was retrieved from OmniPath. The viper algorithm was then applied to this network to assess transcription factor activity. For visualization, a bar plot was created displaying transcription factor activity scores with transcription factors ordered accordingly.

### Cell viability assay

U-2 OS cells were infected with either control shRNA (shSCR) or *KDM6A*-targeting shRNA (sh*KDM6A*) lentiviral particles and selected with puromycin for 3 days. Subsequently, 1000 cells per well were seeded in triplicate into 96-well plates in 200 μL of complete culture medium. Cell viability was assessed using the CyQUANT Cell Proliferation Assay (Thermo Fisher Scientific) according to the manufacturer’s instructions. Fluorescence was measured with a VICTOR microplate reader (PerkinElmer).

### Cell cycle analysis

For flow cytometric analysis of the cell cycle, U-2 OS cells were infected with shSCR or shKDM6A viruses and selected with puromycin. Forty-eight hours post-selection, cells were fixed in cold 70% ethanol for 30 min on ice. Fixed cells were washed with PBS containing 1% BSA, then resuspended in staining solution consisting of 50 μg/mL propidium iodide (PI; Merck) and 200 μg/mL RNase A (Euroclone). Samples were incubated at 37 °C for 30 min. A minimum of 20,000 events per sample were acquired using a flow cytometer, and cell cycle distribution was analyzed with FCS Express 6.0 software (De Novo Software).

### Transmission electron microscopy

Cells were fixed as monolayer in 2.5% glutaraldehyde in 0.1 M sodium cacodylate buffer pH 7.4 for 1 h at room temperature, washed three times with cacodylate buffer and fixed in 1% osmium tetroxide, 1.5% potassium ferrocyanide in 0.1 M cacodylate for 1 h on ice. After several washes in distilled water, samples were “en bloc” stained with 0.5% uranyl acetate in water overnight at 4 °C. Finally, samples were dehydrated in a graded ethanol series (30%, 50%, 70%, 80%, 90%, 96%, 5 min each and 3 washes with absolute ethanol, 10 min each). The samples were then infiltrated in a 1:1 ethanol/Epon 812 solution for 2 h, in 100% Epon twice for 1 h each, then the monolayer cells were covered with a layer of Epon and polymerized in an oven at 60 °C for 48 h. At the end of the polymerization the plastic was separated from the Epon block mechanically breaking the plastic, leaving the cell monolayer facing up on the resin block. A portion of the specimen was glued on top of an epon block and mounted on a Leica Ultracut UCT ultramicrotome. Ultrathin (70–90 nm) sections were then collected on copper grids and stained with uranyl acetate and lead citrate before being imaged with TEM. Mitochondria were quantified using Fiji software as the percentage of cytosolic area occupancy.

### Cut&Tag

We performed a Cleavage Under Targets and Tagmentation (CUT&Tag) assay (Kaya-Okur et al, 2019) following the protocol: CUT&Tag-direct for whole cells with CUTAC V.5 (https://www.protocols.io). We used 100,000 cells for each experimental condition. A primary antibody (CREB - D76D11 – rabbit mAb #4820, 1:50 dilution) incubation was performed overnight. A secondary antibody (Guinea Pig anti-Rabbit IgG, ABIN101961, antibodies-online.com) incubation was performed for 2 h. As Tn5 enzyme CUTANA™ pAG-Tn5 (15–1017, Epicypher^®^, 1:20 dilution) was used for an incubation time of 2 h. Read tags were aligned to reference genome (hg38) using chromap (Zhang et al, 2021) [-q 0 --preset atac], peaks were identified using MACS3 (Zhang et al, 2008) with default parameters. We used bedtools (Quinlan and Hall, 2010) to compute a reference peakset by merging all individual peaks and to quantify peak coverage. We compared scr/sh using edgeR (Chen et al, 2025). Coverage tracks were obtained using bedtools.

### Myoblast differentiation

C2C12 cell line was cultured in a 6-well plate (100,000–150,000 cells/w) for 24 h, until they reached confluency. Then growth medium was replaced with differentiation medium (DMEM high glucose, 2% HS, and 1%P/S). Cells were then kept in culture, and medium was replaced every 48 h. After 72/96 h the myotube formation was assessed by immunofluorescence.

### Satellite cells isolation by FACS

Tibialis anterior (TA) and gastrocnemius (GA) muscles from mice were enzymatically dissociated in PBS containing 2 mg/mL Collagenase A (Roche, 10 103 586 001), 2.4 U/mL Dispase II (Roche, 04 942 078 001), 10 ng/mL DNase I (Sigma-Aldrich, 11 284 932 001), 0.4 mM CaCl₂, and 5 mM MgCl₂ for 30 min at 37 °C. The resulting cell suspension was filtered through a 50 μm sterile syringe Filcon (BD, 340602) and incubated for 30 min with the following antibodies: CD45 (Invitrogen, 48-0451-82), CD31 (Invitrogen, 48-0311-82), TER119 (Invitrogen, 48-5921-82), Sca1 (Invitrogen, 11-5981-82), and Itga7 (AbLab, R2F2). Satellite cells were isolated as TER119⁻CD45⁻CD31⁻Sca1⁻Itga7⁺ cells.

### Satellite cells culture

Freshly isolated SCs were resuspended in growth medium (GM) consisting of DMEM (+ Pyruvate), 20% FBS, 10% horse serum (HS), 1% penicillin/streptomycin, and 1% chicken embryo extract (CEE). The cells were then plated in gelatin-coated 24-well plates. Myogenic differentiation was induced by switching to differentiation medium (DM), composed of DMEM (+ Pyruvate) and 2% HS. Myotube formation was evaluated via immunofluorescence. Cultured SCs were fixed in 4% PFA for 10 min, then permeabilized with 0.1% Triton X-100 for 15 min at room temperature. Blocking was performed for 1 h using 4% BSA in PBS. Cells were incubated overnight at 4 °C with the primary anti-Myosin antibody (MF20) in 4% BSA, followed by incubation with the secondary antibody conjugated to Alexa Fluor 488. Nuclei were stained with DAPI in PBS for 10 min.

### Statistical analysis

Statistical analysis was performed on experiments with 3 to 5 biological replicates. Differential results were tested with a statistical analysis. Significant differences were quantified by applying Paired or Unpaired Student’s t-test, and one-way or two-way ANOVA. A normalization of all samples to 1 was adopted when the experiment entailed paired samples. An equalizer or raw measurement were plotted in the case of unpaired samples. One sample t test was applied each time a normalization to 1 was necessary. Each statistical test is specified in figure captions. *p < 0.05, **p < 0.01, ***p < 0.001, ****p < 0.0001, ns = not significative. Statistics and plots were generated with using the GraphPad Prism version 9.5.0 to 11.0.2 or JASP 0.19.3.

### RNAseq

For U-2 OS cells, 6 samples (3xshSCR, 3xsh*KDM6A*) were obtained for RNA-Seq using TrueSeq mRNA Illumina Unstranded in NextSeq 550 to obtain 30 M clusters of single end 75 bp reads reversely stranded. Reads were aligned to the human genome (hg38) using STAR (Dobin et al, 2013) (version: STAR_2.5.3a) and gene-level counts were obtained by featureCounts using the gene annotation from GeneCode (version 31). The differentially expressed genes (DEGs) between sh*KDM6A* and shSCR were obtained using linear models through the use of the R/Bioconductor package limma (Ritchie et al, 2015). Data were ranked by log_2_FC and exported as tab delimited text (.txt). The gene list was loaded in Gene Set Enrichment Analysis (GSEA (Subramanian et al, 2005)) 4.3.1 software. A GSEA pre-ranked analysis was computed against 10,000 control permutations. H.all.v2022.1.Hs.symbols.gmt and c5.go.bp.v2022.1.Hs.symbols.gmt were selected as gene set database. Enriched pathways were selected as FDR q-value < 0.05 and displayed in a bar-chart showing normalized enrich score (NES) on x axis.

Transcriptomic profiles of 8 ARP-1 samples (4xWT, 4xKO) were obtained by RNA-Seq using mRNA Stranded TruSeq Illumina library preparation protocols (Illumina, Inc) to obtain 30 M clusters of single-end 100 bp reads through Illumina NovaSeq 6000 SP Reagent Kit (100 cycles). Reads were aligned to the human genome (hg38) using STAR (Dobin et al, 2013) (version: STAR_2.5.3a) and gene-level counts were obtained by featureCounts using the gene annotation from GeneCode (version 31). DEGs between KO and WT cells ARP-1 cells were obtained by a paired analysis using linear models through the use of the R/Bioconductor package limma (Ritchie et al, 2015). Data were ranked by log_2_FC and exported as tab delimited text (.txt). The gene list was loaded in Gene Set Enrichment Analysis (GSEA (Subramanian et al, 2005)) 4.3.1 software. A GSEA pre-ranked analysis was computed against 10,000 control permutations.

Sorted SCs RNA was sequenced through SMART-seq (6 biological replicates for each condition divided into 2 batches, one WT untreated replicate of the first batch was discarded due to lowQC and one KO treated of the second was discarded as an outlier) in Illumina NovaSeq 6000. Adapters and low-quality sequences were trimmed using Trimmomatic (Bolger et al, 2014). Reads were aligned to the mouse genome (MM10) using STAR (Dobin et al, 2013) (version: STAR_2.5.3a) and gene-level counts were obtained by featureCounts using the gene annotation from GeneCode (version 31). DEGs between KO and WT SCs and between not treated and CDX treated conditions were obtained using linear models through the use of the R/Bioconductor package limma (Ritchie et al, 2015). Data were ranked by log_2_FC and exported as tab delimited text (.txt). The gene list was loaded in Gene Set Enrichment Analysis (GSEA (Subramanian et al, 2005)) 4.3.1 software. A GSEA pre-ranked analysis was computed against 10,000 control permutations. H.all.v2022.1.Hs.symbols.gmt was selected as gene set database. Enriched pathways were selected as FDR q-value < 0.05 and displayed in a bar-chart showing normalized enrich score (NES) on x axis.

### TCGA Pan Cancer Atlas data analysis

Gene expression data from TCGA datasets were downloaded from the cBioPortal data hub (https://www.cbioportal.org) for the selected datasets from the pan-cancer atlas (Hoadley et al, 2018) of the TCGA, in the form of mRNA RNA-Seq RSEM analyzed reads. For each dataset, genes with zero counts were removed. All the patients analyzed are female in order to avoid potential UTY compensation. The limma-voom approach (Ritchie et al, 2015) was run to normalize RSEM values and KDM6A higher (upper quartile) or lower (lower quartile) expressors were selected. For GSEA analysis a complete table of voom normalized gene expression for all higher and lower expressors was input to the GSEA algorithm using both Hallmarks (H.all.v2022.1.Hs.symbols.gmt) and Gene Ontology Biological Process (GOBP) (c5.go.bp.v2022.1.Hs.symbols.gmt) to obtain pathways enriched. Heatmaps of Normalized Enrichment score were constructed for Upregulated (in lower expressors) or Downregulated (in higher expressors) Hallmarks and GOBP that showed a canonical p-value of at least 0.05 in at least one dataset. An averaged NES was calculated and the top9 Hallmarks and GOBP pathways were selected.

### MitoMass TCGA data analysis

mRNA expression and clinical data from TCGA PanCancer cohort were retrieved via the cBioPortal database. Samples were queried based on *KDM6A* expression, and only female patient data were retained for further analysis. The dataset was then stratified by *KDM6A* expression levels, with samples divided into upper and lower quartiles to define *KDM6A*-high and *KDM6A*-low groups, respectively. These filtered data were subsequently matched with mtDNA information obtained from published data (Reznik et al, 2016). The final dataset underwent normality test. Statistical comparisons between groups were performed using the two-tailed Mann–Whitney U test, and corresponding *P* value was calculated.

### MMRF patients’ data analysis

We downloaded expression data from MMRF^®^ (IA18) as gene quantification in transcripts per million reads (tpm). We obtained the segmentation data for the same dataset and calculated the average segmentation value for all female patients over *KDM6A* locus. For patients with matched data between two modalities we built a linear model with interaction including sex as binary covariate and KDM6A z-score as continuous covariate using limma (Ritchie et al, 2015).

### Spatial transcriptomic analysis

The dataset used was licensed under the Creative Commons Attribution 4.0 International license (available at: https://creativecommons.org/licenses/by/4.0/). Data were retrieved from Visium HD Spatial Gene Expression Library, Mouse Embryo (FFPE), HD Spatial Gene Expression dataset analyzed using Space Ranger 4.0.1, 10x Genomics. Analysis was performed using 10x Genomics Loupe Browser (v8.1.2). A GSEA pre-ranked analysis was computed against 1000 control permutations, m8.all.v2025.1.Mm.symbols.gmt was selected as gene set database. Enriched pathways were selected as FDR q-value < 0.05 and displayed in a bar-chart showing normalized enrich score (NES) on x axis.

### Mouse embryo single cell data analysis

Mouse embryo single cell data (Qiu et al, 2024) were downloaded from cellxgene data portal. For each cell type (“author_major_cell_cluster”) and developmental time (“author_day”) we built a co-expression network as follows: genes found in less than 5 cells were removed from the data subset, genes were normalized using pf_log1p_pf procedure (preprint: Booeshaghi et al, 2022), pairwise proportionality correlation coefficient ρ (Lovell et al, 2015) were computed. We computed the difference between coefficients for the *Kdm6a/Ccdc3* and *Kdm6a/Traf3ip3* in each tissue/cell type, the resulting vectors were clustered to generate the heatmap shown in Figs. 7 and EV7.

## Supplementary information

Table EV1

Peer Review File

Source data Fig. 1

Source data Fig. 2

Source data Fig. 3

Source data Fig. 4

Source data Fig. 5

Source data Fig. 6

Source data Fig. 7

Expanded View Figures

## Data Availability

RNAseq and Cut&Tag data reported in this manuscript have been deposited in the publicly available repository ArrayExpress (https://www.ebi.ac.uk/biostudies/arrayexpress): E-MTAB-15701: RNASeq_U2OS, E-MTAB-15716: RNASeq_ARP1, E-MTAB-15714: RNASeq_SCs, E-MTAB-15717: Cut&Tag. The source data of this paper are collected in the following database record: biostudies:S-SCDT-10_1038-S44318-026-00891-0.
