# Supplementary material for: KDM6A loss enhances oxidative phosphorylation uncovering tissue-level convergent evolution
Source: EMBO J. 2026 Sep 4;45(19):6847–82. doi: 10.1038/s44318-026-00891-0 (PMC13631268; doi:10.1038/s44318-026-00891-0)
Supplement: Supplementary file 1 [file 44318_2026_891_MOESM10_ESM.pdf]

Expanded View Figures

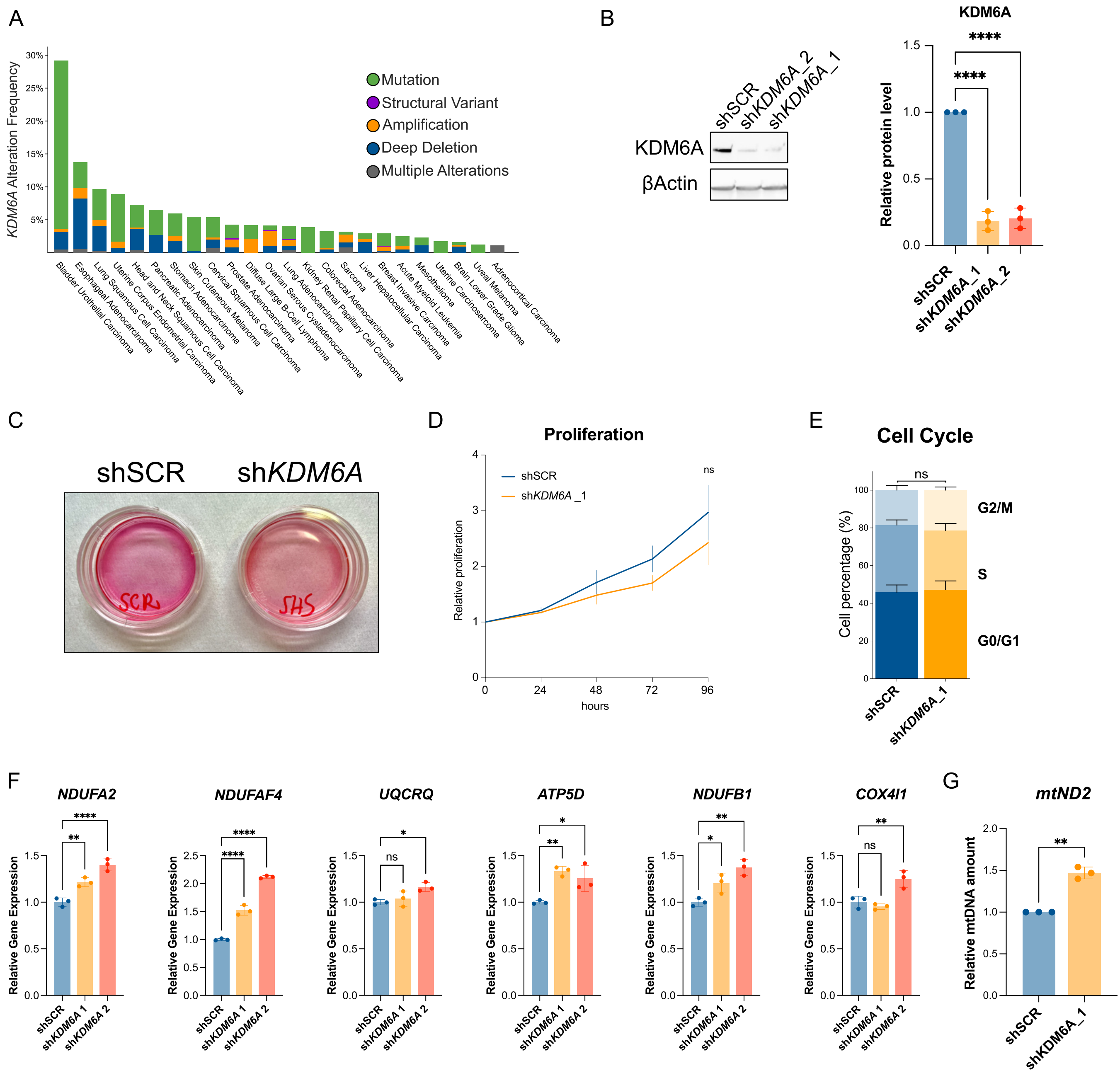

Figure EV1. The loss od KDM6A increases medium acidification without perturbing proliferation and cell cycle but increasing ETC genes expression.

(**A**) *KDM6A* alteration frequency from several patient datasets from TCGA, PanCancer Atlas (minimum frequency > 1%). Bar chart generated in cBioPortal browser. (**B**) One representative out of three western blot of KDM6A protein level on shSCR (scrambled short hairpin RNA) and sh*KDM6A* conditions. Normalization on βActin and shSCR. Statistical significance was assessed by applying one-way ANOVA test. Mean +/− SD, $n = 3$ (****$p = 0.0000032$, $p = 0.0000037$). (**C**) Representative images of cell culture media acidification of U-2 OS shSCR compared to U-2 OS *KDM6A* KD. (**D**) Relative proliferation at 24, 48, 72, and 96 h of shSCR and *KDM6A* KD cells of three independent biological replicates. Mean +/− SEM. Unpaired t-test (ns $p = 0.4352$). (**E**) Cell cycle phases, percentage of shSCR and sh*KDM6A* cells. Data are represented as mean +/− SD of four independent biological replicates. Statistical significance was assessed by applying two-way ANOVA test (ns $p = 0.533$). (**F**) Gene expression of ETC genes on shSCR and sh*KDM6A* conditions evaluated by RT-qPCR. Data are represented as mean +/− SD of three independent biological replicates. Statistical significance was assessed by applying one-way ANOVA test (*NDUFA2* **$p = 0.002751$, ****$p = 0.000098$; *NDUFAF4* ****$p = 0.0000212$, ****$p = 0.0000002$; *UQCRQ* ns $p = 0.4587$, *$p = 0.0135$; *ATP5D* **$p = 0.0034$, *$p = 0.0113$; *NDUFB1* *$p = 0.0218$, **$p = 0.0013$; *COX4I1* ns $p = 0.4038$, **$p = 0.004$). (**G**) Abundance of mtDNA (*mtND2*) assessed by qPCR. Data are normalized to *NBPF9* and to shSCR condition and represented as mean +/− SD of three independent biological replicates. Significant differences were quantified by one sample t-test (**$p = 0.0073$).

© The Author(s)

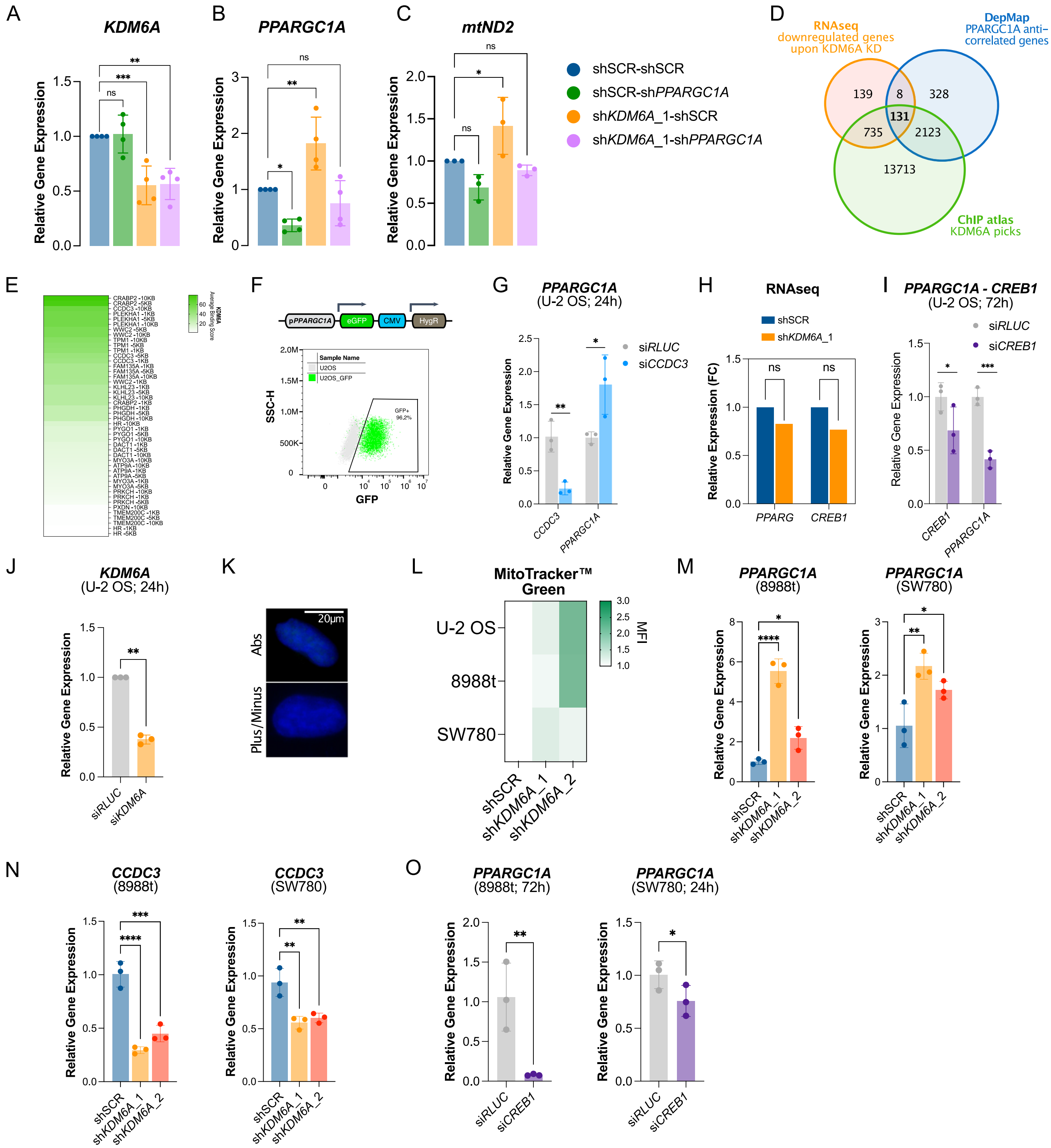
A
KDM6A
B
PPARGC1A
C
mtND2
shSCR-shSCR
shSCR-shPPARGC1A
shKDM6A_1-shSCR
shKDM6A_1-shPPARGC1A
D
RNAseq downregulated genes upon KDM6A KD
DepMap PPARGC1A anti-correlated genes
ChIP atlas KDM6A picks
E
F
G
PPARGC1A (U-2 OS; 24h)
siRLUC
siCCDC3
H
RNAseq
shSCR
shKDM6A_1
I
PPARGC1A - CREB1 (U-2 OS; 72h)
siRLUC
siCREB1
J
KDM6A (U-2 OS; 24h)
K
L
MitoTracker™ Green
M
PPARGC1A (8988t)
PPARGC1A (SW780)
N
CCDC3 (8988t)
CCDC3 (SW780)
O
PPARGC1A (8988t; 72h)
PPARGC1A (SW780; 24h)
Relative Gene Expression

Figure EV2. CCDC3 loss induces *PPARGC1A* expression mirroring the effect of *KDM6A* KD in PaTu8988t and SW780 as observed in U-2 OS cells.

(**A**) Gene expression levels of *KDM6A* and (**B**) *PPARGC1A* in U-2 OS with double shRNAs assessed by RT-qPCR. Data are normalized to *TBP* and to shSCR-shSCR condition and represented as mean +/− SD of four independent biological replicates. Statistical significance was assessed by applying one-way ANOVA test (*KDM6A*: ns $p = 0.8367$, ***$p = 0.0008$, **$p = 0.001$; *PPARGC1A*: *$p = 0.0141$, **$p = 0.0031$, ns $p = 0.2954$). (**C**) mtDNA quantification (*mtND2*) was measured through qPCR. Data are normalized to *NBPF9* and to shSCR-shSCR condition and represented as mean +/− SD of three independent biological replicates. Statistical significance was assessed by applying one-way ANOVA test (ns $p = 0.0761$, *$p = 0.0259$, ns = 0.4932). (**D**) Venn diagram of shared significant genes between RNAseq, DepMap, and ChIPAtlas. (**E**) Genes ranked by *KDM6A* average binding score. (**F**) GFP-positive cells after transduction with *PPARGC1A* expression reporter. (**G**) Gene expression levels measured by RT-qPCR of *PPARGC1A* and *CCDC3* in U-2 OS after 24 h silencing with siRNA. Data are normalized to *TBP* and to siRLUC control and represented as mean +/− SD of three independent biological replicates. Statistical significance was assessed by applying multiple unpaired t-test (**$p = 0.0058$, *$p = 0.0386$). (**H**) Gene expression of *PPARG* and *CREB1* from RNAseq ($n = 3$, limma, ns adj. $p = 0.51$, ns adj. $p = 0.14$). (**I**) Gene expression levels analyzed by RT-qPCR of *CREB1* and *PPARGC1A* in U-2 OS after 72 h silencing with siRNA. Data are normalized to *TBP* and to siRLUC control and represented as mean +/− SD of three independent biological replicates. Statistical significance was assessed by applying one-tailed unpaired t-test (*$p = 0.05$, ***$p = 0.0004$). (**J**) Expression levels of *KDM6A* measured by RT-qPCR in U-2 OS after 24 h silencing with siRNA. Mean +/− SD, $n = 3$. Statistical significance was assessed by applying one sample t-test (**$p = 0.0017$). (**K**) PLA negative controls. Abs = only antibodies. Plus/Minus = only probes. (**L**) Heatmap showing the median fluorescence intensity (MFI) of MitoTracker™ Green in U-2 OS, PaTu8988t, and SW780 cell lines. (**M**) Expression levels assessed by RT-qPCR of *PPARGC1A* and (**N**) *CCDC3*, on PaTu8988t and SW780 cell lines silenced with two different shRNAs targeting *KDM6A*. Data are normalized to *TBP* and to shSCR condition and represented as mean +/− SD of three independent biological replicates. One-way ANOVA test was performed (*PPARGC1A*: (8988t) ****$p = 0.000028$, *$p = 0.024629$; (SW780) **$p = 0.0033$, *$p = 0.0304$. *CCDC3*: (8988t) ****$p = 0.000049$, ***$p = 0.000198$; (SW780) **$p = 0.0018$, **$p = 0.0034$). (**O**) Gene expression levels measured by RT-qPCR of *CREB1* in PaTu8988t and SW780 cell line after *KDM6A* silencing. Data are normalized to *TBP* and to control condition (siRLUC) and represented as mean +/− SD of three independent biological replicates. Data were collected at 72 h and 24 h, respectively. Statistical significance was assessed by one-tailed Unpaired t-test (8988t: **$p = 0.0083$; SW780 *$p = 0.0476$).

 © The Author(s)

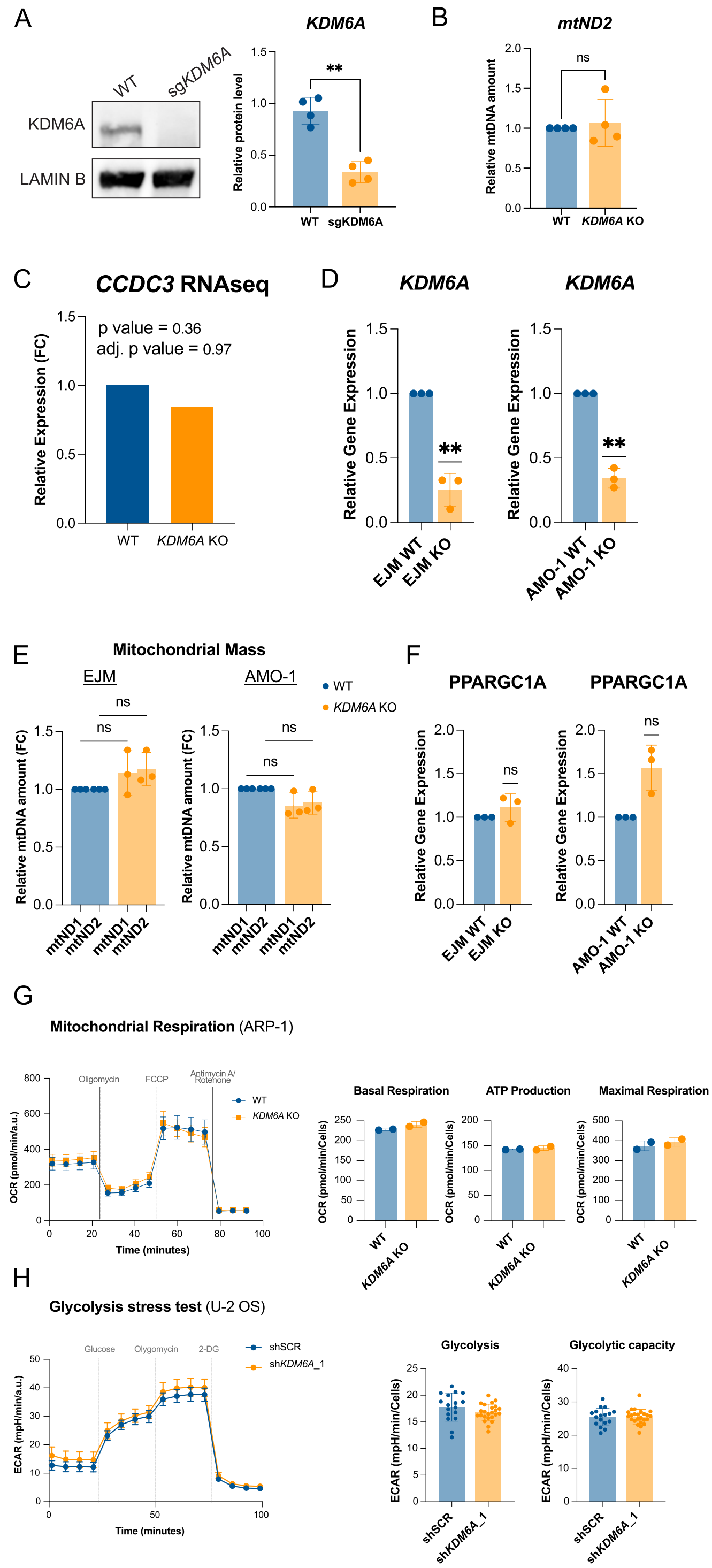
A
WT
sgKDM6A
KDM6A
LAMIN B
KDM6A
Relative protein level
**
WT
sgKDM6A
B
mtND2
Relative mtDNA amount
ns
WT
KDM6A KO
C
CCDC3 RNAseq
Relative Expression (FC)
p value = 0.36
adj. p value = 0.97
WT
KDM6A KO
D
KDM6A
KDM6A
Relative Gene Expression
**
EJM WT
EJM KO
AMO-1 WT
AMO-1 KO
E
Mitochondrial Mass
EJM
AMO-1
WT
KDM6A KO
Relative mtDNA amount (FC)
ns
mtND1
mtND2
F
PPARGC1A
PPARGC1A
Relative Gene Expression
ns
EJM WT
EJM KO
AMO-1 WT
AMO-1 KO
G
Mitochondrial Respiration (ARP-1)
Oligomycin
FCCP
Antimycin A/ Rotenone
OCR (pmol/min/a.u.)
Time (minutes)
WT
KDM6A KO
Basal Respiration
ATP Production
Maximal Respiration
OCR (pmol/min/Cells)
H
Glycolysis stress test (U-2 OS)
Glucose
Olygomycin
2-DG
ECAR (mpH/min/a.u.)
Time (minutes)
shSCR
shKDM6A_1
Glycolysis
Glycolytic capacity
ECAR (mpH/min/Cells)

Figure EV3. MM cell lines do not exhibit increased mitochondrial mass, *PPARGC1A* expression or OXPHOS upon *KDM6A* KO.

(**A**) One representative out of four western blot and quantification of KDM6A after CRISPR Cas9 KO in ARP-1 cells. Protein levels of KDM6A were measured in four independent biological replicates and normalized to LAMIN B. Mean +/− SD. Two-tailed Paired Student's t-test (**$p = 0.0049$). (**B**) Abundance of mtDNA (*mtND2*) measured by qPCR in WT and KO ARP-1 cell line. Data are normalized to *NBPF9* and to WT condition and represented as mean +/− SD of four independent biological replicates. Statistical significance was assessed by one sample t-test (ns $p = 0.6783$). (**C**) *CCDC3* expression from RNAseq in WT and *KDM6A* KO ARP-1 cells ($n = 4$, limma). (**D**) Gene expression of *KDM6A* in WT and KO EJM and AMO-1 cell lines analyzed by RT-qPCR. Data are normalized to *TBP* and to WT and represented as mean +/− SD of three independent biological replicates. Statistical significance was assessed by one sample t-test (**$p = 0.0097$, **$p = 0.0045$). (**E**) Abundance of mtDNA (*mtND1* and *mtND2*) in WT and KO EJM and AMO-1 cell lines measured by qPCR. Data are normalized to *NBPF9* and to WT condition and represented as mean +/− SD of three independent biological replicates. Statistical significance was assessed by multiple unpaired t-test (EJM: ns $p = 0.276$, ns $p = 0.0978$; AMO-1: ns $p = 0.083$, ns $p = 0.1046$). (**F**) Expression of *PPARGC1A* in WT and *KDM6A* KO EJM and AMO-1 cell lines analyzed by RT-qPCR. Data are normalized to *TBP* and to WT and represented as mean +/− SD of three independent biological replicates. Statistical significance was assessed by one sample t-test (ns $p = 0.3467$, ns $p = 0.0648$). (**G**) One representative analysis out of two independent biological replicates of OCR measurements. Bar plots of basal respiration, ATP production, and Maximal Respiration of two independent biological replicates. Mean +/− SD. (**H**) Analysis of ECAR measurements and bar plots of glycolysis and glycolytic capacity on U-2 OS cell line. Mean +/− SD ($n = 17$, $n = 24$; $n = 17$, $n = 24$).

 © The Author(s)

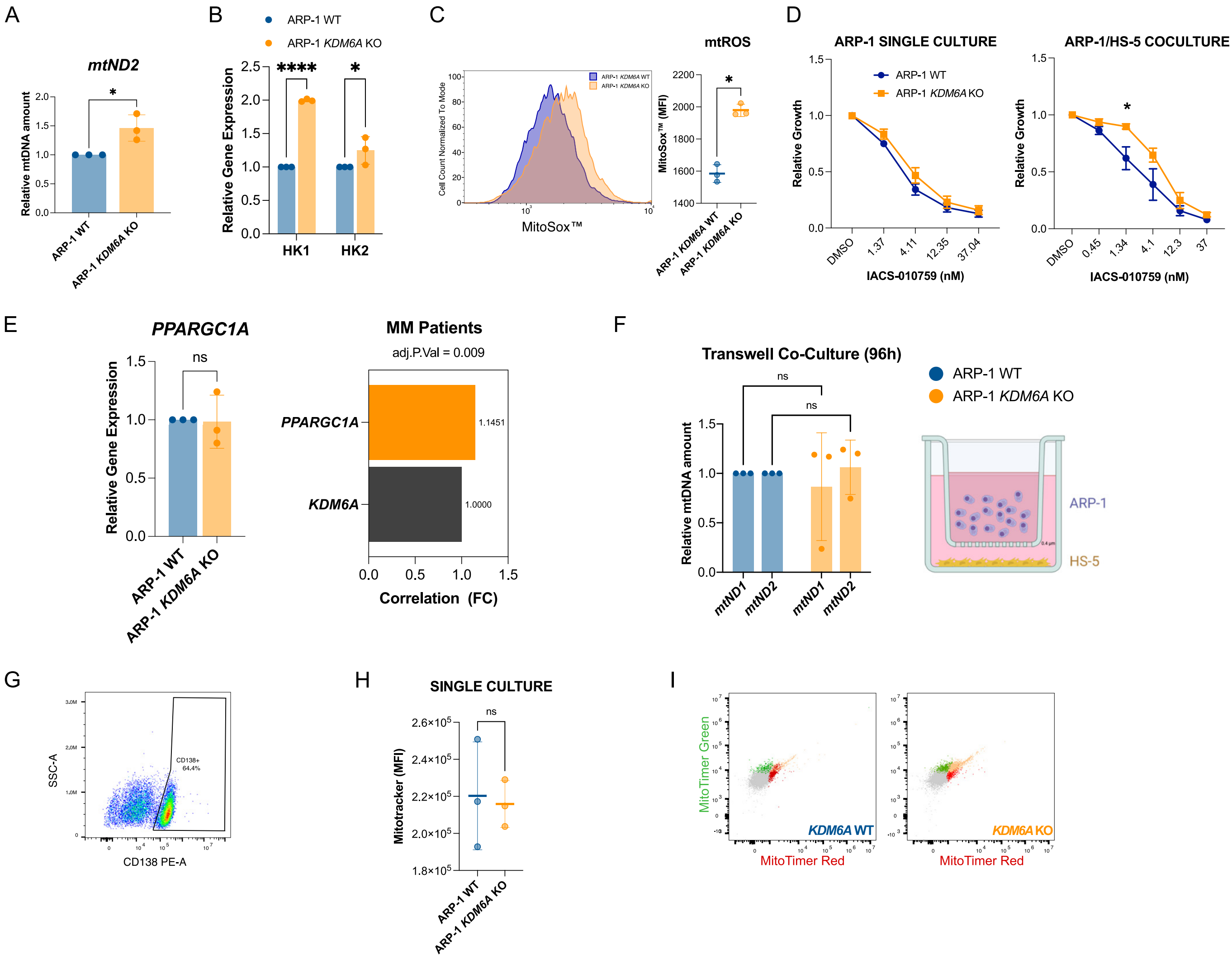

Figure EV4. KDM6A KO in MM cells increases glycolysis, ROS production and resistance to OXPHOS inhibitors upon coculture as a result of cell-to-cell contact.

(**A**) Abundance of mtDNA (*mtND2*) measured by qPCR after coculture. Data are normalized to *NBPF9* and to WT condition and represented as mean +/− SD of three independent biological replicates. Statistical significance was assessed by one sample t-test (*$p = 0.036$). (**B**) Gene expression of *HK1* and *HK2* in ARP-1 cells following co-culture with HS-5. RT-qPCR data are normalized to *TBP* and to WT and represented as mean +/− SD of three independent biological replicates. Statistical significance was assessed by two-way ANOVA (****$p = 0.00002$, *$p = 0.019342$). (**C**) One representative histogram and quantification of MitoSox™ staining of ARP-1 cells following co-culture with HS-5. MFI measurements from three independent biological replicates. Mean +/− SD. Statistical significance was assessed by applying paired Student's t-test (*$p = 0.0114$). (**D**) ARP-1 single culture and coculture treated with IACS-010759 at different concentrations. Mean +/− SEM ($n = 3$). Unpaired t test (*$p = 0.0265$). (**E**) On the left, gene expression measured by RT-qPCR of *PPARGC1A* in ARP-1 cells following co-culture with HS-5. Data are normalized to *TBP* and to WT and represented as mean +/− SD of three independent biological replicates. Statistical significance was assessed by one sample t-test (ns $p = 0.9128$). On the right, linear regression of *PPARGC1A* and *KDM6A* in MM patients. Data shown as fold change of the correlation. (**F**) Abundance of mtDNA (*mtND1*, *mtND2*) analyzed by qPCR in ARP1 cells when cocultured with HS-5 in a transwell system. Data are normalized to *NBPF9* and to WT condition and represented as mean +/− SD of three independent biological replicates. Statistical significance was assessed by two-tailed Unpaired Student's t-test (ns $p = 0.691167$, ns $p = 0.717478$). Schematic representation of transwell (0.4 µm) coculture. (**G**) Gated CD138 positive cells after ARP-1/HS-5 coculture. (**H**) MFI of MitoTracker™ green stained ARP-1 *KDM6A* WT and KO cell line in single culture. Mean +/− SD, $n = 3$. One-tailed paired t test (ns $p = 0.343$). (**I**) Dot plot of Mitotimer green and red positive *KDM6A* WT and KO ARP-1 cells after HS-5 coculture.

© The Author(s)

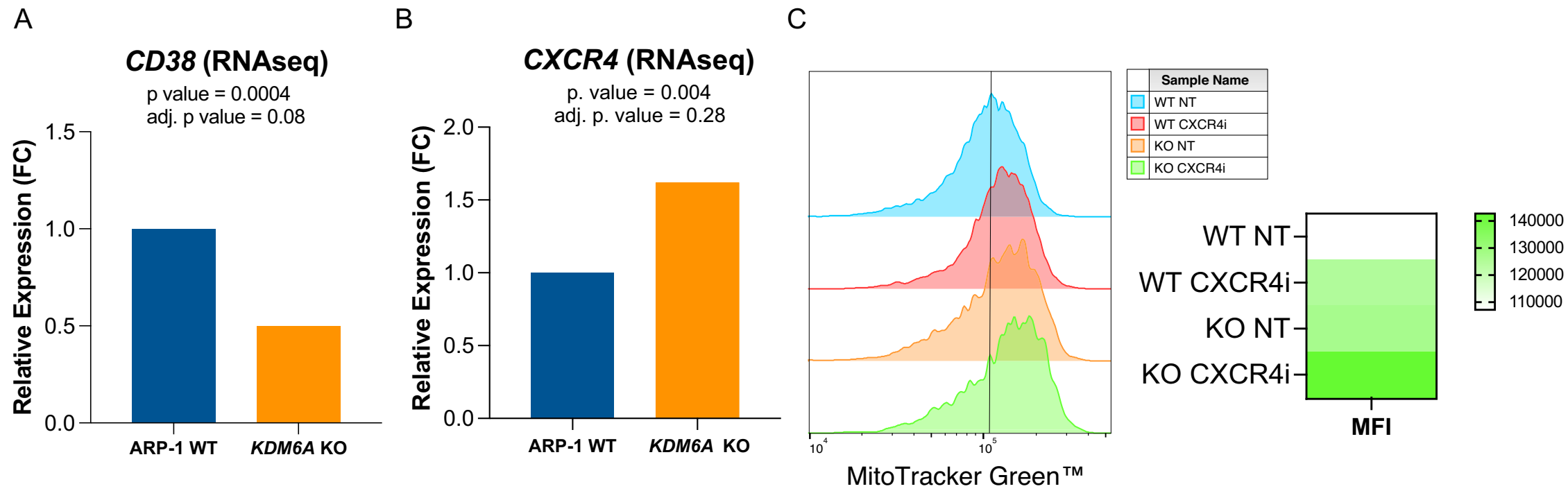

Figure EV5. Mitochondrial trafficking regulators *CD38* and *CXCR4* are not induced by KDM6A loss.

(**A**) Expression of *CD38* from RNAseq in ARP-1 cells. (**B**) *CXCR4* expression from RNAseq in WT and *KDM6A* KO ARP-1 cells. (**C**) MitoTracker™ green fluorescence measured by FACS in CXCR4i treated HS-5 cocultured ARP-1 cells.

 © The Author(s)

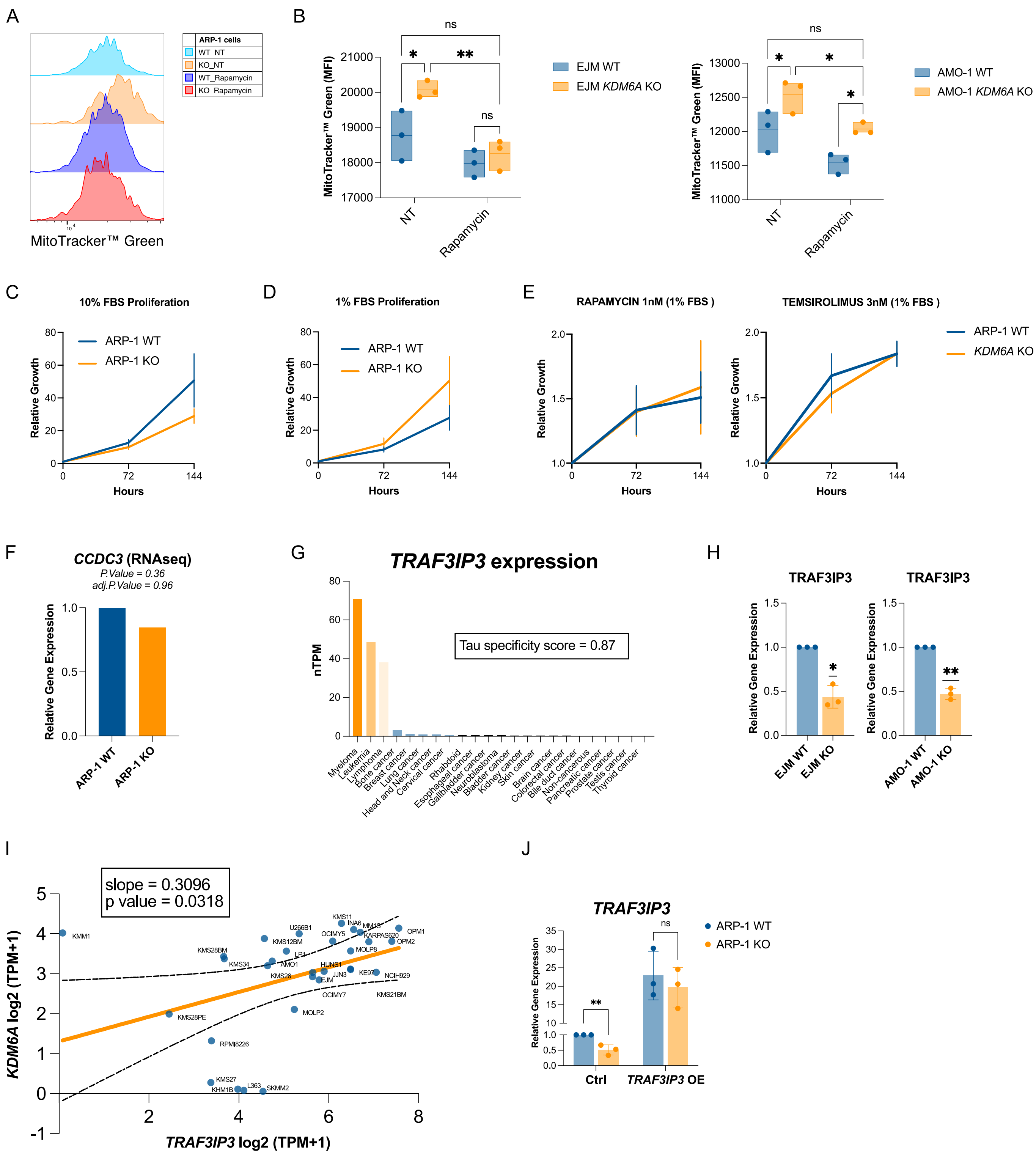
A
ARP-1 cells
WT_NT
KO_NT
WT_Rapamycin
KO_Rapamycin
MitoTracker™ Green
B
MitoTracker™ Green (MFI)
EJM WT
EJM KDM6A KO
AMO-1 WT
AMO-1 KDM6A KO
NT
Rapamycin
ns
C
10% FBS Proliferation
D
1% FBS Proliferation
E
RAPAMYCIN 1nM (1% FBS)
TEMSIROLIMUS 3nM (1% FBS)
ARP-1 WT
ARP-1 KO
KDM6A KO
Relative Growth
Hours
F
CCDC3 (RNAseq)
P.Value = 0.36
adj.P.Value = 0.96
Relative Gene Expression
G
TRAF3IP3 expression
Tau specificity score = 0.87
nTPM
Myeloma
Leukemia
Lymphoma
Bone cancer
Breast cancer
Head and Neck cancer
Lung cancer
Cervical cancer
Rhabdoid
Esophageal cancer
Gallbladder cancer
Neuroblastoma
Bladder cancer
Kidney cancer
Skin cancer
Brain cancer
Colorectal cancer
Bile duct cancer
Non-cancerous
Pancreatic cancer
Prostate cancer
Testis cancer
Thyroid cancer
H
TRAF3IP3
EJM WT
EJM KO
AMO-1 WT
AMO-1 KO
I
slope = 0.3096
p value = 0.0318
KDM6A log2 (TPM+1)
TRAF3IP3 log2 (TPM+1)
J
TRAF3IP3
Ctrl
TRAF3IP3 OE

◀ **Figure EV6. *KDM6A* KO in MM cell lines downregulates the mTOR inhibitor TRAF3IP3 to induce proliferation and mitochondrial trafficking.**

(**A**) One representative MFI measurement by FACS of MitoTracker™ Green in ARP-1 cells upon *KDM6A* KO and Rapamycin 50 nM treatment. (**B**) MFI of MitoTracker™ measured on HS-5 cocultured EJM and AMO-1 cells treated with Rapamycin 50 nM. Data are normalized to WT and represented as mean +/− SD of three independent biological replicates. Significant differences were quantified by applying two-way ANOVA test (EJM: $*p = 0.0103$, ns $p = 0.2228$, $**p = 0.0016$, ns $p = 4926$; AMO-1: $*p = 0.018$, ns $p = 0.9458$, $*p = 0.0201$, $*p = 0225$). (EJM) NT-WT: Min = 18,053, Median = 18,784, Max=19,481; NT-KO: Min = 19,873, Median = 20,005, Max = 20,340; Rapa-WT: Min = 17,583, Median = 17,994, Max = 18,354; Rapa-KO: Min = 17,758, Median = 18,415, Max= 18,599. (AMO-1) NT-WT: Min = 11,690, Median = 12,092, Max=12290; NT-KO: Min = 12,259, Median = 12,664, Max = 12,712; Rapa-WT: Min = 11,373, Median = 11,588, Max = 11,661; Rapa-KO: Min = 11,986, Median = 11,986, Max = 12,137. (**C**) Proliferation evaluation of WT and KO ARP-1 cell line in 10% FBS growth medium. Mean +/− SEM ($n = 3$). (**D**) Proliferation evaluation of WT and KO ARP-1 cell line in 1% FBS growth medium. Mean +/− SEM ($n = 3$). (**E**) Proliferation evaluation of WT and KO ARP-1 cell line in 1% FBS growth medium during treatment with Rapamycin 1 nM and Temsirolimus 3 nM. Mean +/− SEM ($n = 3$). (**F**) Differential gene expression of *CCDC3* from RNAseq in ARP-1 cells upon *KDM6A* KO ($n = 4$, limma). (**G**) Normalized Transcript per Million (nTPM) values of cancer cell lines categories ranked for *TRAF3IP3* expression levels. Tau specificity score is indicated. (**H**) Gene expression levels of *TRAF3IP3* were measured through RT-qPCR. Data are normalized to *TBP* and to WT and represented as mean +/− SD of three independent biological replicates. Significant differences were quantified by one sample t-test ($*p = 0.0167$, $**p = 0.0049$). (**I**) Linear regression of *KDM6A* and *TRAF3IP3* expression across different MM cell lines from DepMap. (**J**) Gene expression analyzed by RT-qPCR of *TRAF3IP3* in control and *TRAF3IP3* overexpressed ARP-1 cells. Data are normalized to *TBP* and to WT and represented as mean +/− SD of three independent biological replicates. Statistical significance was assessed by applying multiple unpaired t tests ($**p = 0.0074$, ns $p = 0.5532$).

 © The Author(s)

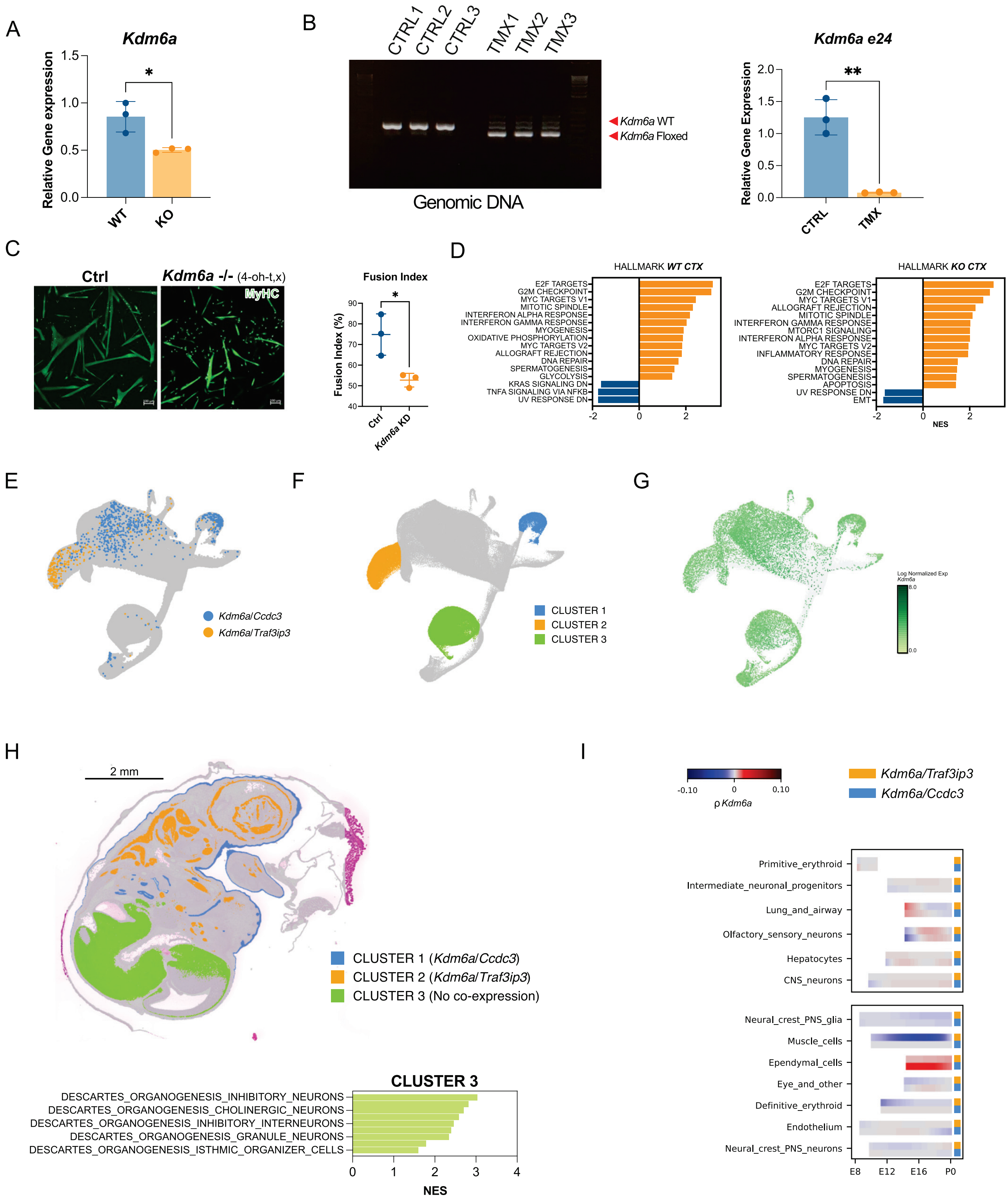
A
Kdm6a
Relative Gene expression
1.5
1.0
0.5
0.0
*
WT
KO
B
CTRL1
CTRL2
CTRL3
TMX1
TMX2
TMX3
Kdm6a WT
Kdm6a Floxed
Genomic DNA
Kdm6a e24
Relative Gene Expression
2.0
1.5
1.0
0.5
0.0
**
CTRL
TMX
C
Ctrl
Kdm6a -/- (4-oh-t,x)
MyHC
Fusion Index
Fusion Index (%)
90
80
70
60
50
40
*
Ctrl
Kdm6a KD
D
HALLMARK WT CTX
E2F TARGETS
G2M CHECKPOINT
MYC TARGETS V1
MITOTIC SPINDLE
INTERFERON ALPHA RESPONSE
INTERFERON GAMMA RESPONSE
MYOGENESIS
OXIDATIVE PHOSPHORYLATION
MYC TARGETS V2
ALLOGRAFT REJECTION
DNA REPAIR
SPERMATOGENESIS
GLYCOLYSIS
KRAS SIGNALING DN
TNFA SIGNALING VIA NFKB
UV RESPONSE DN
-2
0
2
HALLMARK KO CTX
E2F TARGETS
G2M CHECKPOINT
MYC TARGETS V1
ALLOGRAFT REJECTION
MITOTIC SPINDLE
INTERFERON GAMMA RESPONSE
MTORC1 SIGNALING
INTERFERON ALPHA RESPONSE
MYC TARGETS V2
INFLAMMATORY RESPONSE
DNA REPAIR
MYOGENESIS
SPERMATOGENESIS
APOPTOSIS
UV RESPONSE DN
EMT
-2
0
2
NES
E
Kdm6a/Ccdc3
Kdm6a/Traf3ip3
F
CLUSTER 1
CLUSTER 2
CLUSTER 3
G
H
2 mm
CLUSTER 1 (Kdm6a/Ccdc3)
CLUSTER 2 (Kdm6a/Traf3ip3)
CLUSTER 3 (No co-expression)
CLUSTER 3
DESCARTES_ORGANOGENESIS_INHIBITORY_NEURONS
DESCARTES_ORGANOGENESIS_CHOLINERGIC_NEURONS
DESCARTES_ORGANOGENESIS_INHIBITORY_INTERNEURONS
DESCARTES_ORGANOGENESIS_GRANULE_NEURONS
DESCARTES_ORGANOGENESIS_ISTHMIC_ORGANIZER_CELLS
0
1
2
3
4
NES
I
-0.10
0
0.10
ρ Kdm6a
Kdm6a/Traf3ip3
Kdm6a/Ccdc3
Primitive_erythroid
Intermediate_neuronal_progenitors
Lung_and_airway
Olfactory_sensory_neurons
Hepatocytes
CNS_neurons
Neural_crest_PNS_glia
Muscle_cells
Ependymal_cells
Eye_and_other
Definitive_erythroid
Endothelium
Neural_crest_PNS_neurons
E8
E12
E16
P0

Figure EV7. *Kdm6a* is central for muscle differentiation and its co-expression with *Ccdc3 or Traf3ip3* identifies specific clusters of cells belonging to different embryonic tissues.

(**A**) Expression levels assessed by RT-qPCR of murine *Kdm6a* in C2C12 cell line. Mean +/− SEM ($n = 3$). Unpaired t test (*$p = 0.02$). (**B**) On the left, electrophoretic run of genomic DNA highlighting *Kdm6a* exon24 deletion after tamoxifen treatment. On the right, gene expression of *Kdm6a* (exon 24) assessed by RT-qPCR. Normalized on *GAPDH*. Mean +/− SD of three independent biological replicates. Unpaired t test (**$p = 0.0018$) (**C**) One representative image of MyHC (Myosin Heavy Chain) staining on satellite cells from mice. Fusion index percentage of control and *Kdm6a* KD cells. Mean +/− SD ($n = 3$). Unpaired t test (*$p = 0.0222$). (**D**) GSEA (v4.3.2) of DEGs from RNAseq data collected in WT and *Kdm6a* KO SCs after CTX treatment. Pathways derived from HALLMARK. (**E**) UMAP representation of spatial transcriptomic data showing *Kdm6a/Ccdc3* and *Kdm6a/Traf3ip*3 co-expressions, while (**F**) shows the *Kdm6a/Ccdc3*, *Kdm6a/Traf3ip3* and a third unknown cluster and (**G**) represents *Kdm6a* expression. (**H**) H&E staining and clustering of spatial transcriptomics data from mouse embryo at day 15.5. GSEA, v4.3.2 was performed on the genes differentially expressed within the cluster 3. (**I**) Heatmap showing the co-expression patterns of *Kdm6a* (ρKdm6a) with *Traf3ip3* and *Ccdc3* across others embryonic tissues during development.

 © The Author(s)
