# Supplementary material for: KDM6A loss enhances oxidative phosphorylation uncovering tissue-level convergent evolution
Source: EMBO J. 2026 Sep 4;45(19):6847–82. doi: 10.1038/s44318-026-00891-0 (PMC13631268; doi:10.1038/s44318-026-00891-0)
Supplement: Supplementary file 2 [file 44318_2026_891_MOESM2_ESM.pdf]

THE EMBO JOURNAL

KDM6A Loss Enhances Oxidative Phosphorylation Uncovering Tissue-Level Convergent Evolution

Antonino Alex Cartalemi, dalia rosano, camilla ferrari, gemma crupi, Laura Cassina, guido gatti, Federica Corigliano, simona punzi, Ilaria Villanti, Daphne Dupere Richer, Chiara D'Ercole, Veronica Ruggieri, Annarita Miluzio, Tiziana Bruno, Jose Garcia Manteiga, Valentina Giansanti, Richard Bennet, Davide Cittaro, luca madaro, Alessandra Boletta, Telmo Pievani, Stefano Biffo, Maurizio Fanciulli, Jonathan D. Licht, and Giovanni Tonon

Corresponding author(s): Giovanni Tonon (tonon.giovanni@hsr.it)

Review Timeline:

Submission Date:	7th Jan 26
Editorial Decision:	12th Feb 26
Revision Received:	5th Jun 26
Editorial Decision:	22nd Jul 26
Revision Received:	25th Jul 26
Accepted:	27th Jul 26

Editor: Hartmut Vodermaier

Transaction Report:

(Note: With the exception of the correction of typographical or spelling errors that could be a source of ambiguity, letters and reports are not edited. Depending on transfer agreements, referee reports obtained elsewhere may or may not be included in this compilation. Referee reports are anonymous unless the Referee chooses to sign their reports.)

1st Editorial Decision 12th Feb 2026

Dr. Giovanni Tonon
IRCCS San Raffaele Scientific Institute
Functional Genomics of Cancer Unit, Division of Molecular Oncology
Via Olgettina 60
Milan 20032
Italy

12th Feb 2026

Re: EMBOJ-2026-123503
KDM6A Loss Enhances Oxidative Phosphorylation Uncovering Tissue-Level Convergent Evolution

Dear Giovanni,

Thank you again for submitting your full manuscript on differential KDM6A roles in controlling oxidative phosphorylation in cancers to The EMBO Journal. We have now received feedback from three expert referees, copied below for your information. As you will see, the reviewers find your results potentially interesting, but also note that additional insights into various aspects would be important to make the study a compelling candidate for EMBO Journal publication. In this light, I would like to give you the opportunity to respond by way of a revised version of the manuscript. However, since it is our policy to consider only a single round of major revision, it will be important to not only fully respond to all comments at the time of resubmission, but also to extend the study along the lines suggested. Therefore, I would encourage you to contact me with a revision plan already during the early stages of your revision work, so that we could discuss how the main points might best be resolved, and which points would have the highest priority. We would also be open to extension of the default three-months revision period if needed; our 'scooping protection' (meaning that competing work appearing elsewhere in the meantime will not affect our considerations of your study) would remain valid also throughout such an extension.

Detailed information on preparing, formatting and uploading a revised manuscript can be found below and in our Guide to Authors. Thank you again for the opportunity to consider this work for The EMBO Journal, and I look forward to hearing back from you.

With kind regards,

Hartmut

Hartmut Vodermaier, PhD
Senior Editor, The EMBO Journal
h.vodermaier@embojournal.org

*** PLEASE NOTE: All revised manuscript are subject to initial checks for completeness and adherence to our formatting guidelines. Revisions may be returned to the authors and delayed in their editorial re-evaluation if they fail to comply to the following requirements. As a first step please read our guidelines for revised submissions:
https://link.springer.com/journal/44318/submission-guidelines#cms-Revised-submissions

1) Every manuscript requires a Data Availability section (even if only stating that no deposited datasets are included). Primary datasets or computer code produced in the current study have to be deposited in appropriate public repositories prior to resubmission, and reviewer access details provided in case that public access is not yet allowed.

2) Each figure legend must specify
- size of the scale bars that are mandatory for all micrograph panels
- the statistical test used to generate error bars and P-values
- the type error bars (e.g., S.E.M., S.D.)
- the number (n) and nature (biological or technical replicate) of independent experiments underlying each data point
- Figures may not include error bars for experiments with n<3; scatter plots showing individual data points should be used instead.

3) Revised manuscript text (including main tables, and figure legends for main and EV figures) has to be submitted as editable text file (e.g., .docx format). We encourage highlighting of changes (e.g., via text color) for the referees' reference.

4) Each main and each Expanded View (EV) figure should be uploaded as individual production-quality files (preferably in .eps,

.tif, .jpg formats). For suggestions on figure preparation/layout, please refer to our Figure Preparation Guidelines: https://media.springernature.com/original/springer-cms/rest/v1/content/27825798/data/v1

5) Point-by-point response letters should include the original referee comments in full together with your detailed responses to them (and to specific editor requests if applicable), and also be uploaded as editable (e.g., .docx) text files.

6) Please complete our Author Checklist, and make sure that information entered into the checklist is also reflected in the manuscript; the checklist will be available to readers as part of the Review Process File.

7) All authors listed as (co-)corresponding need to deposit, in their respective author profiles in our submission system, a unique ORCiD identifier linked to their name. Please see our Guide to Authors for detailed instructions.

8) Please note that supplementary information at EMBO Press has been superseded by the 'Expanded View' for inclusion of additional figures, tables, movies or datasets; with up to five EV Figures being typeset and directly accessible in the HTML version of the article.

9) To facilitate reproducibilty and cross-laboratory adoption of methodologies, please structure the Materials & Methods section as outlined in our guide to authors, including a completed Reagents and Tools Table.

10) Digital image enhancement is acceptable practice, as long as it accurately represents the original data and conforms to community standards. If a figure has been subjected to significant electronic manipulation, this must be clearly noted in the figure legend and/or the 'Materials and Methods' section. The editors reserve the right to request original versions of figures and the original images that were used to assemble the figure. Finally, we generally encourage uploading of numerical as well as gel/blot image source data.

At EMBO Press we ask authors to provide source data for the main manuscript figures. You will receive a separate email with instructions for providing source data with your revised manuscript, including how to upload and organize the files.

In the interest of ensuring the conceptual advance provided by the work, we recommend submitting a revision within 3 months (13th May 2026). Please discuss the revision progress ahead of this time with the editor if you require more time to complete the revisions. Use the link below to submit your revision:

Link Not Available

Referee #1:

In this manuscript, Cartalemi et al. investigate the role of the histone demethylase KDM6A in the regulation of oxidative phosphorylation. Loss of KDM6A has been associated with activation of OXPHOS in multiple tumor types, potentially representing an adaptive mechanism that supports tumor growth. The authors demonstrate that KDM6A regulates OXPHOS in a tissue-specific and context-dependent manner. In solid tumors, KDM6A is required for the expression of CCDC3, which suppresses CREB1-driven PPARGC1A transcription and limits mitochondrial biogenesis. In contrast, in multiple myeloma (MM), loss of KDM6A promotes mitochondrial transfer from stromal cells through TRAF3IP3-mTORC1 signaling. Together, these findings establish KDM6A as a central, context-dependent modulator of mitochondrial activity.

Conceptually, some findings are novel, particularly the work identifying a role for KDM6A in regulating mitochondrial transfer from stromal to MM cells. In contrast, the section focused on solid tumors and OXPHOS is less developed and has been reported previously in various contexts. In Figure 7, a mouse model is presented, but it is underdeveloped and it is unclear how it integrates with the rest of the study. Overall, while the manuscript contains interesting observations, it lacks coherence-shifting between systems-and remains largely descriptive without firmly establishing causality.

1. The conclusions in MM are based on a single cell line (ARP1). The manuscript would be strengthened by validating the findings in additional MM cell lines.
2. In Figure 6F, shKDM6A increases 4E-BP1 phosphorylation but not S6 phosphorylation under serum-free conditions (0'). Since both are downstream of mTOR, what is the proposed explanation for this discrepancy?
3. KDM6A is a histone H3K27me3 demethylase. Are any of the observed phenotypes dependent on its demethylase activity? More broadly, how does KDM6A regulate adaptation to OXPHOS in solid tumors versus MM?

Referee #2:

This comprehensive study shows that loss of the epigenetic player KDM6A increases oxidative phosphorylation across cancers and normal tissues, highlighting distinct, tissue-specific mechanisms. In solid tumors, KDM6A loss enhances mitochondrial biogenesis via a CCDC3-CREB1-PPARGC1A axis, whereas in multiple myeloma, it activates mTORC1 and promotes mitochondrial transfer from stromal cells. The authors propose that KDM6A is a key controller of metabolism, illustrating a form of tissue-level convergent evolution toward increased mitochondrial activity.

I am outlining a few comments to help clarify aspects of the study:
The functional relationship between OXPHOS and tumor fitness can be better studied in the manuscript: can the authors genetically inhibit PPARGC1A or perform mitochondrial transfer to affect tumor maintenance in KDM6A-deficient settings without systematically demonstrating necessity in vivo?

A key question is whether the mechanisms described in the different settings are dependent on the KDM6A demethylase activity or reflect catalytic-independent (scaffolding/cofactor-recruitment) functions. The H3K27 marks are shown at selected loci (such as TRAF3IP3), and the manuscript would benefit from comparison between wild-type and catalytically dead rescue experiments in KDM6A knockout settings. Further clarifying this point would substantially strengthen the mechanistic interpretation.

Although the CCDC3-CREB1-PPARGC1A axis is well dissected in solid tumors, the mechanisms in normal tissues are not similarly dissected, and the conclusions depend on correlative transcriptomic analyses. Can the authors genetically suppress Ppargc1a/PGC-1α or restore Ccdc3 in Kdm6a-deficient muscle cells and test for potential OXPHOS changes and rescue of myogenic differentiation?

As the authors mention in the manuscript, all the models used are female to avoid UTY compensation. Can they discuss potential implication of UTY in male models?

Referee #3:

Very interesting and novel findings by Cartalemi et al. The authors show that in solid tumours, loss of KDM6A increases oxidative phosphorylation; however, this phenotype was not observed in the blood cancer multiple myeloma. Interestingly, when MM cell lines were co-cultured with the stromal cell line HS-5, the phenotype was restored, suggesting that the tumour microenvironment in myeloma is the limiting factor. Further work demonstrated that KDM6A knockout in myeloma induces mitochondrial transfer from stroma to myeloma cells via a TRAF3IP3-dependent mechanism. Although mitochondrial transfer has previously been identified in myeloma, this study provides a novel mechanistic insight. I have several additional comments below.
1. The authors use both MitoTracker dye and a genetic mitochondrial tag to demonstrate mitochondrial transfer. This dual approach is robust and should be commended.
2. The authors show increased mitochondrial mass in solid tumours in the KDM6A knockout model (Figure 1F). It would strengthen the manuscript to show equivalent data in the myeloma patient samples.
3. It would be valuable to clarify whether solid tumours also engage in mitochondrial transfer in this context, or whether this mechanism is specific to myeloma.
4. There is heavy reliance on a single MM cell line in the study. This raises questions about relevance of the findings to human disease. Inclusion of primary human samples would significantly strengthen the manuscript.
5. The authors should consider investigating mitochondrial transfer in vivo. Do tumours with KDM6A have increased mitochondrial mass in vivo and have a growth advantage, or increased chemo resistance, or is this phenomenon limited to in-vitro systems?
6. Many experiments appear to have n=3, and in some cases inappropriate statistical tests have been used to infer significance. The authors should consult with a statistician to ensure correct statistical interpretation.

Milan, June 5, 2026

Dear Dr. Vodermaier,

Many thanks for your help in shepherding this manuscript through the review process, and many thanks for the insightful and constructive comments of the Reviewers, which we greatly appreciated. Please find below a detailed point-by-point response.

Best regards,

Giovanni

Note on figure numbering: *In this document, Arabic numerals refer to the manuscript figures, while Roman numerals refer to figures included in this document.*

Referee #1:

In this manuscript, Cartalemi et al. investigate the role of the histone demethylase KDM6A in the regulation of oxidative phosphorylation. Loss of KDM6A has been associated with activation of OXPHOS in multiple tumor types, potentially representing an adaptive mechanism that supports tumor growth. The authors demonstrate that KDM6A regulates OXPHOS in a tissue-specific and context-dependent manner. In solid tumors, KDM6A is required for the expression of CCDC3, which suppresses CREB1-driven PPARGC1A transcription and limits mitochondrial biogenesis. In contrast, in multiple myeloma (MM), loss of KDM6A promotes mitochondrial transfer from stromal cells through TRAF3IP3-mTORC1 signaling. Together, these findings establish KDM6A as a central, context-dependent modulator of mitochondrial activity.

Conceptually, some findings are novel, particularly the work identifying a role for KDM6A in regulating mitochondrial transfer from stromal to MM cells. In contrast, the section focused on solid tumors and OXPHOS is less developed and has been reported previously in various contexts.

In Figure 7, a mouse model is presented, but it is underdeveloped and it is unclear how it integrates with the rest of the study.

We thank the reviewer for the overall positive assessment of our work. We included the mouse model to extend our findings beyond cancer, to a physiological context. We have tried to integrate more smoothly this section into the manuscript, adding some data to better connect the *in vitro* observations with the *in vivo* experiments (Fig. 7B).

Overall, while the manuscript contains interesting observations, it lacks coherence-shifting between systems-and remains largely descriptive without firmly establishing causality.

1. The conclusions in MM are based on a single cell line (ARP1). The manuscript would be strengthened by validating the findings in additional MM cell lines.

Following the reviewer's suggestion, we have now included two additional MM cell lines, EJM and AMO-1. Prompted by the observation of the third reviewer, we have also included data from 2 patient-derived MM models.

On the overall, we have confirmed that *KDM6A* loss downregulates TRAF3IP3 levels (Figure IA) also in these new models, as we described in ARP-1 and in 30 MM cell lines derived from the DepMap portal, as reported in the previous version of the manuscript (Figure EV6H). We also confirmed that PPARGC1A and mitochondrial mass did not change upon KDM6A KO in EJM (data already included in the previous version of the manuscript) as well as in AMO-1 (Figure IB-C). The new data gathered from the two MM primary cell lines also confirmed these observations (Figure ID-E).

Finally, we explored mitochondrial trafficking between stromal and MM cells also in the EJM and AMO-1 MM cell lines. Following the approach described in the manuscript (as performed in figure 4C-D for the ARP-1 MM cell line in the previous version of the manuscript), we confirmed that in these new MM cell lines there was an increment in mitochondrial trafficking upon HS-5 coculture, which was prevented by Rapamycin (mTOR inhibitor) treatment (Figure IF).

Overall, we confirmed on additional established and primary MM cell lines that KDM6A loss increases mitochondrial trafficking through the mTOR pathway via TRAF3IP3. We added these additional data to the main manuscript as Figures 3D-E, EV3D-F, 6K and EV6B.

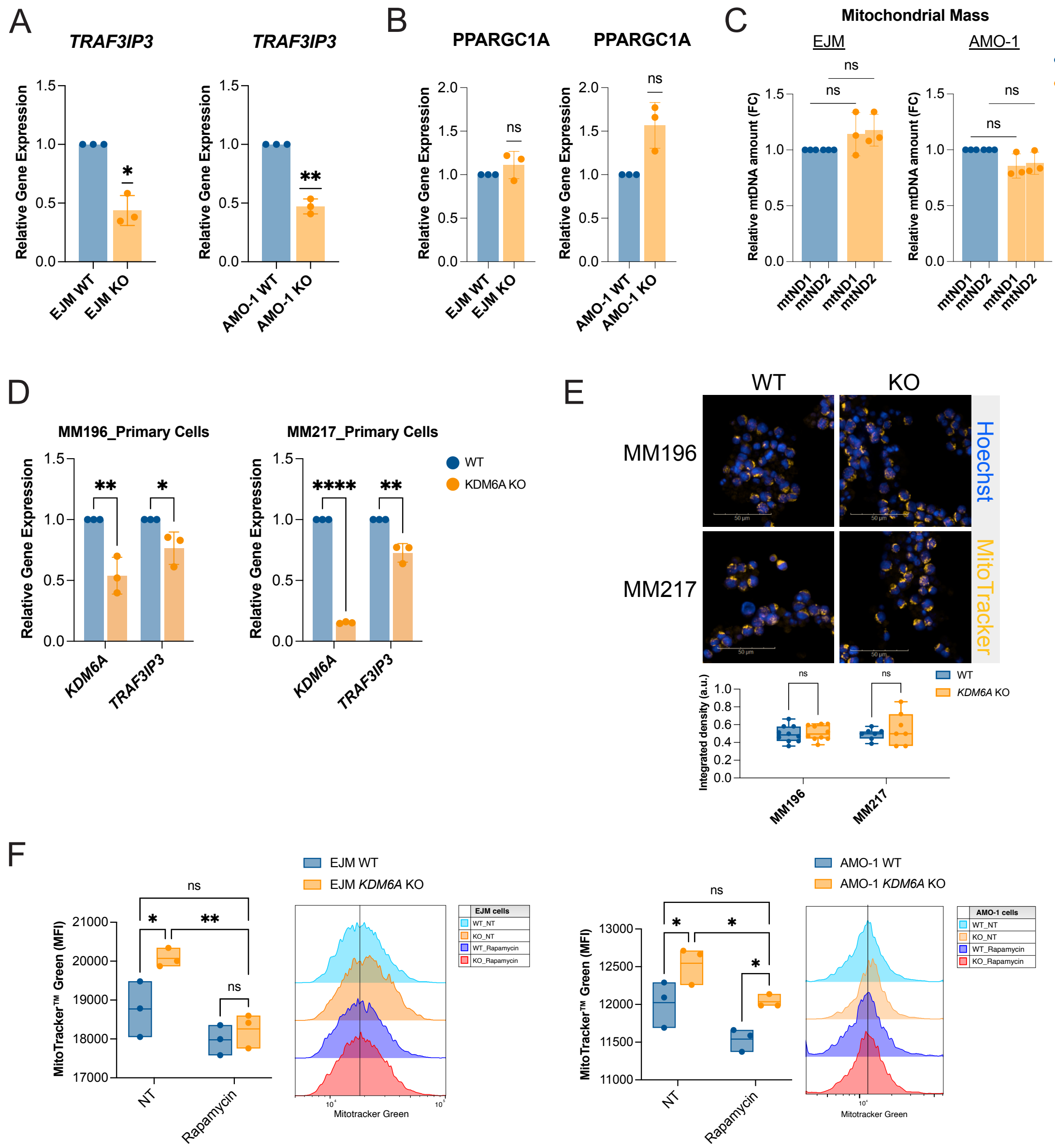

Figure I: mTOR pathway activation induces mitochondrial trafficking upon KDM6A loss in EJM MM cell line. (A) rtPCR on EJM and AMO-1 RNA. Normalization on TBP and WT control (three biological replicates; mean +/- SD; one sample t test). (**B**) rtPCR on EJM and AMO-1 RNA. Normalization on TBP and WT control (three biological replicates; mean +/- SD; one sample t test). (**C**) qPCR on EJM and AMO-1 DNA. Normalization on NBPF9 and WT control (three biological replicates; mean +/- SD; multiple unpaired t test). (**D**) rtPCR on MM196 and MM217 MM primary cell lines RNA. Normalization on ACTB and WT control (three biological replicates; mean +/- SD; multiple unpaired t test). (**E**) Mitotracker fluorescence measurement and quantification in Primary MM cell (7 to 10 acquisitions coming from three biological replicates; multiple t test). (**F**) Mitotracker MFI measurement by FACS upon coculture of EJM and AMO-1 with HS-5 and Rapamycin (50nM) treatment (MFI +/- SD, 2way Anova).

2. In Figure 6F, shKDM6A increases 4E-BP1 phosphorylation but not S6 phosphorylation under serum-free conditions (0'). Since both are downstream of mTOR, what is the proposed explanation for this discrepancy?

As emerging in the bar plots included in Figure 6G, there was some variability between different western blottings. We choose one representative western blotting in the manuscript, where the increase at baseline was not evident, while other blots did show this increase also at time 0, as for example in the blot included in Figure II.

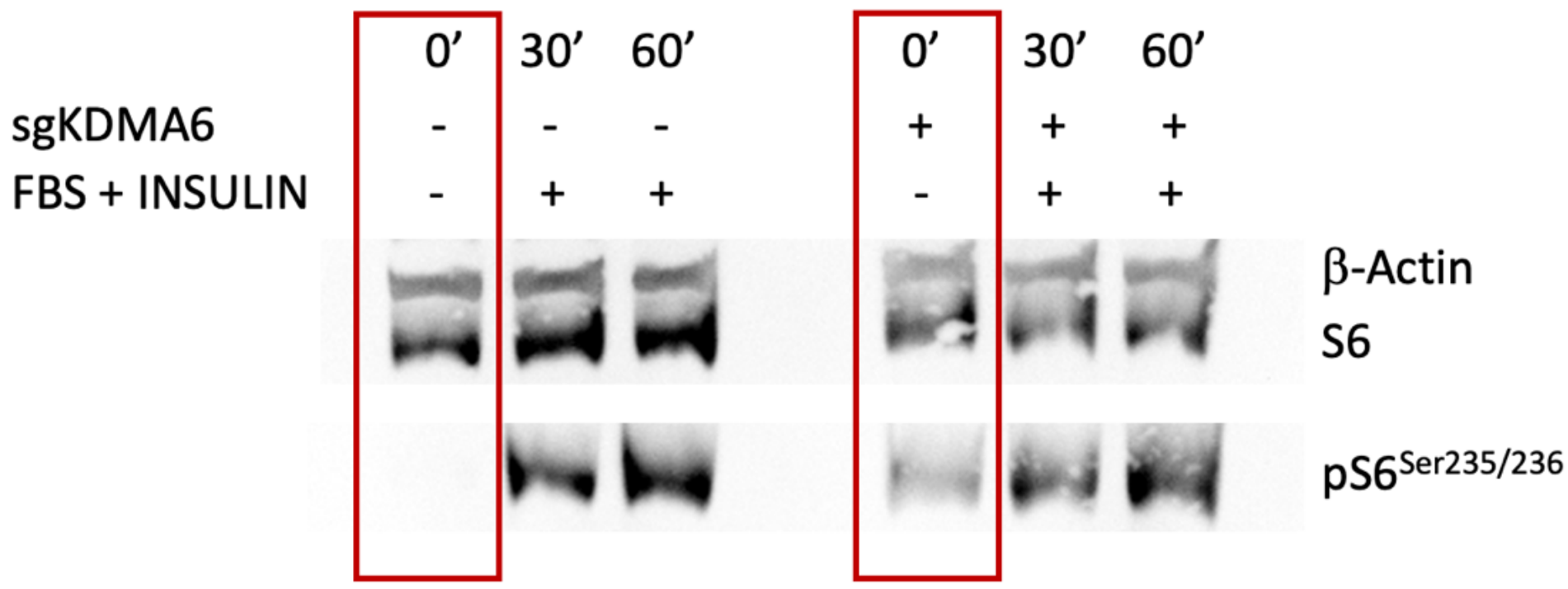

Figure II mTORC1 pathway induction in MM cells.

That said, while both 4E-BP1 and S6 are canonical downstream targets of mTORC1, their phosphorylation dynamics are not identical and can diverge depending on the cellular context and stimulus conditions: 4E-BP1 is a direct target of mTORC1, whereas rpS6 controlled by the mTORC1-p70SK and RS6K pathways (Saxton & Sabatini, 2017), suggesting that S6K-S6 branch may be subject to additional regulatory constraints under serum starvation (Böhm *et al*, 2021; Dowling *et al*, 2010; Thoreen *et al*, 2012).

3. KDM6A is a histone H3K27me3 demethylase. Are any of the observed phenotypes dependent on its demethylase activity? More broadly, how does KDM6A regulate adaptation to OXPHOS in solid tumors versus MM?

We are grateful to the reviewer for this constructive feedback.

In order to assess the relevance of the catalytic activity of KDM6A in the solid tumor model we transfected a catalytically-dead mutant KDM6A (pCS2-UTX-F-MT2, Addgene Plasmid #40619)(Wang *et al*, 2012), or a wild type (WT) isoform, in U-2 OS cells and assessed *PPARGC1A* expression (Figure IIIA). The overexpression of WT KDM6A downregulated PPARGC1A expression, as anticipated. However, also the expression of the catalytically-dead KDM6A (Figure IIIB) resulted in PPARGC1A

downregulation (Figure IIIC), suggesting that the effect of KDM6A on PPARGC1A is not explained by its catalytic function, but more likely by its scaffolding activity, which has been extensively described (Gozdecka *et al*, 2018; Pacella *et al*, 2025). Interestingly, in MM cells, the ChIPseq data reported in figure 6M show that KDM6A loss reduces H3K27me3 levels, the KDM6A specific target histone modification. These results suggest that an additional layer of context specificity defines the activity of KDM6A: in solid tumors, KDM6A acts as a scaffolding protein to control oxidative phosphorylation through its interaction with CCDC3, while in MM controls TRAF3IP3 via its demethylase activity. We added these results in figure 2C-D and commented them in the discussion.

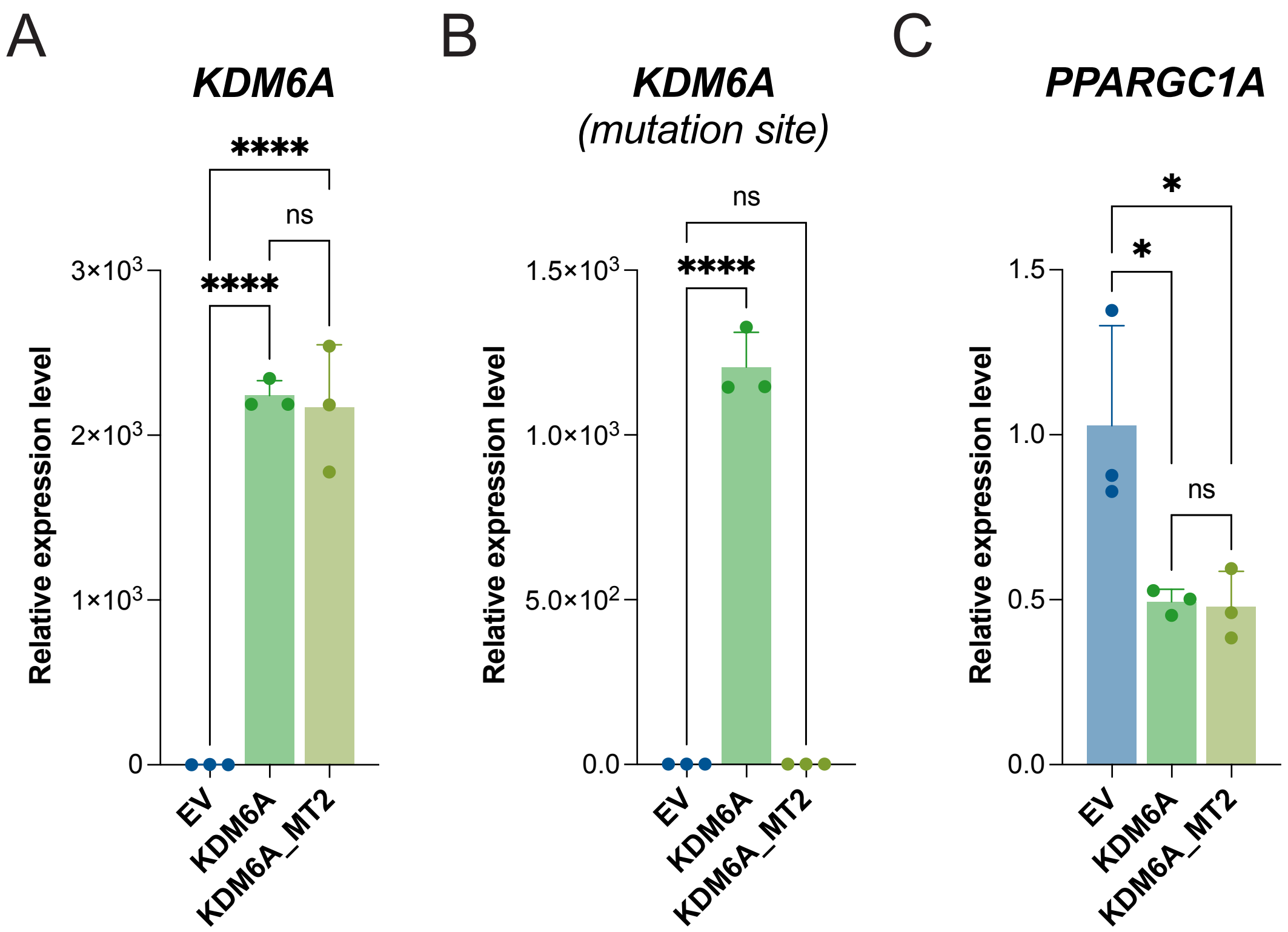

Figure III: PPARGC1A expression regulation is independent from KDM6A catalytic activity. rtPCR of U-2 OS RNA after transfection with empty vector (EV), KDM6A and catalytically dead KDM6A (KDM6A_MT2). (**A**) Evaluation of KDM6A expression, (**B**) KDM6A with primers designed on the mutation site and (**C**) PPARGC1A (three biological replicates; Mean +/- SD, One-way Anova).

Referee #2:

This comprehensive study shows that loss of the epigenetic player KDM6A increases oxidative phosphorylation across cancers and normal tissues, highlighting distinct, tissue-specific mechanisms. In solid tumors, KDM6A loss enhances mitochondrial biogenesis via a CCDC3-CREB1-PPARGC1A axis, whereas in multiple myeloma, it activates mTORC1 and promotes mitochondrial transfer from stromal cells. The authors propose that KDM6A is a key controller of metabolism, illustrating a form of tissue-level convergent evolution toward increased mitochondrial activity.

I am outlining a few comments to help clarify aspects of the study:

The functional relationship between OXPHOS and tumor fitness can be better studied in the manuscript: can the authors genetically inhibit PPARGC1A or perform mitochondrial transfer to affect tumor maintenance in KDM6A-deficient settings without systematically demonstrating necessity in vivo?

We thank the reviewer for the positive appraisal of our study, and specifically for raising this important point. We first attempted to interfere with the formation of TNTs, using cytochalasin B, which however was very toxic for all cells. To demonstrate a role for increased mitochondrial transfer and OXPHOS in driving proliferation, we utilized first mTOR inhibitors, which hampered the mitochondrial increase elicited by KDM6A as well as the increased proliferation driven by KDM6A (Figure 6E). Conversely, as reported in Figure S4D, we showed that cells lacking KDM6A proliferated more that their normal counterpart upon treatment with an inhibitor of the electron transport chain, suggesting that the increased mitochondrial load endowed them with an increased resistance to OXPHOS inhibition, again pointing to a role of the increase in mitochondria elicited by KDM6A loss in driving proliferation.

A key question is whether the mechanisms described in the different settings are dependent on the KDM6A demethylase activity or reflect catalytic-independent (scaffolding/cofactor-recruitment) functions. The H3K27 marks are shown at selected loci (such as TRAF3IP3), and the manuscript would benefit from comparison between wild-type and catalytically dead rescue experiments in KDM6A knockout settings. Further clarifying this point would substantially strengthen the mechanistic interpretation.

We are grateful to the reviewer for this constructive feedback.

In order to assess the relevance of the catalytic activity of KDM6A in the solid tumor model we transfected a catalytically-dead mutant KDM6A (pCS2-UTX-F-MT2, Addgene Plasmid #40619)(Wang *et al*, 2012), or a wild type (WT) isoform, in U-2 OS cells and assessed *PPARGC1A* expression (Figure IVA). The overexpression of WT KDM6A downregulated PPARGC1A expression, as anticipated.

However, also the expression of the catalytically-dead KDM6A (Figure IVB) resulted in PPARGC1A downregulation (Figure IVC), suggesting that the effect of KDM6A on PPARGC1A is not explained by its catalytic function, but more likely by its scaffolding activity, which has been extensively described (Gozdecka *et al*, 2018; Pacella *et al*, 2025). Interestingly, in MM cells, the ChIPseq data reported in figure 6M show that KDM6A loss reduces H3K27me3 levels, the KDM6A specific target histone modification. These results suggest that an additional layer of context specificity defines the activity of KDM6A: in solid tumors, KDM6A acts as a scaffolding protein to control oxidative phosphorylation through its interaction with CCDC3, while in MM controls TRAF3IP3 via its demethylase activity. We added these results in figure 2C-D and commented them in the discussion.

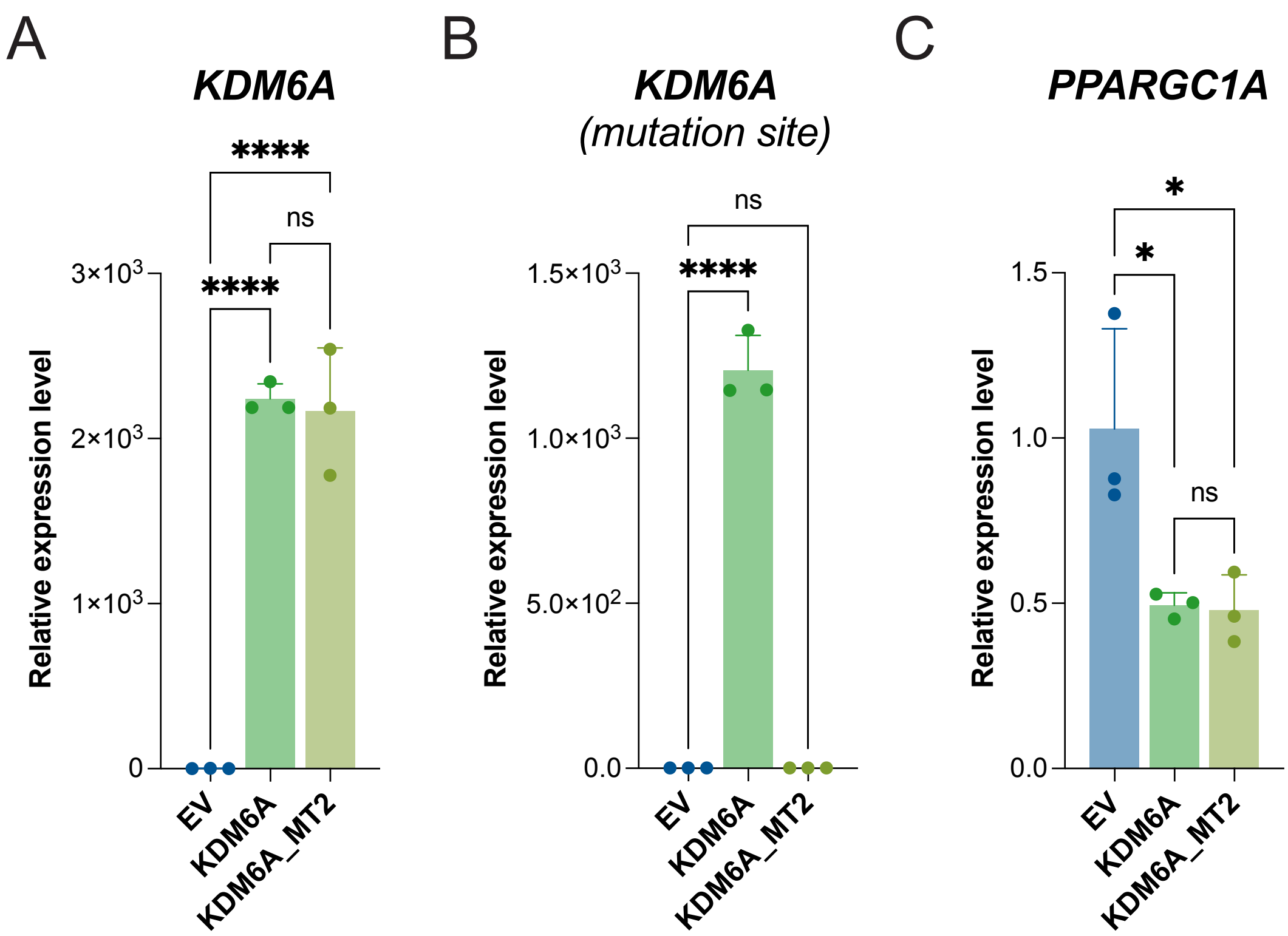

Figure IV: PPARGC1A expression regulation is independent from KDM6A catalytic activity. rtPCR of U-2 OS RNA after transfection with empty vector (EV), KDM6A and catalytically dead KDM6A (KDM6A_MT2). (**A**) Evaluation of KDM6A expression, (**B**) KDM6A with primers designed on the mutation site and (**C**) PPARGC1A (three biological replicates; Mean +/- SD, One-way Anova).

Although the CCDC3-CREB1-PPARGC1A axis is well dissected in solid tumors, the mechanisms in normal tissues are not similarly dissected, and the conclusions depend on correlative transcriptomic analyses. Can the authors genetically suppress Ppargc1a/PGC-1α or restore Ccdc3 in Kdm6a-deficient muscle cells and test for potential OXPHOS changes and rescue of myogenic differentiation?

We thank the reviewer for raising this important point. As previously shown, also in C2C12 *Kdm6a* KO increased *Ppargc1a* levels (Figure 7A in the manuscript; below, VA), thus suggesting a direct role of Kdm6a to regulate this gene. As suggested, we have then knocked down *Ppargc1a* in muscle cells and assessed the impact on myogenic differentiation. *Kdm6a* KO alone reduced both the mean number of nuclei and the fusion index of the terminal myotubes (Figure VB) in line with previous studies (Seenundun *et al*, 2010; Faralli *et al*, 2016). We then tested the impact of *Ppargc1a* knock-down. As shown in figure VB, Ppargc1a loss *per se* interferes with myogenic differentiation, as the mean number of nuclei and the fusion index of the terminal myotubes was reduced upon Ppargc1a loss. Combining knock-down of *Kdm6a* and *Ppargc1a* did not rescue the phenotype, suggesting that the impairment of muscle development elicited by *Kdm6a* loss is not mediated by *Ppargc1a*.

We then explored also the RNA levels of *Ppargc1a* during differentiation, which was obtained in C2C12 cells by switching from growth medium (GM) to differentiation medium (DM). *Ppargc1a* expression was induced during differentiation (figure VC), in line with previous reports (Baldelli *et al*, 2014), suggesting a role for Ppargc1a during muscle development. Instead, we noticed that Ppargc1a expression increase upon KO was rescued upon differentiation.

In conclusion, while Kdm6a loss increases Ppargc1a levels in undifferentiated cells, as seen in solid tumors, the roles of Kdm6a and Ppargc1a in myogenic differentiation is difficult to disentangle, as Ppargc1a interferes *per se* with muscle development when is downregulated (after siRNAs). However, Ppargc1a upregulation (as a result of *kdm6a* knock-down) does not seem to be the cause of the differentiation impairment induced by Kdm6a loss, since Ppargc1a downregulation had no effect on differentiation. Based on these data, we could only assess the impact of kdm6a on Ppargc1a levels in undifferentiated myoblasts, with no conclusive model on how this increase on Ppargc1a elicited by *kdm6a* KO may affect muscle development. We included panel C into Figure 7 of the manuscript to better characterize the role of Kdm6a loss in Ppargc1a regulation during differentiation.

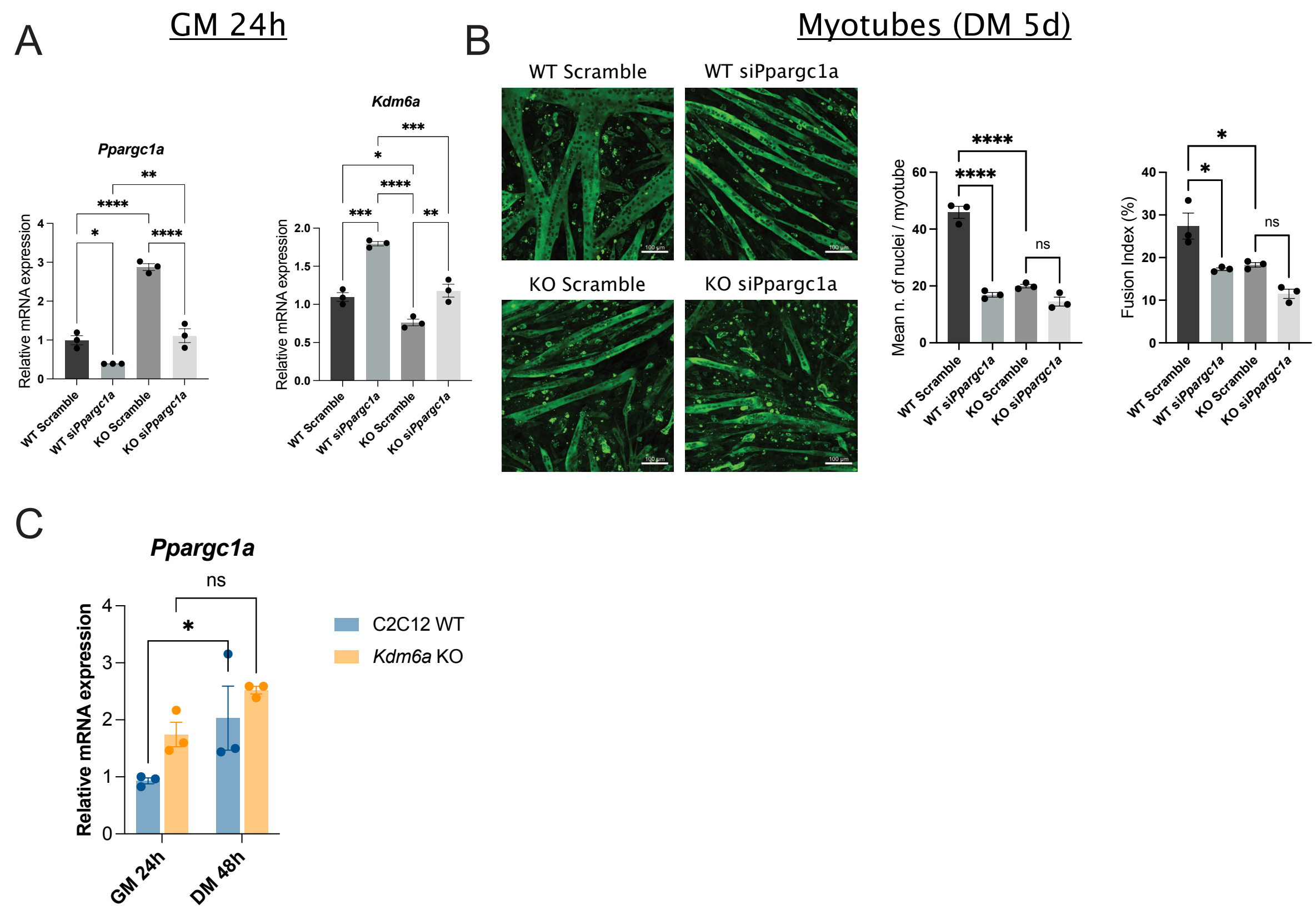

Figure V: *Ppargc1a* inhibition during C2C12 differentiation impairs C2C12 differentiation. (**A**) *Ppargc1a* and *Kdm6a* expression level upon *Kdm6a* KO and si*Ppargc1a* (three biological replicates; mean +/- SEM, one-way Anova). (**B**) Myosin Heavy Chain staining of 5 days fully differentiated myotubes and quantification of nuclei number and fusion index (three biological replicates; mean +/- SEM, one-way Anova). (**C**) *Ppargc1a* expression level of C2C12 in growth medium (GM) or differentiation medium (DM) (three biological replicates; mean +/- SEM, 2way Anova).

As the authors mention in the manuscript, all the models used are female to avoid UTY compensation. Can they discuss potential implication of UTY in male models?

The role of UTY is still incompletely understood. Concerning its role in stem cell differentiation, KDM6A promotes embryonic stem cells mesodermal differentiation independently from its demethylase activity (Wang *et al*, 2012). UTY, which presents a negligible demethylase activity (Hong *et al*, 2007), restores differentiation in male *KDM6A* KO stem cells (Wang *et al*, 2012), suggesting a compensatory effect despite the lack of demethylase activity. Moreover, homozygous *Kdm6a* KO murine embryos show dramatic morphogenic defects more severe in female than males, likely due to the redundant role of *Uty*. Indeed, *Kdm6a* KO females die during embryonic development, while a subset of males complete embryonic development albeit with reduced size and survival (Welstead *et al*, 2012) thus showing a partial rescue of the lethality caused by *Kdm6a* loss. On the other hand, the loss of Kdm6a in CD4+ T-cell precursors impairs the expression of the terminal thymocyte differentiation gene *S1pr1* (Manna *et al*, 2015), a phenotype not rescued by Uty, probably for an insufficient activity of its demethylase domain.

In line with the existing literature, we explored whether there were differences in patient data or own models, between females and males, with the assumption that the presence of UTY in male samples may temper some of the phenotypes emerging from KDM6A losses. In the transcriptomic data obtained from the cancer samples of male patients, OXPHOS remains among the most significantly enriched pathways impacted by low KDM6A levels both in solid tumors (figure VIA) and in Multiple Myeloma (figure VIB). Additionally, among the MM cell lines that we examined to address some of the points raised by the other reviewers, we included also two MM primary samples, MM196 (male sample) and MM217 (female sample). In both cases, irrespectively of the gender, mitochondrial mass remained unchanged (figure VIC) and TRAF3IP3 was downregulated upon KDM6A KO (figure VID). Overall, we conclude that also in our system, UTY is unable to compensate for the increase in OXPHOS elicited by KDM6A loss.

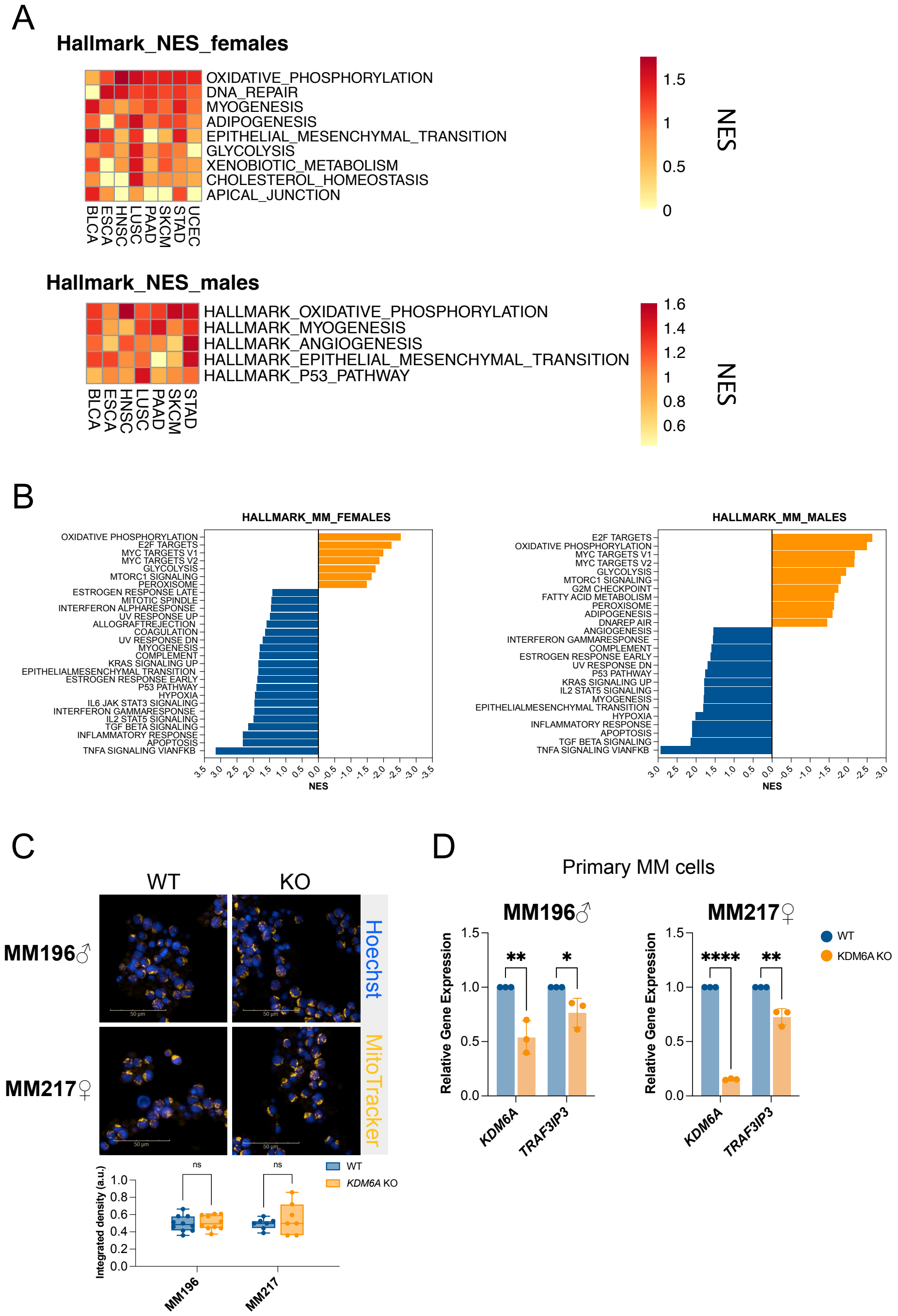

Figure VI: Male patients exhibit the same phenotype as female patients. (**A**) Top 8 tumors ranked by *KDM6A* alteration frequency selected for transcriptomics analysis by GSEA. Positive NES indicates enriched pathways in *KDM6A* low expression patients (lower quartile). Pathways were derived from HALLMARK. TCGA patient dataset: Urothelial Bladder Carcinoma (BLCA), Esophageal Carcinoma (ESCA), Head-Neck Squamous Cell Carcinoma (HNSC), Lung Squamous Cell Carcinoma (LUSC), Pancreatic Adenocarcinoma (PAAD), Skin Cutaneous Melanoma (SKCN), Stomach Adenocarcinoma (STAD), Uterine Corpus Endometrial Carcinoma (UCEC). (**B**) GSEA analysis of transcriptome data from MMRF CoMMpass™ study patients' cohort. Negative NES represent pathways anticorrelated with *KDM6A* expression in MM patients. Pathways derived from HALLMARK. (**C**) Measurement of the median integrated density of Mitotracker Red signal. Images acquired using the Opera confocal microscope, 7 to 10 different acquisitions coming from three independent biological replicates. (**D**) RNA levels of *KDM6A* and TRAF3IP3 in WT and *KDM6A* KO MM primary cells. Data are normalized to *ACTB* and to WT and represented as mean (+/- SD) of three independent biological replicates.

Referee #3:

Very interesting and novel findings by Cartalemi et al. The authors show that in solid tumours, loss of KDM6A increases oxidative phosphorylation; however, this phenotype was not observed in the blood cancer multiple myeloma. Interestingly, when MM cell lines were co-cultured with the stromal cell line HS-5, the phenotype was restored, suggesting that the tumour microenvironment in myeloma is the limiting factor. Further work demonstrated that KDM6A knockout in myeloma induces mitochondrial transfer from stroma to myeloma cells via a TRAF3IP3-dependent mechanism. Although mitochondrial transfer has previously been identified in myeloma, this study provides a novel mechanistic insight. I have several additional comments below.

We thank the reviewer for the enthusiasm demonstrated for our study.

1. The authors use both MitoTracker dye and a genetic mitochondrial tag to demonstrate mitochondrial transfer. This dual approach is robust and should be commended.

We thank the reviewer.

2. The authors show increased mitochondrial mass in solid tumours in the KDM6A knockout model (Figure 1F). It would strengthen the manuscript to show equivalent data in the myeloma patient samples.

We thank the reviewer for this intriguing suggestion. MM patients are not included in the study we utilized for the assessment of mitochondrial DNA in patients (Reznik *et al*, 2016). In order to add data on MM patients we requested access to data from the CoMMpass study of the MMRF repository. However, after several interactions with the team managing the repository, we learned that genome alignments or raw reads are not provided to the community, but only VCF files, which do not include the mitochondrial genome. With regret, we were therefore unable to address this interesting point.

3. It would be valuable to clarify whether solid tumours also engage in mitochondrial transfer in this context, or whether this mechanism is specific to myeloma.

We appreciate the reviewer's concern regarding this issue. It should be mentioned that our data suggest that this transfer is driven by *TRAF3IP3*, a gene that we show specifically expressed in haematological cancer cells (Figure S6F-G), thus suggesting that mitochondrial trafficking should not be involved in solid tumors.

4. There is heavy reliance on a single MM cell line in the study. This raises questions about relevance of the findings to human disease. Inclusion of primary human samples would significantly strengthen the manuscript.

Obtaining primary cells from myeloma patients is complex, and infection/transfection of these cells is technically challenging. Through a collaboration with Dr. Fanciulli's team, now co-author, we have been able to test the role of KDM6A in two primary samples derived from MM patients (Bruno *et al*, 2020). We have knocked out KDM6A expression (Figure VIIA) and after 48h from electroporation we evaluated mitochondrial mass through Mitotracker signal acquired using the Opera microscope (Figure VIIB). Moreover, we assessed TRAFIP3 expression upon KDM6A loss (Figure VIIC). Overall, we confirmed data collected in MM cell lines.

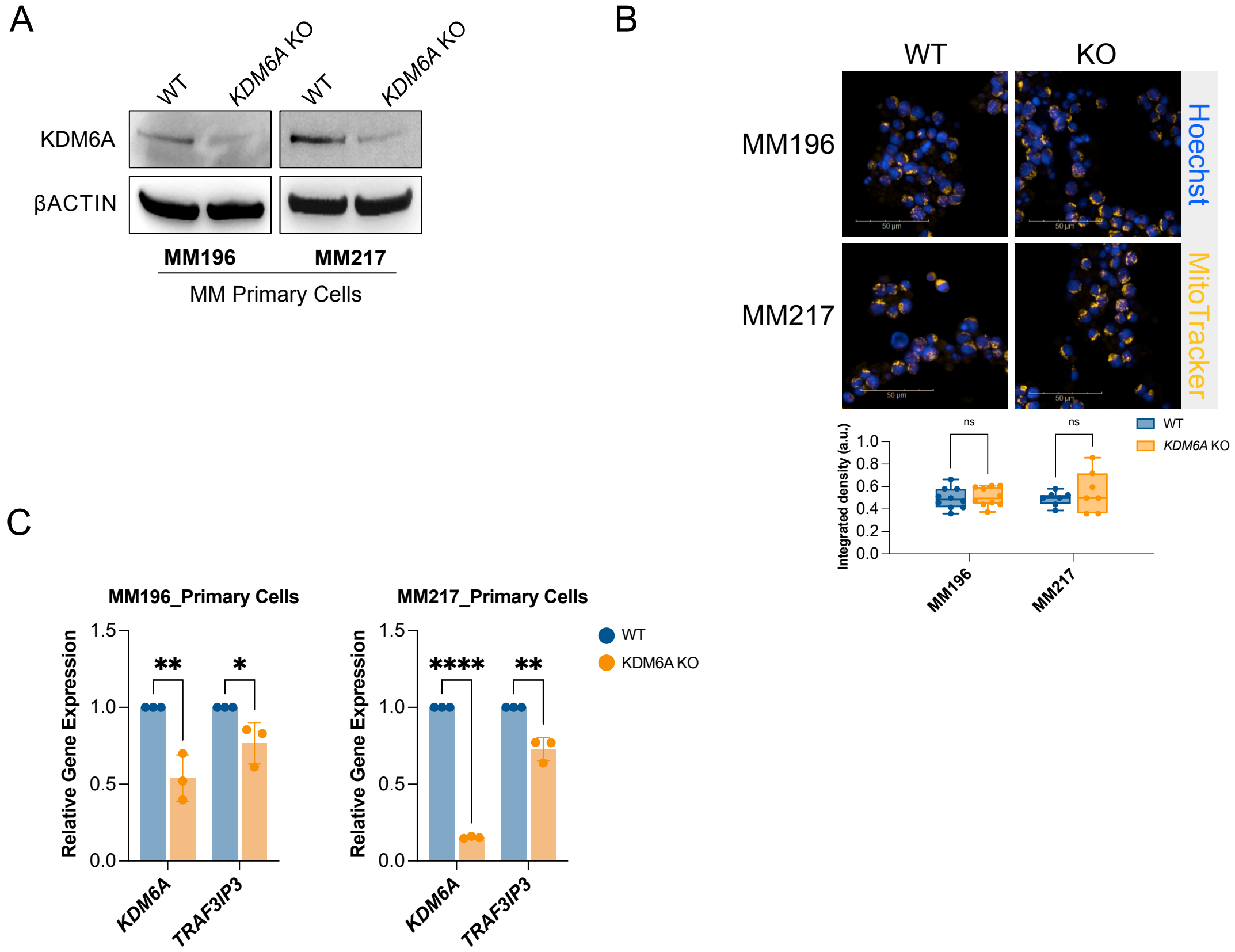

Figure VII: MM primary cells recapitulate cell lines. (**A**) Western blot analysis of KDM6A protein level. (**B**) Measurement of the median integrated density of Mitotracker Red signal. Images acquired using the Opera confocal microscope, 7 to 10 different acquisitions coming from three independent biological replicates. (**C**) RNA levels of *KDM6A* and TRAF3IP3 in WT and *KDM6A* KO MM primary cells. Data are normalized to *ACTB* and to WT and represented as mean (+/- SD) of three independent biological replicates.

5. The authors should consider investigating mitochondrial transfer in vivo. Do tumours with KDM6A have increased mitochondrial mass in vivo and have a growth advantage, or increased chemo resistance, or is this phenomenon limited to in-vitro systems?

At this stage, establishing a new *in vivo* model would require substantial optimization and set-up, including obtaining the authorization from the Italian Ministry of Health that requires several months and seems to go beyond what can be realistically achieved during the revision process. While we fully appreciate the reviewer's suggestion and agree that such experiments would be of great interest, we would argue they are more appropriate for a dedicated follow-up study rather than for the current manuscript.

6. Many experiments appear to have n=3, and in some cases inappropriate statistical tests have been used to infer significance. The authors should consult with a statistician to ensure correct statistical interpretation.

In line with the number of biological replicates that we usually find in the literature, we trusted that 3 was the minimal number allowed, reassured also by some journals guidelines such as Nature Communications(https://www.nature.com/documents/Biological_and_technical_replicates_guidelines.pdf). We tried to find additional guidelines from other journals, but we could not find exact numbers mentioned. With the help of a statistician, we have revised the manuscript to improve it wherever possible. We decided to change the violin plot in Figure 4D towards a more fitting scatter plot. Moreover, we implemented the one sample t test each time a normalization to 1 was necessary. Finally, we will specify in the manuscript that the normalization of all samples to 1 was adopted when the experiment entailed paired samples. An equalizer or raw measurement were plotted in the case of unpaired samples. These clarifications have been included in the statistics paragraph of Materials and methods.

References

Baldelli S, Aquilano K & Ciriolo MR (2014) PGC-1α buffers ROS-mediated removal of mitochondria during myogenesis. *Cell Death Dis* 5: e1515–e1515

Böhm R, Imseng S, Jakob RP, Hall MN, Maier T & Hiller S (2021) The dynamic mechanism of 4E-BP1 recognition and phosphorylation by mTORC1. *Mol Cell* 81: 2403-2416.e5

Bruno T, De Nicola F, Corleone G, Catena V, Goeman F, Pallocca M, Sorino C, Bossi G, Amadio B, Cigliana G, *et al* (2020) Che-1/AATF-induced transcriptionally active chromatin promotes cell proliferation in multiple myeloma. *Blood Adv* 4: 5616–5630

Dowling RJO, Topisirovic I, Alain T, Bidinosti M, Fonseca BD, Petroulakis E, Wang X, Larsson O, Selvaraj A, Liu Y, *et al* (2010) mTORC1-Mediated Cell Proliferation, But Not Cell Growth, Controlled by the 4E-BPs. *Science* 328: 1172–1176

Faralli H, Wang C, Nakka K, Benyoucef A, Sebastian S, Zhuang L, Chu A, Palii CG, Liu C, Camellato B, *et al* (2016) UTX demethylase activity is required for satellite cell–mediated muscle regeneration. *J Clin Invest* 126: 1555–1565

Gozdecka M, Meduri E, Mazan M, Tzelepis K, Dudek M, Knights AJ, Pardo M, Yu L, Choudhary JS, Metzakopian E, *et al* (2018) UTX-mediated enhancer and chromatin remodeling suppresses myeloid leukemogenesis through noncatalytic inverse regulation of ETS and GATA programs. *Nat Genet* 50: 883–894

Hong S, Cho Y-W, Yu L-R, Yu H, Veenstra TD & Ge K (2007) Identification of JmjC domain-containing UTX and JMJD3 as histone H3 lysine 27 demethylases. *Proc Natl Acad Sci* 104: 18439–18444

Manna S, Kim JK, Baugé C, Cam M, Zhao Y, Shetty J, Vacchio MS, Castro E, Tran B, Tessarollo L, *et al* (2015) Histone H3 Lysine 27 demethylases Jmjd3 and Utx are required for T-cell differentiation. *Nat Commun* 6: 8152

Pacella GN, Kuprasertkul N, Bao L, Huang S, D'souza C, Prouty SM, Anderson A, Maldonado López AM, Sinkfield M, Olingou C, *et al* (2025) UTX (KDM6A) promotes differentiation noncatalytically in somatic self-renewing epithelia. *Proc Natl Acad Sci* 122: e2422971122

Reznik E, Miller ML, Şenbabaoğlu Y, Riaz N, Sarungbam J, Tickoo SK, Al-Ahmadie HA, Lee W, Seshan VE, Hakimi AA, *et al* (2016) Mitochondrial DNA copy number variation across human cancers. *eLife* 5: e10769

Saxton RA & Sabatini DM (2017) mTOR Signaling in Growth, Metabolism, and Disease. *Cell* 168: 960–976

Seenundun S, Rampalli S, Liu Q-C, Aziz A, Palii C, Hong S, Blais A, Brand M, Ge K & Dilworth FJ (2010) UTX mediates demethylation of H3K27me3 at muscle-specific genes during myogenesis. *EMBO J* 29: 1401–1411

Thoreen CC, Chantranupong L, Keys HR, Wang T, Gray NS & Sabatini DM (2012) A unifying model for mTORC1-mediated regulation of mRNA translation. *Nature* 485: 109–113

Wang C, Lee J-E, Cho Y-W, Xiao Y, Jin Q, Liu C & Ge K (2012) UTX regulates mesoderm differentiation of embryonic stem cells independent of H3K27 demethylase activity. *Proc Natl Acad Sci* 109: 15324–15329

Welstead GG, Creyghton MP, Bilodeau S, Cheng AW, Markoulaki S, Young RA & Jaenisch R (2012) X-linked H3K27me3 demethylase Utx is required for embryonic development in a sex-specific manner. *Proc Natl Acad Sci* 109: 13004–13009

1st Revision - Editorial Decision 22nd Jul 2026

Dr. Giovanni Tonon
IRCCS San Raffaele Scientific Institute
Functional Genomics of Cancer Unit, Division of Molecular Oncology
Via Olgettina 60
Milan 20032
Italy

22nd Jul 2026

Re: EMBOJ-2026-123503R
KDM6A Loss Enhances Oxidative Phosphorylation Uncovering Tissue-Level Convergent Evolution

Dear Giovanni,

Thank you for submitting your revised manuscript on KDM6A and oxidative phosphorylation to The EMBO Journal, and please excuse the somewhat delayed re-evaluation. Two of the original referees have now looked at it again, and were overall satisfied with the revisions and improvements. Referee 2 still suggests some points to be better qualified in the discussion section (see comments below), which I would invite you to incorporate during a final round of minor revision. At this stage, please also address the following remaining editorial points:

- Please adjust the order of the manuscript sections, and also make sure to use the correct section headers:
Single title page with complete author information, Abstract, Introduction, Results, Discussion, Methods, Data Availability, Acknowledgements, Disclosure and Competing Interests Statement, References, Main Figure Legends, Tables, Expanded Figure Legends.

- Please double-check to make sure to all relevant funding information in the manuscript is congruent with the info entered into our submission system. Currently missing in the submission system are:
R50CA293837; This seems included in the Comments box: "Several NIH grants", but funding info included in the Comments box could not be extracted by Production team, and therefore all funders should be added to "More Funders" list.

- Please carefully go through the reference list and make sure that all references have their complete citation information - including year, journal name & volume, and page/elocator numbers - such information is currently missing for several of them.

- For preprint citations: Please change in-text reference format according to "preprint: Miller et al, 2025"; and in the reference list, name of the platform plus DOI + preprint label - e.g. "bioRxiv doi: 1234/002.dfj123 [PREPRINT]"

- In the Data Availability section, please include a direct URL to any of the mentioned databases in which data generated in this study have been deposited.

- Please double-check in-text references to figures and tables: FIGURE CALLOUTS: there is a callout for Table 1, but no such table (reference is possibly to the Reagents & Tools table?); missing callout for Fig. 6D, but there is one for Fig. 6N, although there is no such panel in the figure. Finally, Fig. EV1 appears mislabeled as 'Figure S1' in the image file.

- Please upload 'Table EV1' as a spreadsheet rather than a ZIP file, and include its description in an additional 'Legends' tab of the spreadsheet, rather than in a separate 'readme' file.

- Please provide suggestions for a short 'blurb' text prefacing and summing up the conceptual aspect of the study in two sentences (max. 250 characters), followed by 3-5 one-sentence 'bullet points' with brief factual statements of key results of the paper; they will form the basis of an editor-written 'Synopsis' accompanying the online version of the article. Please also upload a synopsis image, which can be used as a "visual title" for the synopsis section of your paper. The image should be in JPG format of 550 pixels wide and 300 pixels high, and please make sure that it remains clear enough even in these modest dimensions.

- Finally, during routine pre-acceptance checks, our data editors have raised the following queries regarding figures, data, and legends; I would appreciate if you briefly answered to them in the cover letter of your final submission, and made the requested text modifications with changes/additions highlighted via the "Track changes" option, to facilitate our final checking:
1. Please note that the box plots need to be defined in terms of minima, maxima, centre, bounds of box and whiskers, and percentile in the legends of figures 1E; 1F; 3E; 5C; 6B; EV-6B.
2. Please note that information related to n is missing in the legends 3H; 4E; 6H,M; EV-1B; EV-2J; EV-3H; EV-4D; EV-6C-E; EV-7A,C.
3. Please note that the error bars are not defined in the legends of figures 3H; 6G,J,M; EV-1D; EV-2J; EV-3A,H; EV-4C,D; EV-

6C-E.
4. Please note that the exact p values are not provided in the legends of 1C-F; 2A,B,C,D,I; 3C,F,G,H; 4A,B,D,E; 5C; 6A-C,E,G,I,J,K,M; 7A,B; EV-1B,F,G; EV-2A-C,G,I,J,M,N,O; EV-3A,D; EV-4A-C; EV-6B,H,J; EV-7A-C.
5. Please indicate the statistical test used for data analysis in the legends of figures 2E; 6H; EV-1D; EV-2H; EV-3C; EV-4H; EV-6F.
6. Please define the white arrows in the legend of figure 5A.

I am returning the manuscript to you for a final round of minor revision, solely to allow you to make these modifications and upload the revised files. Once we will have received them, we should be ready to swiftly proceed with formal acceptance and production of the manuscript.

With kind regards,

Hartmut

Hartmut Vodermaier, PhD
Senior Editor, The EMBO Journal
h.vodermaier@embojournal.org

*** PLEASE NOTE: All revised manuscript are subject to initial checks for completeness and adherence to our formatting guidelines. Revisions may be returned to the authors and delayed in their editorial re-evaluation if they fail to comply to the following requirements. As a first step please read our guidelines for revised submissions:
https://link.springer.com/journal/44318/submission-guidelines#cms-Revised-submissions

1) Every manuscript requires a Data Availability section (even if only stating that no deposited datasets are included). Primary datasets or computer code produced in the current study have to be deposited in appropriate public repositories prior to resubmission, and reviewer access details provided in case that public access is not yet allowed.

2) Each figure legend must specify
- size of the scale bars that are mandatory for all micrograph panels
- the statistical test used to generate error bars and P-values
- the type error bars (e.g., S.E.M., S.D.)
- the number (n) and nature (biological or technical replicate) of independent experiments underlying each data point
- Figures may not include error bars for experiments with n<3; scatter plots showing individual data points should be used instead.

3) Revised manuscript text (including main tables, and figure legends for main and EV figures) has to be submitted as editable text file (e.g., .docx format). We encourage highlighting of changes (e.g., via text color) for the referees' reference.

4) Each main and each Expanded View (EV) figure should be uploaded as individual production-quality files (preferably in .eps, .tif, .jpg formats). For suggestions on figure preparation/layout, please refer to our Figure Preparation Guidelines:
https://media.springernature.com/original/springer-cms/rest/v1/content/27825798/data/v1

5) Point-by-point response letters should include the original referee comments in full together with your detailed responses to them (and to specific editor requests if applicable), and also be uploaded as editable (e.g., .docx) text files.

6) Please complete our Author Checklist, and make sure that information entered into the checklist is also reflected in the manuscript; the checklist will be available to readers as part of the Review Process File.

7) All authors listed as (co-)corresponding need to deposit, in their respective author profiles in our submission system, a unique ORCiD identifier linked to their name. Please see our Guide to Authors for detailed instructions.

8) Please note that supplementary information at EMBO Press has been superseded by the 'Expanded View' for inclusion of additional figures, tables, movies or datasets; with up to five EV Figures being typeset and directly accessible in the HTML version of the article.

9) To facilitate reproducibilty and cross-laboratory adoption of methodologies, please structure the Materials & Methods section as outlined in our guide to authors, including a completed Reagents and Tools Table.

10) Digital image enhancement is acceptable practice, as long as it accurately represents the original data and conforms to community standards. If a figure has been subjected to significant electronic manipulation, this must be clearly noted in the figure legend and/or the 'Materials and Methods' section. The editors reserve the right to request original versions of figures and the

original images that were used to assemble the figure. Finally, we generally encourage uploading of numerical as well as gel/blot image source data.

At EMBO Press we ask authors to provide source data for the main manuscript figures. You will receive a separate email with instructions for providing source data with your revised manuscript, including how to upload and organize the files.

In the interest of ensuring the conceptual advance provided by the work, we recommend submitting a revision within 3 months (20th Oct 2026). Please discuss the revision progress ahead of this time with the editor if you require more time to complete the revisions. Use the link below to submit your revision:

Link Not Available

Referee #2:

The authors have addressed my points satisfactorily. The mechanisms described are better supported by data in the revised manuscript.
I suggest that the authors integrate some of the present challenges and unanswered questions (e.g., the in vivo functional consequences of the tumor mitochondrial phenotype, the pharmacological means of assessing the requirement for OXPHOS/mitochondrial transfer) in the discussion section and temper the language accordingly.

Referee #3:

Thank you for addressing my comments

All minor editorial requests have been addressed by the authors.

EMBO Press Author Checklist

Corresponding Author Name: Giovanni Tonon	
Journal Submitted to: EMBO Journal	
Manuscript Number: EMBOJ-2026-123503	

USEFUL LINKS FOR COMPLETING THIS FORM
The EMBO Journal - Author Guidelines
EMBO Reports - Author Guidelines
Molecular Systems Biology - Author Guidelines
EMBO Molecular Medicine - Author Guidelines

Reporting Checklist for Life Science Articles (updated January
This checklist is adapted from Materials Design Analysis Reporting (MDAR) Checklist for Authors. MDAR establishes a minimum set of requirements in transparent reporting in the life sciences (see Statement of Task: 10.31222/osf.io/9sm4x). Please follow the journal's guidelines in preparing your
Please note that a copy of this checklist will be published alongside your article.

Abridged guidelines for figures
1. Data
The data shown in figures should satisfy the following conditions:
- the data were obtained and processed according to the field's best practice and are presented to reflect the results of the experiments in an accurate and unbiased manner.
- ideally, figure panels should include only measurements that are directly comparable to each other and obtained with the same assay.
- plots include clearly labeled error bars for independent experiments and sample sizes. Unless justified, error bars should not be shown for technical
- if n<5, the individual data points from each experiment should be plotted. Any statistical test employed should be justified.
- Source Data should be included to report the data underlying figures according to the guidelines set out in the authorship guidelines on Data

2. Captions
Each figure caption should contain the following information, for each panel where they are relevant:
- a specification of the experimental system investigated (eg cell line, species name).
- the assay(s) and method(s) used to carry out the reported observations and measurements.
- an explicit mention of the biological and chemical entity(ies) that are being measured.
- an explicit mention of the biological and chemical entity(ies) that are altered/varied/perturbed in a controlled manner.
- the exact sample size (n) for each experimental group/condition, given as a number, not a range;
- a description of the sample collection allowing the reader to understand whether the samples represent technical or biological replicates (including how many animals, litters, cultures, etc.).
- a statement of how many times the experiment shown was independently replicated in the laboratory.
- definitions of statistical methods and measures:
  - common tests, such as t-test (please specify whether paired vs. unpaired), simple χ2 tests, Wilcoxon and Mann-Whitney tests, can be unambiguously identified by name only, but more complex techniques should be described in the methods section;
  - are tests one-sided or two-sided?
  - are there adjustments for multiple comparisons?
  - exact statistical test results, e.g., P values = x but not P values < x;
  - definition of 'center values' as median or average;
  - definition of error bars as s.d. or s.e.m.

Please complete ALL of the questions below.
Select "Not Applicable" only when the requested information is not relevant for your study.

Materials

Newly Created Materials	Information included in the manuscript?	In which section is the information available? (Reagents and Tools Table, Materials and Methods, Figures, Data Availability Section)
New materials and reagents need to be available; do any restrictions apply?	Not Applicable	

Antibodies	Information included in the manuscript?	In which section is the information available? (Reagents and Tools Table, Materials and Methods, Figures, Data Availability Section)
For **antibodies** provide the following information:<br>- Commercial antibodies: RRID (if possible) or supplier name, catalogue number and or/clone number<br>- Non-commercial: RRID or citation	Yes	Reagents and tools table

DNA and RNA sequences	Information included in the manuscript?	In which section is the information available? (Reagents and Tools Table, Materials and Methods, Figures, Data Availability Section)
**Short novel DNA or RNA including primers, probes**: provide the sequences.	Yes	Reagents and tools table

Cell materials	Information included in the manuscript?	In which section is the information available? (Reagents and Tools Table, Materials and Methods, Figures, Data Availability Section)
**Cell lines:** Provide species information, strain. Provide accession number in repository **OR** supplier name, catalog number, clone number, and/**OR** RRID.	Yes	Reagents and tools table
**Primary cultures:** Provide species, strain, sex of origin, genetic modification status.	Yes	Reagents and tools table and Materials and Methods
Report if the cell lines were recently **authenticated** (e.g., by STR profiling) and tested for mycoplasma contamination.	Not Applicable	

Experimental animals	Information included in the manuscript?	In which section is the information available? (Reagents and Tools Table, Materials and Methods, Figures, Data Availability Section)
**Laboratory animals or Model organisms:** Provide species, strain, sex, age, genetic modification status. Provide accession number in repository **OR** supplier name, catalog number, clone number, **OR** RRID.	Yes	Materials and Methods
**Animal observed in or captured from the field:** Provide species, sex, and age where possible.	Not Applicable	
Please detail **housing and husbandry conditions**.	Yes	Materials and Methods

Plants and microbes	Information included in the manuscript?	In which section is the information available? (Reagents and Tools Table, Materials and Methods, Figures, Data Availability Section)
**Plants:** provide species and strain, ecotype and cultivar where relevant, unique accession number if available, and source (including location for collected wild specimens).	Not Applicable	
**Microbes:** provide species and strain, unique accession number if available, and source.	Not Applicable	

Human research participants	Information included in the manuscript?	In which section is the information available? (Reagents and Tools Table, Materials and Methods, Figures, Data Availability Section)
If collected and within the bounds of privacy constraints report on age, sex and gender or ethnicity for all study participants.	Not Applicable	

Core facilities	Information included in the manuscript?	In which section is the information available? (Reagents and Tools Table, Materials and Methods, Figures, Data Availability Section)
If your work benefited from core facilities, was their service mentioned in the acknowledgments section?	Yes	Acknowledgments section

Design

Study protocol	Information included in the manuscript?	In which section is the information available? (Reagents and Tools Table, Materials and Methods, Figures, Data Availability Section)
If study protocol has been **pre-registered, provide DOI in the manuscript**. For clinical trials, provide the trial registration number **OR** cite DOI.	Not Applicable	
Report the **clinical trial registration number** (at ClinicalTrials.gov or equivalent), where applicable.	Not Applicable	

Laboratory protocol	Information included in the manuscript?	In which section is the information available? (Reagents and Tools Table, Materials and Methods, Figures, Data Availability Section)
Provide DOI OR other citation details if **external detailed step-by-step protocols** are available.	Yes	Materials and Methods

Experimental study design and statistics	Information included in the manuscript?	In which section is the information available? (Reagents and Tools Table, Materials and Methods, Figures, Data Availability Section)
Include a statement about **sample size** estimate even if no statistical methods were used.	Yes	Figures
Were any steps taken to minimize the effects of subjective bias when allocating animals/samples to treatment (e.g. **randomization procedure**)? If yes, have they been described?	Not Applicable	
Include a statement about **blinding** even if no blinding was done.	Not Applicable	
Describe **inclusion/exclusion criteria** if samples or animals were excluded from the analysis. Were the criteria pre-established?<br><br>If sample or data points were omitted from analysis, report if this was due to attrition or intentional exclusion and provide justification.	Yes	Source data
For every figure, are **statistical tests** justified as appropriate? Do the data meet the assumptions of the tests (e.g., normal distribution)? Describe any methods used to assess it. Is there an estimate of variation within each group of data? **I**s the variance similar between the groups that are being statistically compared?	Yes	Figures legends and Materials and Methods

Sample definition and in-laboratory replication	Information included in the manuscript?	In which section is the information available? (Reagents and Tools Table, Materials and Methods, Figures, Data Availability Section)
In the figure legends: state number of times the experiment was **replicated** in laboratory.	Yes	Figures legends
In the figure legends: define whether data describe **technical or biological replicates**.	Yes	Figures legends

Ethics

Ethics	Information included in the manuscript?	In which section is the information available? (Reagents and Tools Table, Materials and Methods, Figures, Data Availability Section)
Studies involving **human participants**: State details of **authority granting ethics approval** (IRB or equivalent committee(s), provide reference number for approval.	Not Applicable	
Studies involving **human participants**: Include a statement confirming that **informed consent** was obtained from all subjects and that the experiments conformed to the principles set out in the WMA Declaration of Helsinki and the Department of Health and Human Services Belmont Report.	Not Applicable	
Studies involving **human participants:** For publication of **patient photos**, include a statement confirming that consent to publish was obtained.	Not Applicable	
Studies involving experimental **animals**: State details of **authority granting ethics approval** (IRB or equivalent committee(s), provide reference number for approval. Include a statement of compliance with ethical regulations.	Not Applicable	
Studies involving **specimen and field samples:** State if relevant **permits** obtained, provide details of authority approving study; if none were required, explain why.	Not Applicable	

Dual Use Research of Concern (DURC)	Information included in the manuscript?	In which section is the information available? (Reagents and Tools Table, Materials and Methods, Figures, Data Availability Section)
Could your study fall under dual use research restrictions? Please check biosecurity documents and list of **select agents and toxins** (CDC): https://www.selectagents.gov/sat/list.htm	Not Applicable	
If you used a select agent, is the security level of the lab appropriate and reported in the manuscript?	Not Applicable	
If a study is subject to dual use research of concern regulations, is the name of the **authority granting approval and reference number** for the regulatory approval provided in the manuscript?	Not Applicable	

Reporting

The MDAR framework recommends adoption of discipline-specific guidelines, established and endorsed through community initiatives. Journals have their own policy about requiring specific guidelines and recommendations to complement MDAR.

Adherence to community standards	Information included in the manuscript?	In which section is the information available? (Reagents and Tools Table, Materials and Methods, Figures, Data Availability Section)
State if relevant guidelines or checklists (e.g., **ICMJE, MIBBI, ARRIVE, PRISMA**) have been followed or provided.	Not Applicable	
For **tumor marker prognostic studies,** we recommend that you follow the **REMARK** reporting guidelines (see link list at top right). See author guidelines, under 'Reporting Guidelines'. Please confirm you have followed these guidelines.	Not Applicable	
For **phase II and III randomized controlled trials**, please refer to the **CONSORT** flow diagram (see link list at top right) and submit the CONSORT checklist (see link list at top right) with your submission. See author guidelines, under 'Reporting Guidelines'. Please confirm you have submitted this list.	Not Applicable	

Data Availability

Data availability	Information included in the manuscript?	In which section is the information available? (Reagents and Tools Table, Materials and Methods, Figures, Data Availability Section)
Have **primary datasets** been deposited according to the journal's guidelines (see 'Data Deposition' section) and the respective accession numbers provided in the Data Availability Section?	Yes	Data Availability Section
Were **human clinical and genomic datasets** deposited in a public access-controlled repository in accordance to ethical obligations to the patients and to the applicable consent agreement?	Not Applicable	
Are **computational models** that are central and integral to a study available without restrictions in a machine-readable form? Were the relevant accession numbers or links provided?	Not Applicable	
If publicly available data were reused, provide the respective **data citations in the reference list**.	Yes	References
